# Structure-based identification of a potential non-catalytic binding site for rational drug design in the fructose 1,6-biphosphate aldolase from *Giardia lamblia*

**DOI:** 10.1038/s41598-019-48192-3

**Published:** 2019-08-13

**Authors:** Sara-Teresa Méndez, Adriana Castillo-Villanueva, Karina Martínez-Mayorga, Horacio Reyes-Vivas, Jesús Oria-Hernández

**Affiliations:** 1 Laboratorio de Bioquímica-Genética, Instituto Nacional de Pediatría, Secretaría de Salud, Insurgentes Sur 3700-C, Col. Insurgentes Cuicuilco, Alcaldía Coyoacán, CP 04530 Ciudad de México Mexico; 2 Instituto de Química, Universidad Nacional Autónoma de México, Circuito Exterior, Ciudad Universitaria, Alcaldía Coyoacán, C.P. 04510 Ciudad de México Mexico

**Keywords:** Enzymes, Target identification, Protein sequence analyses

## Abstract

*Giardia lamblia* is the causal agent of giardiasis, one of the most prevalent parasitosis in the world. Even though effective pharmacotherapies against this parasite are available, the disadvantages associated with its use call for the development of new antigiardial compounds. Based on the *Giardia* dependence on glycolysis as a main energy source, glycolytic enzymes appear to be attractive targets with antiparasitic potential. Among these, fructose 1,6-biphosphate aldolase (GlFBPA) has been highlighted as a promising target for drug design. Current efforts are based on the design of competitive inhibitors of GlFBPA; however, in the kinetic context of metabolic pathways, competitive inhibitors seem to have low potential as therapeutic agents. In this work, we performed an experimental and *in silico* structure-based approach to propose a non-catalytic binding site which could be used as a hot spot for antigardial drug design. The druggability of the selected binding site was experimentally tested; the alteration of the selected region by site directed mutagenesis disturbs the catalytic properties and the stability of the enzyme. A computational automated search of binding sites supported the potential of this region as functionally relevant. A preliminary docking study was performed, in order to explore the feasibility and type of molecules to be able to accommodate in the proposed binding region. Altogether, the results validate the proposed region as a specific molecular binding site with pharmacological potential.

## Introduction

Infectious diseases caused or transmitted by parasites are worldwide public-health issues, particularly in underdeveloped countries^1^. Giardiasis, the infestation of the gastrointestinal tract by the protist *Giardia lamblia*, is one of the most common parasitic infections worldwide; the infection rates varies from 2–7% in developed countries to 2–30% in developing countries^2^. Because of its impact on impoverished regions, giardiasis has been included as part of the WHO Neglected Disease Initiative^3^. The clinical manifestations of acute giardiasis include watery diarrhea, epigastric pain, nausea and vomiting; chronic infections can progress to malabsorption syndrome with important weight loss, malnutrition, and failure to thrive in the pediatric population^4^. Current pharmacological therapies for giardiasis include drugs of the nitroimidazole family (metronidazole, tinidazole, ornidazole, secnidazole) or the benzimidazole group (albendazole or mebendazole); in addition, nitazoxanide, furazolidone, quinacrine, chloroquine and paromomycin can be used as alternative treatments^5,6^.

The apparent successful control of *Giardia lamblia* infection is deceptive; the giardiasis infection and treatment still represent important challenges nowadays. For example, recurrence rates are high in endemic areas and first-line therapy fails in up to 20% of cases^6^. In addition, important disadvantages are associated with the use of current therapies; especially the important side effects related to them^6–8^. Finally, clinical and laboratory-induced resistance to current drugs has been demonstrated for this parasite^9–12^. The high prevalence and recurrence of giardiasis in disadvantaged populations, the undesirable side effects of their therapies and the presence of resistant strains indicates that the development of new antigiardiasis therapies is paramount. In this regard, multiple alternative approaches aimed to develop optional therapies for giardiasis, including the use of natural products, vaccine generation, chemical synthesis of new drugs and rational drug design, are currently on progress^1,5–7,9,10,13^.

Rational drug design makes use of the bioinformatical power currently available^1^. For infectious diseases, this approach attempts identifying a biomolecular target which is essential for the infectious agent; this target is then used for the search for compounds that impairs its function. Once a lead compound is identified, it could be used as starting point in the lead optimization process^1^. For *G. lamblia*, glycolytic enzymes have been highlighted as potential pharmacological targets^14,15^. Because *Giardia* has reduced mitochondrion lacking the components of oxidative phosphorylation, glucose degradation via glycolysis serves as a major source of ATP^16^. Therefore, it has been proposed that disrupting the glycolytic pathway via inhibition of their enzymes could hinder the survival of the parasite^14,15^.

The glycolytic enzyme fructose 1,6-bisphosphate aldolase (FBPA) from *Giardia lamblia* (GlFBPA) stands out as one of the most interesting molecular targets for rational drug design against giardiasis. It has been demonstrated that inhibition of the GlFBPA gene transcription in trophozoites by interference RNA yielded no viable organisms^15^, thus validating GlFBPA as a potential drug target. In addition, the phylogenetic distribution of the enzyme supports the plausibility of GlFPBA as a selective target. The fructose 1,6-bisphosphate aldolase family encompass two separate classes of enzymes differing in their enzymatic mechanisms. The class I family employs an active site lysine in Schiff base formation whereas the class II aldolases employ a Zn^2+^ ion as cofactor. Human FBPA belongs to the class I family, whereas GlFBPA belongs to the class II aldolases^17^. Given that both families do not share any structural, functional or phylogenetic relationship^18^, it has been envisioned that designing drugs that selectively inhibits the parasitic enzyme without affecting the human enzyme is feasible^15^. In order to unravel the determinants of catalysis and substrate recognition that could direct the discovering of specific enzyme inhibitors, the crystal structure of GlFBPA has been obtained in the ligand-free state and in complex with the substrate D-fructose 1,6-bisphosphate (F1,6P), the transition state analog phosphoglycolohydroxamate or the competitive inhibitor tagatose-1,6-bisphosphate^15,19^. The analysis from the GlFBPA crystal structures indicates a complex network of residues involved in substrate discrimination, including amino acids within the 1st, 2nd and higher level shells surrounding the ligand^15,19^.

The structural features of the GlFBPA active site that govern ligand binding and the differences in the catalytic mechanisms of class I and class II aldolases have been exploited to design selective competitive inhibitors of the *G*. *lamblia* enzyme^20^. However, in the kinetic context of metabolic pathways, competitive inhibitors may have limited pharmacological potential, as has been previously stressed^21–23^. Therefore, we hypothesize that inhibitors of GlFBPA acting at a non-active binding site could be better prospects to develop successful antigiardial therapies.

In this work, we performed an *in silico* analysis of GlFBPA to identify a non-catalytic binding site with potential to be used as molecular target for subsequent drug design. The effects caused by modification of the selected region were experimentally tested by site directed mutagenesis. The results indicate that the alteration of this binding site disturbs the substrate affinity, the catalytic activity and the stability of GlFBPA. A computational automated search of binding sites and preliminary docking studies builds on the potential of this region as functionally relevant, thus supporting the proposed region as a specific molecular binding site with pharmacological potential.

## Material and Methods

### Selection of the target region

A complete description of the method used for selection of the potential target binding site in GlFBPA is provided in the Results section (see below). Bioinformatics tools used in the process are described next. GlFBPA related sequences were retrieved with the Protein BLAST algorithm^24^ from the curated Reference Sequence collection (RefSeq) at the National Center for Biotechnology Information^25^. Multiple sequence alignment of FBPA sequences was generated with the Clustal_X 2.1 package^26^ by using the Gonnet 250 matrix^27^. Crystal structures of GlFBPA were analyzed with the Discovery Studio 3.1 visualizer from Accelrys. Solvent accessible surface areas were calculated with the GETAREA server^28^. Sequence logo was performed with WebLogo3^29^.

### Materials and general procedures

Analytical grade chemicals were acquired from Sigma-Aldrich; molecular biology reagents were purchased from New England BioLabs and Invitrogen. Oligonucleotide synthesis and automated DNA sequencing were performed at the Unidad de Biología Molecular, Instituto de Fisiología Celular, UNAM. Protein concentration was determined by the Bradford method or by absorbance at 280 nm for pure GlFBPA considering ∈_280_ = 18910 M^−1^ cm^−1^. The enzymatic activity of GlFBPA was measured at 25 °C in the direction of F1,6P cleavage by a spectrophotometric assay coupled to triosephosphate isomerase and glycerol-3-phosphate dehydrogenase^15^. Standard reaction mixture contains 0.5 mM F1,6P, 0.2 mM NADH, triosephosphate isomerase and glycerol-3-phosphate dehydrogenase (10 and 5 units, respectively) in 50 mM Hepes pH 7.5; the reaction was initiated by the addition of WT GlFBPA or mutants. SDS-PAGE (12%) was performed according to Shägger and von Jagow^30^.

### Isolation and cloning of the *glfbpa* gene

*G*. *lamblia* trophozoites (WB strain) were cultured at 37 °C for 72 hours in TYI-S-33 medium as described^31^. From these cells, genomic DNA was obtained by standard extraction by phenol–chloroform. Amplification of the *glfbpa* gene was accomplished by polymerase chain reaction (PCR) by using the following oligonucleotides: sense 5′-CATATGCCTCTCTGCACTCTG-3′ and antisense 5′-GGATCCTTACTTGTACCATGC-3′; which introduces *NdeI* and *BamHI* restriction sites (underlined) at the 5′ and 3′ ends, respectively. The PCR reaction mixture contained 100 ng of genomic DNA, 0.5 μM oligonucleotides, 1.5 mM MgCl_2_, 0.2 mM dNTPs and 2.5 units of Taq Polymerase (BD Biosciences); run conditions were: 94 °C for 5 min, 30 cycles of 1 minute at 94 °C, 1 minute at 57 °C and 1 minute at 72 °C plus a final step at 72 °C by 5 minutes. The amplified DNA fragment (980 bp) was purified from a 1.5% agarose gel with the Wizard SV Gel and PCR Clean-UP System (Promega), cloned into the pCR 2.1 vector as recommended (Invitrogen) and used to transform *E*. *coli* TOP10F competent cells. The cloned fragment was subcloned into the pET3a vector after digestion with *NdeI* and *BamHI* and used to transform *E*. *coli* TOP10F competent cells. The insert from an ampicillin-resistant clone was completely sequenced from T7 promoter to T7 terminator to confirm the correctness of the final construction; this was named pET3a-*glfbpa* and used to transform *E*. *coli* BL21(DE3)pLysS cells.

### Site directed mutagenesis

The R259A and D278A GlFBPA mutants were constructed over the pET3a-*glfbpa* construction by PCR site directed mutagenesis. The mutagenic oligonucleotides were, for R259A, Fw 5′-TGACTCCGCGATGGCCA-3′ and Rv 5′-TGGCCATCGCGGAGTCA-3′; and for D278A, Fw 5′-GAGAAATTCGCGCCGCGC-3′ and Rv 5′-GCGCGGCGCGAATTTCTC-3′ (mutations underlined). For mutagenesis reactions, external T7 promoter and terminator oligonucleotides (Novagen) were used. PCR conditions were the same as for gene amplification. The double mutant R259A/D278A was constructed with the D278A mutagenic oligonucleotides over the D259A template under the same protocol. In all cases, successful mutagenesis was confirmed by automated DNA sequencing of the complete genes.

### Expression and purification of WT GlFBPA and mutants

The expression and purification procedure was identical for the WT GlFBPA or the mutants. The recombinant *E*. *coli* strains were grown at 37 °C and 200 rpm in 1 liter of LB medium supplemented with 100 mg/L of ampicillin until an OD of 0.8 was reached. Expression was induced by the addition of 0.4 mM isopropyl 1-thio-β-D-galactopyranoside and conducted overnight at 30 °C. Cells were harvested by centrifugation, disrupted by sonication at 4 °C in 50 mM Tris-HCl pH 8.0, 50 mM NaCl, 2 mM PMSF, 10% (v/v) glycerol and centrifuged at 10,000 *g* and 4 °C by 30 min. Supernatant was precipitated at 33% of (NH_4_)_2_SO_4_ and 4 °C by one hour and centrifuged at 10,000 *g* and 4 °C by 30 minutes. The supernatant was collected, bring to 75% of (NH_4_)_2_SO_4_, incubated at 4 °C by one hour and centrifuged at 10,000 *g* and 4 °C by 30 minutes. The pellet was resuspended in 50 mM Tris-HCl pH 8.0, 10% (v/v) glycerol and dialyzed overnight against 1 liter of the same buffer. Protein was applied to a Q-Sepharose column equilibrated in 50 mM Tris-HCl pH 8.0, 10% (v/v) glycerol; protein elution was accomplished by a linear gradient of NaCl from 0 to 500 mM in 50 mM Tris-HCl pH 8.0, 10% (v/v) glycerol. The fractions with the higher specific activity were pooled, concentrated to 2 mL and applied to a Superdex-200 column equilibrated and developed in 50 mM Tris-HCl pH 8.0, 150 mM NaCl and 10% (v/v) glycerol. Fractions with the higher specific activity were pooled, concentrated to ~10 mg/ml and stored in 50% (v/v) glycerol at −76 °C.

### Characterization of WT GlFBPA and mutants

For the functional characterization of WT GlFBPA and mutants, the kinetic constants of each enzyme were determined by collecting initial velocity data at F1,6P concentrations ranging from 1 to 800 μM (depending on the affinity of each enzyme); the experimental datasets were fitted to the Michaelis-Menten equation (*v* = *V*_*max*_ · S/[K_m_ + S]) by non-linear regression calculations.

The protein stability was characterized in first instance by following the thermal induced unfolding of WT GlFBPA and mutants (0.1 mg/mL in 25 mM phosphate buffer pH 7.4) as the change in the circular dichroism signal at 222 nm in temperature scans from 25 to 90 °C with increments of 20 °C/hour; the fraction of unfolded protein and melting temperature (T_m_) values were calculated as reported^32^. For thermal inactivation assays, protein samples at 0.1 mg/mL in 50 mM Hepes pH 8.0 were incubated for 20 minutes at temperatures ranging from 25 to 70 °C; at the end of the incubation period, samples were cooled in an ice-water bath for 5 minutes whereupon aliquots were withdrawn to determine the residual activity under standard conditions. Urea induced inactivation was assayed by incubating 0.1 mg/mL of GlFBPA in 50 mM Hepes pH 8.0 at 37 °C for 2 hours at urea concentrations ranging from 0.1 to 4 M; at the end of the incubation, the residual activity was measured. The maximum urea concentration on reaction samples was 40 mM, which do not have effect on the GlFBPA activity nor in the coupling enzymes.

The structural characterization of WT GlFBPA and mutants was performed spectroscopically. Far-UV circular dichroism assays were performed in a Jasco J-810 spectropolarimeter equipped with a thermostated Peltier controlled cell holder in a quartz cell with a path length of 0.1 cm. Spectral scans at 25 °C were performed from 190 to 260 nm at 1 nm intervals with 0.1 mg/mL of protein in 25 mM phosphate buffer pH 7.4. Protein intrinsic fluorescence assays were performed in a Perkin-Elmer LS-55 fluorescence spectrometer. Emission fluorescence spectra from 310 to 500 nm were recorded after excitation at 280 nm with 0.1 mg/mL of protein in 50 mM Hepes pH 8.0 at 25 °C. The binding of 8-anilinonaphthalene 1-sulphonate (ANS) to GlFBPA (WT or mutants) was analyzed at 25 °C in 50 mM Hepes pH 8.0 by following the fluorescence emission spectra from 420 to 600 nm after excitation at 395 nm; the final concentrations of ANS and protein were, respectively, 150 μM and 0.3 mg/mL. For all spectroscopic assays, blank spectra without protein were subtracted from the experimental ones.

### Identification of potential target binding sites by computational automated search and docking studies

For the automatic search of putative binding sites, we used the crystal structure with highest resolution available in the Protein Data Bank (PDBID: 2ISW). The protein was prepared in MOE 2019.01 (Chemical Computing Group), using the protein preparation wizard. The preparation consisted of the addition of hydrogen atoms at pH = 7.4 (assigning protonation states, when required), energy minimization using MMFF94x force field, assignment of tautomeric species and calculation of partial atomic charges, using standard parameters. Chirality was maintained as well as crystallographic water molecules. Then potential binding sites were searched in the entire protein surface using SiteFinder (MOE). The methodology is described in the literature^33–35^. Briefly, regions that have tight atomic packing and are not too exposed to the solvent are selected. Then the potential sites are classified as hydrophobic or hydrophilic, followed by the selection of accessible regions identified by alpha spheres that satisfy a modified Delaunay triangulation definition. Potential binding sites correspond to clusters of alpha spheres, identified by a single-linkage clustering algorithm, with hydrogen bonding spots and at least one hydrophobic alpha sphere.

Docking studies were performed using MOE 2019.01. The binding site searched consisted of the predicted amino acids found with the SiteFinder tool (Table S1). A diverse subset of 50 molecules from Drugbank were docked on the proposed binding site. Docking settings were maintained to default values, except for the exhaustiveness of the conformational search, which was increased to 40.

## Results

### Computational procedure

#### Identification of a novel potential pharmacological binding site in GlFBPA

In the search for a druggable target binding site in GlFBPA, three main characteristics were pursued: (i) The selected region should be important for the structure or the function of the protein, such that its modification exert a negative effect on the enzyme. (ii) The selected region should be different to the active site, to avoid developing a molecule acting as a competitive inhibitor. (iii) The selected region should be accessible to external molecules, such that a small molecule (as a drug) can bind on it. In order to find a binding site on the protein that meet these conditions, the procedure described below was applied.

The selected region must be important for the structure or the function of the protein: We hypothesized that protein residues that are conserved through evolution must play a main role on the structure or function of GlFBPA, such that modification of these residues is precluded. In order to determine conserved amino acids, related sequences to GlFBPA were retrieved from the RefSeq Database at the NCBI^25^ by using the Protein BLAST algorithm^24^. The input sequence was that of GlFBPA whereas the selected output was the first 1000 sequences with the high *E*-scores. To avoid sequence redundancy, only two species from each genus were selected from this dataset; 287 sequences were thus used for further analysis. With these sequences, a progressive multiple sequence alignment was calculated with the Clustal_X package^26^ by using the Gonnet 250 matrix^27^. The generated alignment (Supplementary Fig. S1), was used to search for strictly (100%) conserved residues; 23 amino acids fulfill this condition and are shown in Table 1.

**Table 1:** Selected amino acids in each stage of the procedure outlined in the Results section.

Conserved residues		Non-active site residues		Solvent-accesible residues
N24	G175	N24	E143	D105
E29	H178	E29	A171	G175
D83	G179	G85	G175	D278
H84	V208	M103	V208	
G85	H210	D105	G235	
M103	G211	E133	K251	
D105	G235	E135	D278	
E133	K251	G137		
E135	N253			
G137	D278			
E143	R280			
A171				

The selected region must be different to the active site: It is expected that amino acids contributing to substrate binding or catalysis must be strictly conserved in the GlFBPA family. Therefore, in order to discard active site residues, the crystal structures of GlFBPA obtained in the presence of the substrate F1,6P (PDB code 3GB6), was examined. Eight conserved residues interacting with the substrate^19^ were discarded of those initially selected (Table 1); the remaining 15 residues were further analyzed.

The selected region must be accessible to external molecules: A fundamental requirement for a potential pharmacological region is its accessibility for the binding of external molecules. We envisioned that residues on the surface of the protein can fulfill this requirement, in contrast to buried residues which would be less accessible to potential drugs. Therefore, we search for solvent exposed residues within the conserved, non-active site residues chosen. Only residues D105, G175 and D278 were at least partially solvent-exposed and therefore, potentially accessible to external molecules.

**Figure 1:**
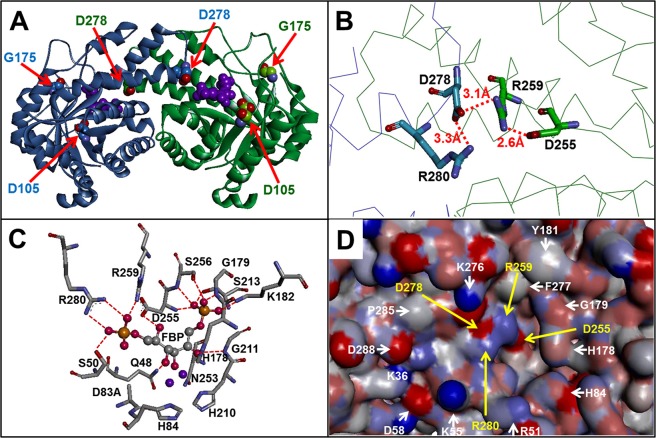
Structural analysis of GlFBPA according to the described method. (**A**) Overall view of D105; G175 and D278 on the structure of GlFBPA. Each subunit of the dimer is shown in green and blue. Selected residues are shown as space-filling models and signaled by arrows. The active site is pointed out by the presence of F1,6P, which is shown as a purple space filling model. (**B**) Close-up view of the interfacial salt bridge network formed by D278 and R280 of one subunit and D255 and R259 of the adjacent monomer. (**C**) Active site residues of GlFBPA; amino acids are shown in stick models and F1,6P as a ball and stick model (FBP), zinc ions are purple balls; key electrostatic interactions between the protein and the substrate are shown as red-dashed lines. (**D**) Surface model of the binding site region: residues selected by the described method are pointed by yellow arrows, whereas amino acids lining the crevice in which this are positioned are signaled by white arrows.

An overview of the three selected amino acids attracted immediate attention over D278. This residue is positioned in the interface region of the protein, and in proximity to the active site (Fig. 1A). A close examination of the D278 residue showed that it is part of an interfacial salt bridge network comprising R280 and D278 of one monomer and R259 and D255 of the adjacent subunit (Fig. 1B). In addition, D278 has a central role coordinating R259 and R280 (Fig. 1B), both binding residues for F1,6P (Fig. 1C). Finally, the surface analysis of GlFBPA showed that all these residues (Fig. 1D, yellow arrows), are superficially positioned in a crevice lined mainly for polar and charged residues (Fig. 1D, white arrows), thus defining a potential target binding site.

In summary, the procedure outlined above allowed us to identify D278 as a highly conserved, non-active site residue accessible to external molecules. The structural analysis of this particular residue unveils a central position within the protein interface and with relevant connections with active site residues, thus suggesting an important role of this region on the structure and function of GlFBPA. Finally, the solvent accessible surface analysis of these amino acids (D255, D278, R259, and D278) indicates that these form a plausible target binding region with pharmacological potential.

In order to experimentally test the relevance of the proposed region for GlFBPA, residues D278 and R259, the central residues of the interfacial salt bridge network (Fig. 1B) were mutagenized. It was hypothesized that mutagenesis of these residues could provoke deleterious effects on the function, the structure or the stability of GlFBPA, thus validating the selected region as a druggable target binding site. The characterization of the functional, structural and stability properties of the WT GlFBPA and the D278A, R259A and R259A/D278A mutants is described below.

### Experimental validation

#### Production of the WT GlFBPA and mutants

**Figure 2:**
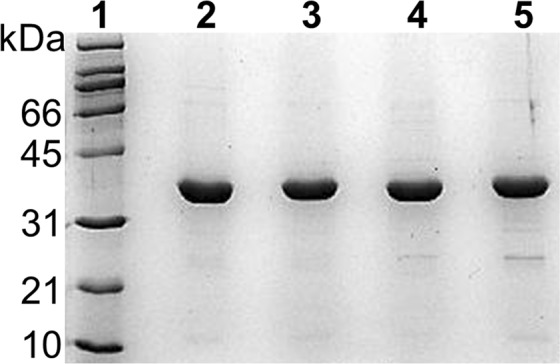
Purification of WT GlFBPA and mutants. Samples of purified proteins were loaded in 12% SDS-PAGE and stained with Coomassie blue. Lane 1, molecular weight standards. Lane 2, WT protein. Lane 3, R259A mutant. Lane 4, D278A mutant. Lane 5, R259A/D278A double mutant. Gel image was cropped and brightness and contrast were slightly modified for clarity. Full-length gel without modifications is shown as Supplementary Fig. S2.

The *glfbpa* gene was isolated from genomic DNA by amplification with specific primers and cloned to the pET-3a vector for heterologous expression of the protein. The sequence of the amplicon was compared with the deposited sequence of GlFBPA (GiardiaDB locus GL50803_11043), showing 100% of identity. The R259A, D278A and R259A/D278A mutants were constructed by PCR mutagenesis and verified by full sequencing of the genes. Recombinant proteins, WT and mutants, were efficiently produced; in all cases the final purity of the samples was higher than 95% as indicated by SDS-PAGE (Fig. 2), with final yields around 50 mg of protein per liter of culture.

#### Functional characterization of the WT GlFBPA and mutants

As suggested by the structural analysis of the protein, it was expected that mutations affected the catalytic function of GlFBPA, therefore, the kinetic constants for the cleavage reaction of F1,6 P were obtained for the WT and the mutant enzymes (Table 2). For the WT enzyme, the kinetic constants closely agree with those previously obtained for recombinant GlFBPA^15^. Each of the mutations exerted differential effects over the kinetic properties of GlFBPA (Table 2). In comparison with the WT enzyme, the *k*_*cat*_ of the D278A mutant was reduced by a third, whereas the K_m_ value was almost doubled; in contrast, for the R259A enzyme, a 14-fold reduction of the *k*_*cat*_ was observed, along with a 160-fold increase for the K_m_. Remarkably, in the D278A/R259A mutant, an intermediate effect between that provoked by single mutations was observed; the *k*_*cat*_ for the double mutant was only 4-times lower than the *k*_*cat*_ of the WT enzyme, whereas the K_m_ was 6-times higher. Consequently, the catalytic efficiency (the *k*_*cat*_/K_m_ ratio) for the D278A mutant was reduced to 35% with respect to the WT enzyme, whereas for the for the R259A protein was only 0.0004%; as observed, the R259A/D278A show an intermediate behavior with a catalytic efficiency of 4% with respect to the WT enzyme (Table 2). The results indicate that in each mutant the substrate binding and the catalysis is affected, albeit at different extents; however, in all cases, mutations decreased the catalytic efficiency of the enzyme.

**Table 2:** Kinetic constants of WT GlFBPA and mutants.

Protein	*k*_*cat*_ (seg^−1^)	K_m_ (μM)	*k*_*cat*_/K_m_ (seg^−1^ M^−1^)
WT	4.3 ± 0.47	1.8 ± 0.6	2.4 × 10^6^
R259A	0.3 ± 0.04	287.5 ± 61.7	866.2
D278A	2.9 ± 0.32	3.4 ± 0.5	8.5 × 10^5^
R259A/D278A	1.1 ± 0.17	11.2 ± 2.6	9.9 × 10^4^

Values are the mean ± SD for three independent experiments. The previously reported kinetic constants for the WT GlFBPA were *k*_*cat*_ 3.55 seg^−1^, K_m_ 1.7 μM and *k*_*cat*_/K_m_ 2.1 × 10^6^ seg^−1^ M^−1^ ^15^.

#### Stability characterization of the WT GlFBPA and mutants

**Figure 3:**
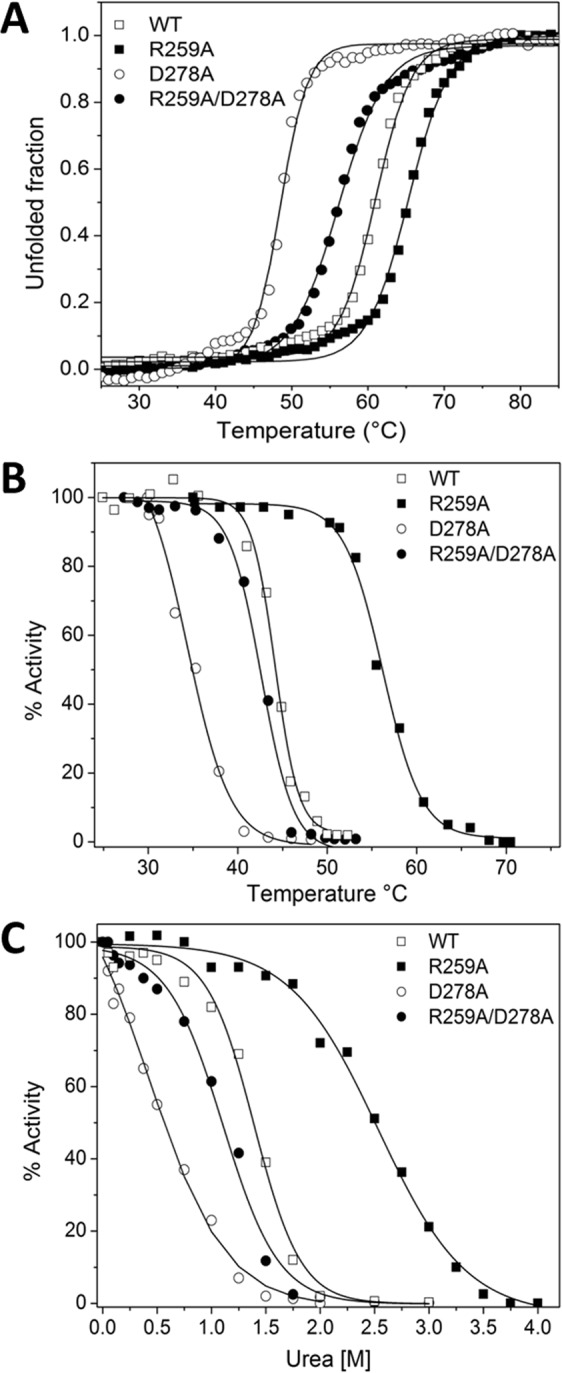
Stability of WT GlFBPA and mutants. (**A**) Thermal induced denaturation of WT GlFBPA and mutants. The circular dichroism signal at 222 nm was recorded in thermal scannings from 25 to 90 °C. The experiment is representative of duplicate experiments. T_m_ values are shown in Table 3. (**B**) Thermal inactivation of WT GlFBPA and mutants. Protein samples were incubated by 20 minutes at the temperature indicated in the abscissa axis afterward the residual activity was determined. The experiment is representative of duplicate experiments. T_1/2_ values are shown in Table 3. (**C**) Urea induced inactivation of WT GlFBPA and mutants. Protein samples were incubated by 2 hours at 37 °C at the urea concentrations indicated in the abscissa axis afterward the residual activity was determined. The experiment is representative of duplicate experiments. C_m_ values are shown in Table 3.

In view of the structural location of the selected residues, the effect of the mutations over the stability of GlFBPA was analyzed. The thermal induced denaturation of the WT GlFBPA and mutants followed by circular dichroism showed that mutations induce differing effects on the stability of the protein (Fig. 3A and Table 3). As hypothesized, the D278A mutation diminishes the thermal stability of the enzyme, as indicated by the decrease of 13.1 °C in the T_m_ (the temperature at the midpoint of the denaturation) with respect to the WT enzyme (60.9 °C and 47.8 °C for the WT and the mutant, respectively). Surprisingly, the R259A mutation displayed the opposite effect; the T_m_ of the mutant increased by 4.5 °C in comparison with the WT enzyme (60.9 °C for the WT enzyme and 65.4 °C for the mutant). As observed for the effect of mutations over the kinetic constants of GlFBPA, the R259A/D278A mutant displays an intermediate behavior; the T_m_ of this mutant (56.0 °C) is located between the T_m_ of the R259A mutant (65.4 °C) and those of the D278A mutant (47.8 °C).

**Table 3:** Stability parameters of WT GlFBPA and mutants.

Protein	T_m_ (°C)	T_1/2_ (°C)	C_m_ (M)
WT	60.9	44.2	1.4
R259A	65.4	56.2	2.5
D278A	47.8	34.4	0.6
R259A/D278A	56.0	42.6	1.1

T_m_ is the temperature at the denaturation midpoint as indicated by thermal induced denaturation followed by circular dichroism. T_1/2_ is the temperature that decreases the activity of the enzyme at a half after 20 minutes incubation. C_m_ is the concentration of urea that reduces 50% of the enzyme activity after incubation by 2 hours at 37 °C. Values are the average of duplicate experiments with S.D. less than 5%.

A similar behavior was observed when the stability of these proteins was investigated by thermal induced inactivation assays (Fig. 3B and Table 3). The T_1/2_ (the temperature at which the enzyme loses 50% of its original activity) was 44.2 °C, 34.4 °C, 56.2 °C, and 42.6 °C for the WT, the D278A, the R259A and the R259A/D278A proteins, respectively. In concordance with the thermal denaturation assays, it was observed that the D278A mutant is prone to heat-inactivation, whereas the R259A mutation protects the enzyme activity. Again, in the double mutant R259A/D278A, the effect is intermediate between those observed for the single mutations.

The changes on the stability of GlFBPA were further confirmed by urea induced inactivation assays (Fig. 3C and Table 3). The C_m_ (the urea concentration that reduces the enzyme activity at a half) was 1.4 M for the WT enzyme, 0.6 M for the D278A enzyme, 2.5 M for the R259A mutant and 1.1 M for the double mutant R259A/D278A. This indicates that the same behavior observed in the thermal denaturation and inactivation assays is preserved; that is, D278A is destabilizing, R259A is stabilizing and R259A/D278A have an intermediate behavior.

#### Structural characterization of the WT GlFBPA and mutants

**Figure 4:**
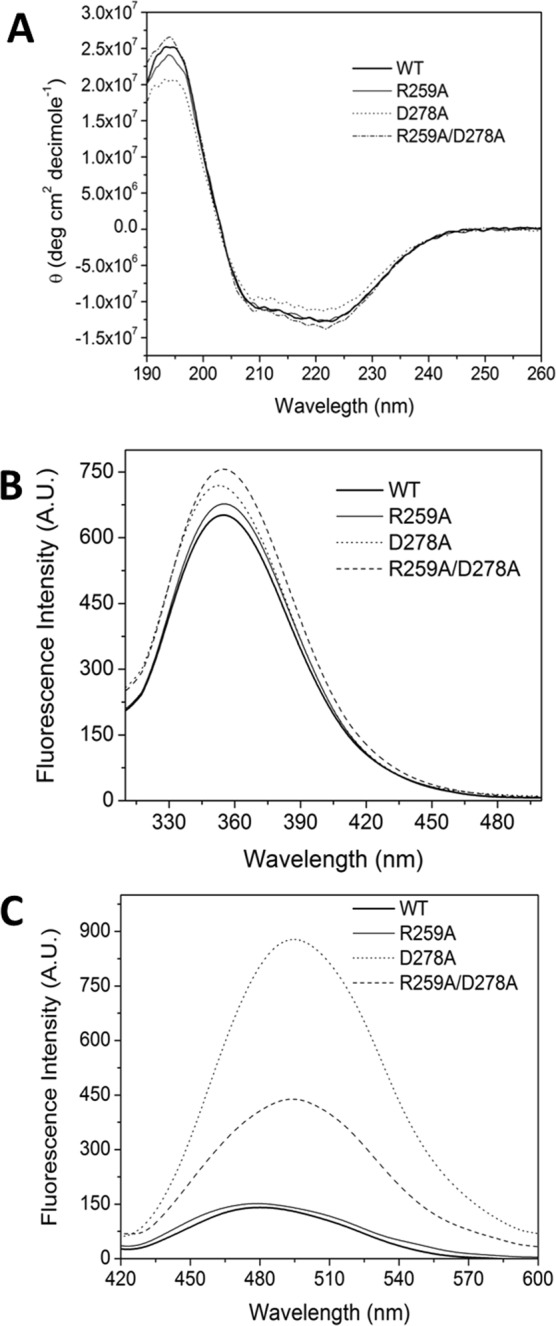
Spectroscopic characterization of WT GlFBPA and mutants. (**A**) Far-UV circular dichroism spectra of WT GlFBPA and mutants. The spectral scan of each protein sample was performed for 190 to 260 nm at 1 nm Intervals. Each spectrum is the average of three replicated scans in duplicate assays. (**B**) Intrinsic fluorescence spectra of WT GlFBPA and mutants. The spectral scan of each protein sample was acquired from 310 to 500 nm after excitation at 280 nm. Each spectrum is the average of three replicated scans in duplicate assays. (**C**) ANS fluorescence spectra in the presence of WT GlFBPA and mutants. The spectral scan of each protein sample in the presence of ANS was acquired from 420 to 600 nm after excitation at 395 nm. Each spectrum is the average of three replicated scans in duplicate assays. In all experiments, blanks (buffer without protein) were subtracted from experimental data.

In order to gain insights into the structural changes that could explain the effects of mutations on GlPBPA, the WT protein and the mutants were spectroscopically-characterized. The far-UV circular dichroism spectrum of the R259A and the R259A/D278A mutants closely resembled the spectrum of the WT enzyme, whereas in the D278A mutant the signal decreased slightly (Fig. 4A). In consonance, the intrinsic protein fluorescence data showed minor changes; for all three mutants, a small increase of the maximal fluorescence intensity (5–16%) with respect to the WT enzyme was observed, which was accompanied by minimal changes on the λ_max_ of the D278A mutant (2–3 nm) (Fig. 4B). From this set of experiments, no clear correlation between mutations and conformational changes could be concluded. In order to extend the structural studies, the binding of ANS to the WT and mutant proteins was analyzed (Fig. 4C). ANS is an amphiphilic fluorescent probe whose fluorescence increases after binding to hydrophobic surfaces, thus, it can be used to monitor protein conformational changes, mainly, exposure of hydrophobic regions. The results show that in the presence of the WT and the R259A mutant ANS shows a minimal fluorescence; in contrast, in the D278A mutant a 6-fold increase of the ANS fluorescence is observed. In the double mutant R259A/D278A the fluorescence increase is less pronounced, 3-fold, albeit it is still over the basal fluorescence of ANS with the WT enzyme. Altogether, the spectroscopic data indicates that minimal structural changes occurs in the mutated proteins, but in the D278A enzyme an increase on the exposure of hydrophobic surface can be inferred. In addition, in the double mutant, the behavior is in between of the individual mutants.

### Computational analysis

#### Automatic identification of target binding sites and docking studies

An automatic search of binding sites, using the SiteFinder tool implemented in MOE 2019.01, was undertaken. The search, performed on the entire protein and without any bias, rendered 27 potential binding sites, the list of residues forming each potential binding site is provided in the Supplementary Table S1. Among the 27 potential binding sites obtained, Site 1 involved residues from each monomer: A22, N24, G48, S50, D83, H84, S177, H178, G179, K182, H210, G211, S212, S213, N253, V254, D255, S256, R259, and R280 from the second monomer. Notably, three residues pointed out as a potential binding site by the approach outlined here (Fig. 1D; D255, R259 and R280) are contained in this region. Thus, combining experimental data and theoretical predictions of binding sites strengths the selection for druggable regions.

Preliminary docking studies were performed to have a first assessment on the type of molecules able to accommodate in the proposed binding site. A set of 50 diverse molecules from Drugbank were docked in the proposed binding site, rendering computational hits. Relevant molecules are shown in the Supplementary material, Figs S3 and S4. This binding region covers the vicinity of the experimentally tested and scored first within the predicted binding pockets. Interestingly, even though D278 is not listed in this site, it is in close proximity to this site to make contacts with ligands located in this region. For example, among the molecules evaluated, three of them (CAS numbers 51-75-2, 26675-46-7, 58-39-9) interact directly with residue D278 (Supplementary Fig. S3), which as described above is key on the proposal of the binding site presented in this work. The binding site is able to accommodate polar small molecules. Interestingly, molecule 58-39-9 makes CH-π interactions as well as hydrogen bonds (Supplementary Figs S3 and S4). In addition, structural modifications to this molecule could be envisioned to promote additional interactions, in order to increase binding affinity. For example, hydrogen bonds could be promoted between R259 and a potential carbonyl group incorporated in the benzyl group of molecule 3. Based on these results, virtual screening efforts targeting this region should incorporate small highly polar molecules.

## Discussion

Giardiasis is one of the most prevalent parasitosis in the world, affecting especially impoverished populations^2^. In the search for new pharmacological options against it, rational drug design stands as an appealing strategy. In infective diseases, rational drug design involves the selection of an essential biological target and the search for a molecule that can interfere with its biological function, thus decreasing the viability of the pathogen. In this respect, the glycolytic enzyme FBPA has been largely proposed as pharmacological target against bacteria^36–43^, fungus^44,45^ and protozoan^46–50^, including *G*. *lamblia*^15,19,20^. In virtually all cases, current efforts have been intended to target the catalytic site, expecting to exploit the structural differences between the active sites of the host and the parasite enzymes. In this respect, two points deserve consideration: (i) even when structural differences can exists between active sites, it cannot be overlooked that both sites are evolutionarily optimized to bind the same ligands (substrates and products); therefore, it is not improbable that structural analogs bind to both sites with low selectivity. (ii) It is expected that substrate or product analogs acts as competitive inhibitors; however, in the kinetic context of open systems, competitive inhibitors may yield disappointing results as therapeutic agents, as previously detailed^21–23^. Substrate and inhibitor molecules are mutually competitive, such that the increase in the substrate concentration (caused by the blockage of the inhibited enzyme) may overcome ultimately the effects of the competitive inhibitor^21–23^. In this connection, the search for inhibitor binding sites, different from the active site, seems to be more attractive.

In the search for non-catalytic binding sites, diverse options have been envisioned. Protein interfaces^51^ or allosteric sites^52^ have been proposed, but these options are of course limited to multimeric proteins or those possessing heterotropic modulators. On the other hand, if *de novo* binding sites are searched, experimental approaches like Multiple Solvent Crystal Structures (MSCS)^53^ or Computational Solvent Mapping^54^ try to undercover not previously identified binding sites on the surface of the proteins. Unfortunately, neither of these two last approaches can ensure that such binding sites may provoke structural or functional effects over the selected proteins.

In contrast, the procedure outlined in this work allowed us to identify D278 as a conserved, non-active site residue accessible to external molecules (Table 1), which along with residues D255, R259, and D278 are involved in interfacial subunit contacts (Fig. 1B) and in the coordination of active site residues (Fig. 1C). In addition, the surface analysis of GlFBPA show that these amino acids are positioned in a crevice lined mainly by polar and charged residues (Fig. 1D) which would be used as molecular cast for docking studies. The druggability of this region was tested by site directed mutagenesis, demonstrating that its modification exerts detrimental effects on the function (Table 2), the stability (Fig. 3 and Table 3) or the structure (Fig. 4) of GlFBPA. It is hypothesized that an organic molecule designed to binds to the proposed binding site could modify the arrangement of this region, exerting similar effects to those showed by mutagenesis. The results obtained from the automated search of binding sites further supported the potential of this region as functionally relevant, based on the disruption of function from the experiments obtained from the mutants and based on geometric considerations from modeling studies. It is important to note that using solely the automated search would require the exploration of 27 different regions. However, the knowledge-based strategy used in this work, allowed the identification of a relevant region, confirmed by the automated search. The consistency between these two approaches (experimental and theoretical) on the identification of this region as relevant mutually supports the findings. The ability of this region to accommodate small molecules was shown via docking studies. Analysis of this preliminary study provided initial knowledge of the type of molecules amenable for the proposed binding site. A virtual screening campaign will include the search of potential ligands on multiple potential binding sites, with the aim of identifying those that are predicted to preferentially bind the proposed binding site. An exhaustive search for ligands using virtual screening will provide computational hits which will potentially modulate the activity of this enzyme; progress on this direction are under way.

In regard to the molecular explanation of our results, it is presumed that the detrimental effects of mutagenesis can be linked to the disruption of the interfacial salt bridge network and to the derangement of active site residues, but conclusive atomic-level structural evidence is needed to support these conjectures. In this sense, the contrary than expected stabilizing effect of the R259A mutant remain as an open question. Similarly, the compensatory effect of the double mutation cannot be explained at this point. It can be argued that in single mutants, hidden unbalanced charges inside the protein interface destabilize and alter the structure of the protein, whereas in the double mutant this unbalance is attenuated, thus decreasing the negative effects of individual mutations. Efforts to obtain the crystal structure of mutant proteins are currently underway to clarify all these points. Nonetheless, the evidence presented here supports the feasibility of the proposed region as a non-catalytic binding site amenable to be used as docking site to perform a virtual screening for the selection of non-competitive GlFBPA inhibitors with antigiardial potential.

## Supplementary information

Dataset 1
