# Supplementary material for: Structure-based identification of a potential non-catalytic binding site for rational drug design in the fructose 1,6-biphosphate aldolase from Giardia lamblia
Source: Sci Rep. 2019 Aug 13;9:11779. doi: 10.1038/s41598-019-48192-3 (PMC6692403; doi:10.1038/s41598-019-48192-3)
Supplement: Supplementary file 1 — Dataset 1 [file 41598_2019_48192_MOESM1_ESM.pdf]

Supplementary material

Structure-based identification of a potential non-catalytic binding site for rational drug design in the fructose 1,6-biphosphate aldolase from *Giardia lamblia*

Sara-Teresa Méndez, Adriana Castillo-Villanueva, Karina Martínez-Mayorga, Horacio Reyes-Vivas, Jesús Oria-Hernández.

```
Fibrobacter_succinogenes         ----------------------------------------------------MAVSYKELGLVNTKEMFAKAVKGGYAIPAFNFNTMEQMQAIVQAAVET
Elusimicrobium_minutum           ----------------------------------------------------MTVSYKELGFVNTKEMFANAMKEGYAVPAYNFNNMEQLQAIVTACVES
Hippea_maritima                  -----------------------------------------------------MVSYKELGFVNTKEMFKDAMEKGYAIPAYNFNNMEQLQAIITACTLT
Deferribacter_desulfuricans      ----------------------------------------------------MGISYKELGLVNTREMFKKAMEGKYAIPAYNFNNLEQLQAIIQACVET
Calditerrivibrio_nitroreducens   ----------------------------------------------------MAVSYKELGLVNTREMFKKAMEGKYAVPAYNFNNLEQLQAIIQACVET
Flexistipes_sinusarabici         ----------------------------------------------------MGVSYRELGLVNTRGMFAKAFKGGYAVPAYNFNNLEQLQAIIQACVET
Pelobacter_carbinolicus          --------------------------------------------------MAEETFYRELGLVNTRDMFEKAMSGGYAIPAYNFNNLEQLQAIVQACAET
Geobacter_lovleyi                -------------------------------------------------MEKKQVHYSELGLVNTKEMFAKAMAGKYAIPAYNFNNLEQLQAIVVACVET
Desulfurispirillum_indicum       ----------------------------------------------------MAVSFKDLGLCNTRDMFAGAMAGKYAIPAYNFNNMEQLQAIISGCAQS
Trichomonas_vaginalis            ----------------------------------------------------MAISYKSLGLCNSKDIFAKAVKGGYAIPGYNFSNLEQLQAIISASVKT
Entamoeba_dispar                 -------------------------------------------------MAAKTVNYKELGLCNHKEMFEHAIKGGFAVPGFNFNNLEQMQAIIQACTEA
Entamoeba_histolytica            -------------------------------------------------MAAKTVNYKELGLCNHKEMFEHAIKGGFAVPGFNFNNLEQMQAIIQACTEA
Leptotrichia_goodfellowii        ----------------------------------------------------MKYHFKDLGLSNTKEMFAKANKEGYSVPAFNFNNLEQLQGIIEACVEM
Leptotrichia_hofstadii           ----------------------------------------------------MKYHFKDLGLSNTKEMFAKANKEGYAVPAFNFNNMEQLQGIIEACVEE
Streptobacillus_moniliformis     ----------------------------------------------------MKYNYKDLGLVNTKEMFAKANREGYSVPAFNFNNLEQLQAIVEACVEM
Fusobacterium_mortiferum         ----------------------------------------------------MGYNYKDLGLSNTKEMFAKANANGYAVPAFNFNNMEMALAIVEACAEM
Fusobacterium_gonidiaformans     ----------------------------------------------------MGYTYRELGLSNTREMFAKANREGYAVPAFNFNNMEMALAIVEACAEM
Alkaliphilus_metalliredigens     -----------------------------------------------------------MPLVTSKELFENAYKGKYAVGAFNVNNMEIIQGIVDAAKEE
Veillonella_sp                   -----------------------------------------------------------MPLVTTTEMFKKAYEGGYAIGAFNVNNMEIVQGIVDAAKEE
Acetonema_longum                 -----------------------------------------------------------MPLVTSKEMFRKAYEGKYAIGAFNVNNMEIIQGIVDAAKEE
Thermosinus_carboxydivorans      -----------------------------------------------------------MPLVTSKEMFKKAYEGKYAIGAFNVNNMEIIQGIVDAAKEE
Pelosinus_fermentans             -----------------------------------------------------------MPLVTTKEMFKKAYDGQYAVGAFNVNNMEIIQGIVDAAKEE
Desulfosporosinus_sp             -----------------------------------------------------------MPLVTSTEMFKKVYG-KCAVGAFNVNNMEIIQGIVDAAKEE
Desulfosporosinus_acidiphilus    -----------------------------------------------------------MPLVTTTEMFKKVYG-KCAVGAFNVNNMEIIQGIVDAAKEE
Acidaminococcus_sp               -----------------------------------------------------------MPLVTTKDMFKKAYEGHYAVGAFNVNNMEIVQGIVDAAKEE
Acidaminococcus_intestini        -------------------------------------------------------------------MFKKAYEGHYAVGAFNVNNMEIVQGIVDAAKEE
Phascolarctobacterium_succinat   -------------------------------------------------------------------MFKKAYEGHYAVGAFNVNNMEIIQGIVAAAQQE
Caldicellulosiruptor_kristjans   -----------------------------------------------------------MPLVTTREMFKKAAEGKYAIGAFNVNNMEIIQGIVEAAKEE
Caldicellulosiruptor_lactoacet   -----------------------------------------------------------MPLVTTREMFKKAAEGKYAIGAFNVNNMEIIQGIVEAAKEE
Tepidanaerobacter_acetatoxydan   -----------------------------------------------------------MPLVTSKEMFKKAYEGKYAIGAFNVNNMEIIQGIVEAAQEE
Thermosediminibacter_oceani      -----------------------------------------------------------MPLVTSREMFKKAYEGGYAIGAFNVNNMEIIQGIVEAAREE
Caloramator_australicus          -----------------------------------------------------------MPLVTTKEMFKKAYEGGYAIGAFNINNMEIIQGIVEAAKEE
Eubacterium_saphenum             --------------------------------------------------MKAKRKENIMGLVTTEQMFKKSLCSDYAVGAFNVNNMEIIQGIVEAAKEE
Eubacterium_infirmum             -----------------------------------------------------------MALITTKEMFKKALVNDYAVGAFNVNNMEIIQGIVDAASIE
Acetobacterium_woodii            -----------------------------------------------------------MGLVTSTEMFKKAYEGGYAVGAFNVNNMEIIQGIVEAATEE
Flavonifractor_plautii           ------------------------------------------MKTDRIMWQINEGDEPSMPLVTTKEMFRKAYDGGYAIGAFNVNNMEIVQGITEAAREV
Oscillibacter_valericigenes      -----------------------------------------------------------MPLVTSTEMFKKAYDGGYAIGAFNVNNMEIVQGITEAARDL
Bryantella_formatexigens         ------------------------------------------------MVSKRKKEMDEMGLVTTKEMFAKAYNGGYAIGAFNVNNMEIVQGITEAAGEL
Stomatobaculum_longum            -----------------------------------------------------------MALVTSTEMFRKAYEGGYAIGAFNVNNMEIVQGITEAAAEL
Selenomonas_sputigena            -----------------------------------------------------------MPLIGTKEMFKKAHEGGYAIGAFNVNNMEIVQGITEAAAAL
Megasphaera_elsdenii             -----------------------------------------------------------MPLIGTTEMFKKAYEGHYAIGAFNINNMEIVQGVTQAAAEL
```

```
Mitsuokella_multacida              -------------------------------------------------------------------MFKKAYEGGFAIGAFNINNMEIVQGITEAAAEL
Selenomonas_infelix                -----------------------------------------------------------MPLVGTKEMFKKAYEGGYAIGAFNVNNMEIVQGITDAAGEL
Centipeda_periodontii              -----------------------------------------------------------MPLVGTKEMFKKAYEGGYAIGAFNVNNMEIVQGITDAAGEL
Megamonas_funiformis               -----------------------------------------------------------MPLVTTKEMFKKAYEGHYAIGAFNVNNMEIVQGITEAAKEL
Anaeroglobus_geminatus             ---------------------------------------------------------MTMPLVTTAEMFKKAYAGRYAVGAFNINNMEIVQAVTEAAGEL
Clostridium_thermocellum           -----------------------------------------------------------MPLVTSTEMFKKAYEGKYAIGAFNVNNMEIIQGITEAAKEV
Acetivibrio_cellulolyticus         -----------------------------------------------------------MPLVTSKEMFKKAYEGGYAIGAFNVNNMEIVQGITEAAKEV
Dictyoglomus_turgidum              -----------------------------------------------------------MPLVNPKELFQKCYG-KYAIGAFNVNNMEILQGVIEAAKEE
Dictyoglomus_thermophilum          -----------------------------------------------------------MPLVNPKELFQKCYG-KYAIGAFNVNNMEILQGVIEAAKEE
Thermotoga_lettingae               -----------------------------------------------------------MPLVSSKEMFKKAYG-KYAIGAFNVNNMEILQGVIEAAKEE
Thermotoga_thermarum               -----------------------------------------------------------MPLVSPKEMFKKAYG-KYAIGAFNVNNMEILQGVIEAAKEE
Caldisericum_exile                 -----------------------------------------------------------MPLVRTDELFKKAYG-KYAIGAFNVNNMEILQGVIDAAKEE
Bilophila_sp                       -----------------------------------------------------------MPLTSPRAMFERAYKEGYAIGAFNVNNMEIIQGIMEAGTEE
Bilophila_wadsworthia              -----------------------------------------------------------MPLTSPRAMFERAYKEGYAIGAFNVNNMEIIQGIMEAGTEE
Lawsonia_intracellularis           -----------------------------------------------------------MPLTGPKVMFEKAYKEGYAIGAFNVNNMEIIQGILMAATEE
Desulfohalobium_retbaense          -----------------------------------------------------------MPLVSPKEMFAKAYAGGYAIGAFNVNNMEIIQGIISAAETE
Desulfomicrobium_baculatum         -----------------------------------------------------------MPLTTPKEMFARGYAEGFAIGGFNVNNMEIIQGIMEAGNLE
Desulfovibrio_alaskensis           -----------------------------------------------------------MPLTGPKEMFARAYKEGYAIGAFNVNNMEIIQGIMQAGAEE
Desulfonatronospira_thiodismut     -----------------------------------------------------------MPLTSPKEMFDKAYQDGYAIGAFNVNNMEIIQGIMKAGEAE
Desulfovibrio_piger                ------------------------------------------------------MQEESMPLIGPKEMFAAAYAGRYAVGAFNVNNMEIVQGIMQAASEE
Dialister_succinatiphilus          -----------------------MEIRSPIQYNEIRYMHNPMKTMHSIHMNQFERKVITMPLVGTTEMFKKAYKGHYAIGAFNVNNMEIIQGIMEAAKEE
Dialister_micraerophilus           --------------------------------------------------------MFIMPIVGTKEMLKKAYIGKYAIGAFNVNNMEIVQGIMEAAHEK
Clostridium_botulinum              -----------------------------------------------------------MALVTTKEMFKKAYEGKYAIGAFNINNMEILQGVVNSAKAA
Peptostreptococcus_anaerobius      -----------------------------------------------------------MALVNTKEMFKKAYEGGYSIGAFNISDLEQLQAVLEACKSR
Arthromitus_sp                     -----------------------------------------------------------MPLVTTKEMFKKAYEGGYAIGAFNINNLESIQGIVEACKNK
Leptonema_illini                   -----------------------------------------------------------MPLQSPGLMFRKALNFRYAVGAFNVNNMEMLQGIVEAARAL
Anaerofustis_stercorihominis       -----------------------------------------------------------MALISSKEILQKAKEGKYAVGAFNVNNMEIIQGIVEAAEQE
Methanocella_arvoryzae             -----------------------------------------------------------MTFATLKEVLDTAKAGKYGVGAFNINNMEIAKAIGNAAKEE
Methanocella_conradii              -----------------------------------------------------------MIFSTLKEVLDRARDGKYAVGAFNINNMEIAKAIGGAAKEE
Staphylococcus_lugdunensis         -----------------------------------------------------------MPLVSMKEMLIDAKENGYAVGQYNLNNLEFTQAILEASQEE
Staphylococcus_aureus              -----------------------------------------------------------MPLVSMKEMLIDAKENGYAVGQYNINNLEFTQAILEASQEE
Listeria_grayi                     -----------------------------------------------------------MPIVSMTEMLKKALAGKYAVGQFNINNLEWTQAILKAAEAE
Brevibacillus_brevis               -----------------------------------------------------------MPLVPMTAFTEDVKKHKYAVGQFNLNNLEFTQAITEAAMEE
Kyrpidia_tusciae                   -----------------------------------------------------------MPLATLKEVLDDAFRNGYAVGQFNINNLEFTQAIMEAAEEE
Halanaerobium_praevalens           -----------------------------------------------------------MNLVPMADILQDAHQKTYAVGGFNINNMEFLQGIMMGAEEL
Halanaerobium_hydrogeniformans     -----------------------------------------------------------MNLVPMADILQDAHKRTYGVGGFNINNMEFLQGIIRGAEEL
Halothermothrix_orenii             -----------------------------------------------------------MNLVPMAEILKKANKEGYAVGGFNINNLEFLQGIIEAAEEM
Thermoanaerobacter_wiegelii        -----------------------------------------------------------MSLVNTKQMLQDAKKNKYAVGAFNIHNLETLKAVVKTAAEM
Helicobacter_pylori                ------------------------------------------------------------MLVKGNEILLKAHKEGYGVGAFNFVNFEMLNAIFEAGNEE
Helicobacter_acinonychis           ------------------------------------------------------------MLVRGNEILLKAHKEGYGVGAFNFVNFEMLNAIFEAGNEE
Thiovulum_sp                       ------------------------------------------------------------MLVSGAEILNKAHKEGYAVGAFNINNLEMLKAIFEAANEA
```

```
Fervidobacterium_nodosum          -----------------------------------------MKLRSLEIIILEKSKGGIFMYVNTKDILEKASKEYYAVPAFNINNMEFFHAILEGAIEK
Thermosipho_melanesiensis         ------------------------------------------------------------MYVNTKEILEDASKKYYAVPAFNINNLEFLMAILQGAVEK
Marinitoga_piezophila             -----------------------------------------------------------MPYVDTKVILENADKNGYGVPALNINNLEFLHYIIEAGVKM
Mesotoga_prima                    -----------------------------------------------------------MPYVNTKEILERANKEFYAVPALNINNLEFLQAIIDAGVEE
Aquifex_aeolicus                  -----------------------------------------------------------MPLVSGKVLFEKANEYNFAIGAFNFNNMEFLKAILDAAEEE
Truepera_radiovictrix             -----------------------------------------------------------MPLLTGLEILAAARAGGYGVGAFNTNNMEITQAILEAAEET
Thermus_thermophilus              ------------------------------------------------------------MLVTGLEILRKARAEGYGVGAFNTNNMEFTQAILEAAEEM
Meiothermus_silvanus              ------------------------------------------------------------MLATGMEILSKARKEGYGVGAFNVNNMEFVQAVLEAAEEV
Deinococcus_deserti               ------------------------------------------------------------MLVTGNDILVPARAGKYGVGSFNTNNMEITQAIIHTAERL
Meiothermus_ruber                 -----------------------------------------------------------MPLALGKDVLDKARREGYAVPSFNTNNLEITQAILETAEAL
Thermus_scotoductus               -----------------------------------------------------------MPLAIGKEVLDKARREGYAVPSFNTNNLEITQAILEVADEL
Giardia_lamblia                   -----------------------------------------------------------MPLCTLRQMLGEARKHKYGVGAFNVNNMEQIQGIMKAVVQL
Bacterium_phylotype_RsD17         -----------------------------------------------------------MALVSGKQILEEAKKKGYGVGAYNVNNMEQIQAIMAAAKET
Eikenella_corrodens               -----------------------------------------------------------MALVSMRQLLDHAAEHGYGLPAFNVNNLEQMRAIMEAADQV
Kingella_kingae                   -----------------------------------------------------------MALVSMRQLLDHAAEHSYGLPAFNVNNLEQMRAIMEAANQV
Neisseria_shayeganii              -----------------------------------------------------------MALVSLRQLLDHAAEHSYGLPAFNVNNLEQMRAIMEAADEV
Laribacter_hongkongensis          -----------------------------------------------------------MALVSMRQLLDHAAEFSYGLPAFNVNNLEQMRAIMEAADKC
Acinetobacter_baumannii           -----------------------------------------------------------MALISMRQLLDHAAEHNYGVPAFNVNNLEQMRAIMLAADAT
Acinetobacter_sp                  -----------------------------------------------------------MALISLRQLLDHAAEHNYGVPAFNVNNLEQMRAIMLAADET
Marinomonas_mediterranea          -----------------------------------------------------------MPLISMRQLLDHAAEHSYGLPAFNVNNMEQVKAIMEAADEV
Oceanospirillum_sp                -----------------------------------------------------------MALISMRQLLDHAAENDYGIPAFNVNNLEQMRAIMEAADKT
Marinobacterium_stanieri          -----------------------------------------------------------MALISMRQLLDHAAEYGYGIPAFNVNNLEQMRAIMEAAAKT
Oceanobacter_sp                   -----------------------------------------------------------MALISMRQMLDHAAEYGYGVPAFNVNNLEQMRAIMMAADKT
Colwellia_psychrerythraea         -----------------------------------------------------------MALISMRQMLDHAAEFEYGIPAFNVNNLEQVRAIMLAASDT
Hahella_chejuensis                -----------------------------------------------------------MALITMRQLLDHAAEHGYGVPAFNVNNLEQMRAIMEAADQT
Alishewanella_aestuarii           -----------------------------------------------------------MALISLRQMLDHAAEFGYGVPAFNVNNLEQMRAIMLAADKT
Alishewanella_jeotgali            -----------------------------------------------------------MALISLRQMLDHAAEFGYGVPAFNVNNLEQMRAIMLAADKT
Rheinheimera_nanhaiensis          -----------------------------------------------------------MALISLRQMLDHAAEFGYGIPAFNVNNLEQMRAIMLAADKT
Congregibacter_litoralis          -----------------------------------------------------------MALISLRQLLDHAAEHDYGVPAFNVNNLEQTRAIMEAADAT
Pseudoalteromonas_marina          -----------------------------------------------------------MALISMRQLLDHAAEHGYGVPAFNVNNQEQMRAIMEAADKT
Alteromonadales_bacterium         -----------------------------------------------------------MALISMRQLLDHAAEHGYGVPAFNVNNQEQMRAIMEAADKT
Idiomarina_baltica                -----------------------------------------------------------MAIVSLRQVLDHAAENGYGVPAFNVNNLEQMRAIMQAADKT
Shewanella_baltica                -----------------------------------------------------------MALISLRQLLDHAAEHGYGVPAFNVNNLEQMRAIMQAAEAT
Shewanella_violacea               -----------------------------------------------------------MALISLRQMLDHAAEHDYGVPAFNVNNLEQMRAIMQAAEAT
Ferrimonas_balearica              -----------------------------------------------------------MALISLRQMLDHAAEHNYGVPAFNVNNLEQMRAIMQAAEET
Pseudomonas_mendocina             -----------------------------------------------------------MALISMRQMLDHAAEFGYGVPAFNVNNLEQMRAIMEAADKT
Pseudomonas_fulva                 -----------------------------------------------------------MALISMRQMLDHAAEFGYGVPAFNVNNLEQMRAIMEAADKT
Azotobacter_vinelandii            -----------------------------------------------------------MALISMRQLLDHAAEFGYGVPAFNVNNLEQMRAIMEAADKT
Halomonas_sp                      -----------------------------------------------------------MALISMRQMLDHAAERGYGIPAFNVNNLEQMRAIMEAADAT
Halomonas_boliviensis             -----------------------------------------------------------MALISMRQMLDHAAEYGYGIPAFNVNNLEQMRAIMEAADAT
Chromohalobacter_salexigens       -----------------------------------------------------------MALISLRQLLDHAAEHAYGVPAFNVNNLEQMRAIMEAADRT
```

```
Marinobacter_algicola            -----------------------------------------------------------MALISMRQLLDHAAEHGYGVPAYNVNNLEQMRAIMEAADKT
Marinobacter_aquaeolei           -----------------------------------------------------------MALISLRQLLDHAAEHGYGVPAFNVNNLEQMRAIMEAADKT
Thiocystis_violascens            -----------------------------------------------------------MALISLRQMLDHAAEHGYGVPAYNVNNLEQMRAIMEAADET
Thiocapsa_marina                 -----------------------------------------------------------MALISLRQLLDHAAEHQYGVPAYNVNNLEQMRAIMEAADET
Allochromatium_vinosum           -----------------------------------------------------------MALISMRQMLDHAAEHGYGVPAFNVNNLEQMRAIMEAADET
Thiorhodococcus_drewsii          -----------------------------------------------------------MALISMRQLLDHAAEHGYGVPAFNVNNLEQMRAIMEAADQT
Marichromatium_purpuratum        -----------------------------------------------------------MALISMRQLLDHAAERGYGVPAFNVNNLEQMRAIMEAADET
Thiomonas_intermedia             -----------------------------------------------------------MALISLRQLLDHAAEHSYGVPAFNVNNLEQVRAIMEAADET
Francisella_novicida             -----------------------------------------------------------MALVSLRQLLDHAAEHGYGLPAFNVNNLEQVRAVMEAADKV
Francisella_noatunensis          -----------------------------------------------------------MALVSLRQLLDHAAEHGYGLPAFNVNNLEQVRAVMEAADKV
Kangiella_koreensis              -----------------------------------------------------------MALITLRQLLDHAAEHNYGIPAFNVNNLEQMRAVMQAADET
Coxiella_burnetii                -----------------------------------------------------------MALITLRQLLDHAAEHGYGVPAFNVNNLEQVRAIMEAADET
Thiothrix_nivea                  -----------------------------------------------------------MALISMRQLLDHAAEHGYGMPAFNVNNMEQVHAIMQAADAT
Thiobacillus_denitrificans       -----------------------------------------------------------MALISLRQLLDHAAENGYGLPAFNVNNMEQVKAIMEAAAAV
Thioalkalimicrobium_cyclicum     -----------------------------------------------------------MAMITLRELMDYAAEHSFGMPAFNVNNMEQVRAIMRAADAV
Halothiobacillus_neapolitanus    -----------------------------------------------------------MALISLRQLLDHAAEHSYGMPAFNVNNMEQIHAIMQAADAV
Nitrosococcus_watsonii           -----------------------------------------------------------MALITLRQLLDYAAEHSFGIPAFNVNNMEQVHSIMQAADAV
Halorhodospira_halophila         -----------------------------------------------------------MAMITLRQLLDHAAEHGYGMPAFNANNMEQLHAIMEAAKEC
Alkalilimnicola_ehrlichii        -----------------------------------------------------------MALITLRQLLDHAAEHGYGMPAFNANNMEQFHAIMEAARET
Rubrivivax_benzoatilyticus       -----------------------------------------------------------MPLIALRPLLDHAAEHDYGVPAFNVNNMEQIQAILQAAQAC
Rubrivivax_gelatinosus           -----------------------------------------------------------MPLIALRPLLDHAAEHDYGVPAFNVNNMEQIQAILQAAQAC
Hydrocarboniphaga_effusa         -----------------------------------------------------------MALISLRQLLDHAAEHDYGLPAFNVNNLEQIQAIMQAADAC
Dechloromonas_aromatica          -----------------------------------------------------------MALISLRQLLDHAAEHSYGLPAFNVNNMEQIQAIMQAAEAC
Oxalobacteraceae_bacterium       -----------------------------------------------------------MALISLRQLLDHAAQFEYGVPAFNVNNLEQIQAIMQAADQT
Leptothrix_cholodnii             -----------------------------------------------------------MALISLRQLLDHAAEHDYGVPAFNVNNMEQVQSIMEAAQAT
Methylovorus_glucosetrophus      -----------------------------------------------------------MALITLRQALDHAAEYGYALPAFNVNNLEQIQAVMQAADAV
Methylobacillus_flagellatus      -----------------------------------------------------------MALISLRQLLDHAAEHDYGVPAFNVNNLEQVQAIMRAADSC
Methylotenera_versatilis         -----------------------------------------------------------MALVSLRQLLDHAAEHDYGLAAFNVNNMEQVHAIMQAADEV
Nitrosospira_multiformis         -----------------------------------------------------------MALVSLRQLLDHAAENGYGLPAFNVNNLEQIHAIMQAADEC
Gallionella_capsiferriformans    -----------------------------------------------------------MALVSLRQLLDHAAEHSYGLPAFNVNNLEQVKAIMEAADEC
Methylomirabilis_oxyfera         -----------------------------------------------------------MALVSMRQLLDHAAEYGYGVPAFNVNNMEQIQAIMEAAHET
Acidithiobacillus_caldus         -----------------------------------------------------------MALVSMRQLLDHAAEHGYGIPAFNVNNLEQVRAIMEAAAAT
Magnetospirillum_magneticum      -----------------------------------------------------------MSLVSMRQLLDHAAENSYGIPAFNVNNMEQVKAIMEAASAC
Magnetospirillum_magnetotactic   ----------------------------------------------------------------MRQLLDHAAENGYGIPAFNVNNMEQVKAIMEAASAC
Phaeospirillum_molischianum      -----------------------------------------------------------MSLVSLRQLLDHAAEHGYGIPAFNVNNMEQVKAIMEAAAAT
Aromatoleum_aromaticum           -----------------------------------------------------------MPLVSMRQLLDHAAENGYGLPAFNVNNLEQVQAIMEAAAEA
Thauera_sp                       -----------------------------------------------------------MPLVSMRQLLDHAAEHSYGLPAFNVNNLEQVQAIMEAAAEC
Azoarcus_sp                      -----------------------------------------------------------MPLVSMRQLLDHAAENSYGLPAFNVNNLEQVQAIMEAAAET
Dechlorosoma_suillum             -----------------------------------------------------------MPIVSMRQLLDHAAENGYGLPAFNVNNMEQVWAIMEAANEL
Accumulibacter_phosphatis        -----------------------------------------------------------MPIVSMRQLLDHAAENGYGLPAFNVNNMEQVWAIMEAAAEV
Herbaspirillum_sp                -----------------------------------------------------------MPLVSMRQLLDHAAENGYGLPAFNVNNLEQVTAIMQAADEV
```

```
Herbaspirillum_seropedicae      -----------------------------------------------------------MPLVSMRQLLDHAAENGYGLPAFNVNNLEQVTAIMEAANEV
Collimonas_fungivorans          -----------------------------------------------------------MPLVSMRQLLDHAAENSYGLPAFNVNNLEQVTAIMQAADEA
Herminiimonas_arsenicoxydans    -----------------------------------------------------------MPLVSMRQLLDHAAENGYGLPAFNVNNLEQVTAIMEAADEV
Polynucleobacter_necessarius    -----------------------------------------------------------MALVSLRQLLDHAAENGYGLPAFNVNNLEQVTAIMEAANEA
Janthinobacterium_sp            -----------------------------------------------------------MSLVSMRQLLDHAAENGYGIPAFNVNNLEQVQAIMAAADAL
Burkholderia_cenocepacia        -----------------------------------------------------------MPLVSMRQLLDHAAEHGYGLPAFNVNNLEQVQAIMAAADQV
Oxalobacter_formigenes          -----------------------------------------------------------MPIVSMRQLLDHAAENGYGLPAFNVNNLEQVQAIMEAAAEV
Ralstonia_eutropha              -----------------------------------------------------------MPLVSMRQLLDHAAENGYGLPAFNVNNLEQVQAIMQAADEV
Cupriavidus_taiwanensis         -----------------------------------------------------------MPLVSMRQLLDHAAENSYGLPAFNVNNLEQVQAIMQAADEV
Cupriavidus_necator             -----------------------------------------------------------MPLVSMRQLLDHAAENSYGLPAFNVNNLEQVQAIMQAADEV
Acidovorax_citrulli             -----------------------------------------------------------MPLVSMREMLDHAAENRYGIPAFNVNNLEQVQAVMSAADEV
Comamonas_testosteroni          -----------------------------------------------------------MPLISMREMLDHAAENGYGIPAFNVNNLEQVQAVMSAADEV
Acidovorax_radicis              -----------------------------------------------------------MPLVSMRELLDHAAANSYGIPAFNVNNLEQVQAVMAAADEL
Limnobacter_sp                  -----------------------------------------------------------MPLVSMRQLLDHAAENGYGIPAFNVNNLEQVQAVMAAADEV
Hylemonella_gracilis            -----------------------------------------------------------MPLVSMRELLDHAAENGYGIPAFNVNNLEQVQAVMAAADEV
Methyloversatilis_universalis   -----------------------------------------------------------MPLVSMRQLLDHAAENGYGLPAFNVNNLEQVQAVMSAADEV
Alicycliphilus_denitrificans    -----------------------------------------------------------MPLVSMRELLDHAADSGYGIPAFNVNNLEQVQAVMAAADEV
Delftia_sp                      -----------------------------------------------------------MPLVSMRELLDHAAENGYGLPAFNVNNLEQVQAVMSAADEV
Delftia_acidovorans             -------------------------------------------MRQGHPPLNNLIRTLHMPLVSMRELLDHAAENGYGLPAFNVNNLEQVQAVMSAADEV
Ralstonia_pickettii             -----------------------------------------------------------MPLVSMRQLLDHAAENGYGLPAFNVNNLEQVQAVMEAAKEA
Variovorax_paradoxus            -----------------------------------------------------------MALVSMRELLDHAAANGYGIPAFNVNNLEQVQAVMEAAKET
Hydrogenophaga_sp               -----------------------------------------------------------MALVSMRELLDHAAANTYGIPAFNVNNLEQVQAVMAAADEV
Ramlibacter_tataouinensis       -----------------------------------------------------------MPLVSMRELLDHAAENGYGIPAFNVNNLEQVQAVMEAAKET
Polaromonas_naphthalenivorans   -----------------------------------------------------------MALVSMRELLDHAAANGYGIPAFNVNNLEQVQAVMAAADEV
Methylibium_petroleiphilum      -----------------------------------------------------------MPLVSMRQLLDHAAEQGYGIPAFNVNNLEQVQAVMSAAHEV
Achromobacter_xylosoxidans      -----------------------------------------------------------MALVSMRQLLDHAAEHGYGIPAFNVNNLEQVQAIMEAAAET
Achromobacter_arsenitoxydans    -----------------------------------------------------------MALVSMRQLLDHAAEHGYGIPAFNVNNLEQVQAIMEAAAET
Bordetella_petrii               -----------------------------------------------------------MALVSMRQLLDHAAEHGYGIPAFNVNNLEQVQAIMEAAAET
Bordetella_pertussis            -----------------------------------------------------------MALVSMRQLLDHAAEHGYGIPAFNVNNLEQVQAIMEAAAET
Advenella_kashmirensis          -----------------------------------------------------------MALVSMRQLLDHAAENGYGIPAFNVNNLEQVQAIMEAAKET
Pusillimonas_sp                 -----------------------------------------------------------MALVSMRQLLDHAAENGYGIPAFNVNNLEQVQAIMEAAAET
Alcaligenes_faecalis            -----------------------------------------------------------MALVSMRQLLDHAAEHGYGIPAFNVNNLEQVQAIMEAASET
Taylorella_equigenitalis        -----------------------------------------------------------MALVSLRQLLDHAAENGYGIPAFNVNNLEQVQAIMEAAKET
Parasutterella_excrementihomin  -----------------------------------------------------------MALVSLRQLLDHAAENNYGIPAFNVNNLEQVQAIMQAADEV
Burkholderiales_bacterium       -----------------------------------------------------------MALVSLRQLLDHAAENNYGIPAFNVNNLEQVQAIMQAADEV
Sutterella_wadsworthensis       -----------------------------------------------------------MALVSLRQLLDHAAENGYGIPAFNVNNLEQVQAIMAAASSV
Xanthobacter_autotrophicus      -----------------------------------------------------------MALVSMRQLLDHAAELSYGLPAFNVNNMEQVKAIMDAARAT
Magnetococcus_marinus           -----------------------------------------------------------MPLVSMRQLLDHAAENSYGLPAFNVNNMEQVRAIMRAADET
Nostoc_punctiforme              -----------------------------------------------------------MALVPLRLLLDHAAENGYGIPAFNVNNLEQIQAILKAAVET
Anabaena_variabilis             -----------------------------------------------------------MALVPLRLLLDHAAENGYGIPAFNVNNLEQIQAILKAAAET
Microcystis_aeruginosa          -----------------------------------------------------------MALVPMRLLLDHAAENGYGIPAFNVNNMEQIQAIMQAAAAT
```

```
Lyngbya_sp                        -----------------------------------------------------------MALVPMRLLLDHAAENNYGIPAYNVNNMEQIIAIMQAADET
Arthrospira_platensis             -----------------------------------------------------------MAIVPMRLLLDHAAENDYGIPAYNVNNMEQIQSIMQAAHET
Oscillatoria_sp                   -----------------------------------------------------------MAIVPMRLLLDHAAENGYGIPAYNVNNMEQIQAIMRAADET
Cyanothece_sp                     -----------------------------------------------------------MALVPMRLLLDHAAENGYGIPAFNVNNLEQILSIMKAAHET
Synechococcus_elongatus           -----------------------------------------------------------MALVPLRLMLDHAAENEYGIPAFNVNNLEQVQSILQAADET
Prochlorococcus_marinus           -----------------------------------------------------------MALVPLRLLLDHAAENGYGIPAFNVNNLEQVQAIMEAASET
Paulinella_chromatophora          -----------------------------------------------------------MALVPLRLLLDHAAENGYGIPAFNVNNLEQVQSIMEAAHET
Synechococcus_sp                  -----------------------------------------------------------MALVPMRILLDHAAEHDYGIPAFNVNNMEQIQAIMQAAHET
Gloeobacter_violaceus             -----------------------------------------------------------MALVPLRVLLDHAAENNYGIPAFNVNNLEQIKAIMDAAREV
Sinorhizobium_meliloti            -----------------------------------------------------------MALITLRQLLDDAAENDYALPAFNVNNLEYIQAVMRAADAT
Stappia_aggregata                 -----------------------------------------------------------MARITLRQLLDHAAEHDYGVPAFNINNMEQALAIMAAADQT
Labrenzia_alexandrii              -----------------------------------------------------------MARITLRQLLDHAAENDYGVPAFNINNMEQALAIMAAADQT
Polymorphum_gilvum                --------------------------------------MVRDPFHPARDAPTAFKWRRNMARITLRQLLDHAAEHDYGVPAFNINNMEQALAIMEAADAT
Bradyrhizobium_sp                 -----------------------------------------------------------MARITLRQLLDHAAENDYGVPAFNINNMEQALAIMDAANSV
Agrobacterium_radiobacter         -----------------------------------------------------------MARITLRQLLDHAAENGYGVPAFNINNMEQALAIMEAADAA
Rhizobium_sp                      -----------------------------------------------------------MARITLRQLLDHAAENGYGVPAFNINNMEQALAIMEAADAA
Brucella_melitensis               -----------------------------------------------------------MARITLRQLLDHAAEKGYGVPAFNINNMEQALAIMEAANAV
Brucella_suis                     -----------------------------------------------------------MARITLRQLLDHAAEKGYGVPAFNINNMEQALAIMEAANAV
Ochrobactrum_anthropi             -----------------------------------------------------------MARITLRQLLDHAAEKGYGVPAFNINNMEQALAIMEAADAA
Sinorhizobium_fredii              -----------------------------------------------------------MARITLRQLLDDAAEHGYGVPAFNINNMEQALAIMEAADGT
Mesorhizobium_amorphae            -----------------------------------------------------------MARITLRQLLDHAAEHGYGVPAFNMNNMEQGLAIMEAAEET
Mesorhizobium_loti                -----------------------------------------------------------MARITLRQLLDHAAEYGYGVPAFNMNNMEQGLAIMEAAEET
Methylobacterium_nodulans         -----------------------------------------------------------MARITLRQLLDHAAEHGYGVPAFNINNMEQGLAIMTAADAC
Methylobacterium_radiotolerans    -----------------------------------------------------------MARITLRQLLDHAAEYEYGVPAFNLNNMEQGLAIMAAADAT
Phenylobacterium_zucineum         -----------------------------------------------------------MARITLRQLLDHAAEHGYGVPAFNINNMEQALAIMEAAEAT
Caulobacter_sp                    -----------------------------------------------------------MARITLRQLLDHAAEHDYALPAFNINNMEQGLAIMEAAEAV
Caulobacter_crescentus            -----------------------------------------------------------MARITLRQLLDHAAEHEYGLPAFNINNMEQGLAIMEAADAV
Paracoccus_denitrificans          -----------------------------------------------------------MALITLRQLLDHAAEHGYGVPAFNINNMEQGLAIVKAAAEV
Paracoccus_sp                     -----------------------------------------------------------MALITLRQLLDHAAEHGYGVPAFNINNMEQGLAIVKAAAEV
Pelagibaca_bermudensis            -----------------------------------------------------------MALITLRQLLDHAAENGYGVPAFNINNMEQGLAIMEAAEAC
Citreicella_sp                    -----------------------------------------------------------MALITLRQLLDHAAEHGYGVPAFNINNMEQGLAIMEAAEGC
Rhodopseudomonas_palustris        -----------------------------------------------------------MARITLRQLLDHAAEHGYGVPAFNINNMEQGLAIMEAAAAV
Starkeya_novella                  -----------------------------------------------------------MARITLRQLLDHAAEHGYGVPAFNINNMEQGIAIMDAAQAV
Azospirillum_amazonens            -----------------------------------------------------------MPRITLRQLLDHAAERDYGVPAFNINNMEQGLAVMEAAAQV
Rhodobacter_sphaeroides           -----------------------------------------------------------MALITLRQLLDHAAEQGYGVPAFNINNMEQGLAIMEAARAC
Aurantimonas_manganoxydans        -----------------------------------------------------------MAKITLRQLLDHAAENGYGVPAFNINNMEQGLAIMEAAAKS
Octadecabacter_arcticus           -----------------------------------------------------------MALVSLRQLLDHAAEYNYAIPAFNVNNMEQMLAIMSAGHST
Sulfitobacter_sp                  -----------------------------------------------------------MPLISLRQLLDHAAENGYGLPAFNVNNMEQMLAIMQAADKA
Rhodospirillum_photometricum      ---------------------------------MIPRPVSWTKPLGASAPPRFIQGDVPMALISLRQLLDHAAEYEYGMPAFNVNNMEQVLSIMNAAKKT
Pirellula_staleyi                 -----------------------------------------------------------MPLVSLRVVLDHAAENNYGVAAFNVNNMEQIQSIMEAAAET
Blastopirellula_marina            -----------------------------------------------------------MPLITLRVLLDHAAENNYGVAAFNVNNMEQIQSIMEAAAET
```

```
Rhodopirellula_baltica              -----------------------------------------------------------MPLVPLRVVLDHAAENDYGVAAFNVNNMEQIQAIMEAADET
Koribacter_versatilis               -----------------------------------------------------------MPLCSMRQVLDEAAKGNYGVGAFNVNNMEQIQAIMEAARET
Solibacter_usitatus                 -----------------------------------------------------------MPLVSMRQLLDEAAKGGYGVGAFNVNNMEQIQSIMEAARET
Anaerolinea_thermophila             ------------------------------------------------------------MIVTTKKLFEAAYG-KYAIGAYNINNLEQCMGLFRGNLES
Diplosphaera_colitermitum           ----------------------------MNKIRAEKTLAGPAPAFQPRPFGSFTRYYNTTMIVTTAQLFKHAYG-KYAVGAYNINNAEQTMGLFKGAIAS
CONSERVATION                                                                                          *    *

                                             110       120       130       140       150       160       170       180       190       200
                                    ....|....|....|....|....|....|....|....|....|....|....|....|....|....|....|....|....|....|....|....|
Chlorobium_phaeovibrioides          KSPVILQVSKGARSYAN-------ETLLRY--LAQGAVAYAEEL-----GSPIP-IVLHLDHGDTFELCKDCIESGFSS-VMIDG-------SHLPYDEN
Pelodictyon_phaeoclathratiform      KSPVILQVSKGARSYAN-------ETLLRY--LAQGAVAYAAEL-----GSPIP-IVLHLDHGDSFELCKDCIDSGFSS-VMIDG-------SHLSYEDN
Chlorobium_chlorochromatii          QSPVILQVSKGARSYAN-------ETLLRY--LAQGAVAYAEEL-----GSSIP-IVLHLDHGDSLELCKDCIDSGFSS-VMIDG-------SHLSYEEN
Prosthecochloris_aestuarii          NSPVILQVSKGARNYAN-------QTLLRN--LARGAVEYAAEL-----GNPVP-IVLHLDHGDSFELCKDCIQTGFSS-VMIDG-------SHLSYEDN
Chlorobaculum_parvum                SSPVILQVSKGARNYAN-------QTLLRH--LAAGAVEYAAEL-----GCAVP-IVLHLDHGDSFELCKDCIETGFSS-VMIDG-------SHLSYDEN
Victivallis_vadensis                QSPVILQVSKGAREYAN-------QTLLRY--MAQGAVQYAKEISPD--GKGIP-IVLHLDHGPDFETCKSCIDNGFSS-VMIDG-------SHLPYEEN
Brachyspira_murdochii               KSPVIIQVSSGARKYAN-------QTLLRY--MAQGAVEYAKEL-----GVNVP-IVLHLDHGDSLELCKSCIEYGFSS-VMIDG-------SHYDYNKN
Brachyspira_pilosicoli              KSPVIIQVSSGARKYAN-------QTLLRY--MAQGAVEYAKEL-----GVNVP-IVLHLDHGDSLELCKSCIEYGFSS-VMIDG-------SHHDYNTN
Alistipes_indistinctus              KSPVILQVSKGARQYAN-------QTLLRY--MAEGAVEYAKEL-----GLKHPEIVLHLDHGDSFETCKSCIDMGFSS-VMIDG-------SHLPYDEN
Tannerella_sp                       NSPVILQVSKGARQYAN-------QTLLRY--MAEGAVEYAKEL-----GCKNPQIVLHLDHGDSFELCKSCIDMGFSS-VMIDG-------SHLPYDEN
Tannerella_forsythia                KSPVILQVSSGARKYAN-------QTILRY--MAEGAVAYAKEL-----GCPNPQIVLHLDHGDTFELCKSCIDMGFSS-VMIDG-------SHLPYDEN
Bacteroides_intestinalis            KSPVILQVSKGARQYAN-------QTLLRY--MAEGAVEYAKEL-----GCKHPEIVLHLDHGDTFETCKSCIDMGFSS-VMIDG-------SHLPYEEN
Bacteroides_uniformis               KSPVILQVSKGARQYAN-------ATLLRY--MAQGAVEYAKEL-----GCKHPEIVLHLDHGDTFETCKSCIDSGFSS-VMIDG-------SHLPYEEN
Parabacteroides_merdae              KSPVILQVSKGARQYAN-------ATLLRY--MAQGAVEYAKEL-----GCPNPQIVLHLDHGDTFETCKSCIDSGFSS-VMIDG-------SHLPYEEN
Parabacteroides_sp                  KSPVILQVSKGARQYAN-------ATLLRY--MAQGAVEYAKEL-----GCPNPQIVLHLDHGDTFETCKSCIDSGFSS-VMIDG-------SHLPYEEN
Capnocytophaga_sp                   KSPVILQVSKGARQYAN-------ATLLRY--MAQGAVEYAKEL-----GCANPQIVLHLDHGDTFETCKSCIDSGFSS-VMIDG-------SHLPYDEN
Paraprevotella_clara                KSPVILQVSKGARQYAN-------ATLLRY--MAQGAVEYAKEL-----GCANPQIVLHLDHGDTFETCKSCIDSGFSS-VMIDG-------SHLPYDEN
Paraprevotella_xylaniphila          KSPVILQVSKGARQYAN-------ATLLRY--MAQGAVEYAKEL-----GCANPQIVLHLDHGDSFETCKSCIDSGFSS-VMIDG-------SHLPYDEN
Bacteroides_salanitronis            KSPVILQVSKGARNYAN-------QTLLRY--MAEGAVAYAKEL-----GCEHPEIVLHLDHGDSFELCKSCVDLGFSS-VMIDG-------SHLPYEEN
Dysgonomonas_gadei                  KSPVIVQASKGARQYAN-------PILLRY--MLQGAVEYAKSL-----GWEKPQIVLHLDHGDTFETCKDCIDSGFSS-VMIDG-------SHLPYDEN
Dysgonomonas_mossii                 KSPVIVQASKGARQYAN-------PILLRY--MLQGAVEYAKSL-----GWEKPQIVLHLDHGDTFETCKDCIDSGFSS-VMIDG-------SHLPYEEN
Paludibacter_propionicigenes        KSPVILQVSSGARKYAN-------QTLLRY--MAQGAVEYAKEL-----GLNIP-VVLHLDHGDTFELCKDCIDSGFSS-VMIDG-------SHHSYEDN
Alistipes_sp                        SSPVILQVSSGARKYAN-------QTLLRY--MAQGAVEYAKEL-----GKNIP-IVLHLDHGNSFELCKSCIDMGFSS-VMIDG-------SHLPYEEN
Odoribacter_laneus                  KSPVILQVSSGARKYAN-------QTILRY--MAQGAVEYAKEL-----GLNIP-IVLHLDHGDSFELCKSCIDMGFSS-VMIDG-------SHLSYDEN
Porphyromonas_endodontalis          NSPVILQVSSGARKYAN-------QTLLRY--MAQGAVEYAKEL-----GQPNPQICLHLDHGDTFELCKSCIELGFSS-VMIDG-------SALPYDEN
Candidatus_Azobacteroides           NSPVILQVSSGARKYAN-------QTILRY--LAQGAVGYAKEL-----GKNIP-IVLHLDHGDTFELCKSCIDFGFSS-VMIDG-------SHHSYNEN
Sphaerochaeta_pleomorpha            KSPVILQVSKGARDYAN-------MNILRN--MARGATEYAKEL-----GCEIP-IVLHLDHGDSFETCKECIDNGFSS-VMIDG-------SHFPYDEN
Sphaerochaeta_coccoides             RSPVILQVSKGARDYAN-------INLLRN--MARGATEYAKEL-----GYEIP-IVLHLDHGDSFETCKDCIDNGFSS-VMIDG-------SHLPYEEN
Spirochaeta_caldaria                KSPVILQVSSGARKYAN-------QTLLRH--MARGAVEYAHEL-----GYDIP-IVLHLDHGDSFELCKDCIESGFSS-VMIDG-------SHLPYDEN
Treponema_saccharophilum            KSPVILQVSKGARQYAN-------GILLRN--MAKGAVEYAHEL-----GCDIP-IVLHLDHGPNFEVAKDCIDNGFSS-VMIDG-------SALPYDEN
Treponema_sp                        KSPVILQVSKGARAYAN-------ANILRN--MARGAVEYAHEL-----GCDIP-IALHLDHGPNFEVAKDCIDNGFSS-VMIDG-------SALPYDEN
Melioribacter_roseus                KSPVILQVSSGARKYAN-------QTLLKN--LAKGAVEYAEEL-----GYKIP-IVLHLDHGDTFELCKSCIEMGFSS-VMIDG-------SHHPYEKN
```

```
Spirochaeta_thermophila            RSPVILQVSKGAREYAN-------PTLLRH--MARGAVEYAREL-----GYEIP-IVLHLDHGDSYELCKSCVDMGFSS-VMIDG-------SHLPYEEN
Prevotella_multisaccharivorax      KSPVILQVSKGARAYAN-------PILLRY--MAQGAVEFAKSL-----GCHHPEIALHLDHGDTFELVKDCVDLGFSS-VMIDG-------SSKPYEEN
Prevotella_disiens                 KSPVILQVSKGARKYAN-------QTLLRY--LAEGAVEYAKEL-----GCNHPEIVLHLDHGDSFETCKSCVDFGFSS-VMIDG-------SSLPYEEN
Fibrobacter_succinogenes           KSPVIMQVSKGARNYAN-------GTILRY--MAQGAVEYAKEL-----GCANPQIVLHLDHGDSFELCKDCIDNGFSS-VMIDG-------SALPYEDN
Elusimicrobium_minutum             GSPVIVQVSSGARKYAN-------ATMLRW--MARGAVEMMKDIG-----KPVP-IALHLDHGDSFELCKDCIDSGFSS-VMIDG-------SHLPYEEN
Hippea_maritima                    QSPVILQVSKGARKYAN-------QTLLRW--MAQGAVELMKEIASESGLKEIP-IALHLDHGDSFELCKSCIETGFSS-VMIDG-------SHLPYEEN
Deferribacter_desulfuricans        RSPVILQVSKGARQYAN-------ATMLRY--MAMGAVEYAKELG-----YEIP-IALHLDHGDSFELCKACVDNGFSS-VMIDG-------SHLPFEEN
Calditerrivibrio_nitroreducens     KSPVILQVSKGAREYAN-------ATILRY--LALGATQLAEEMG-----HKIP-IALHLDHGDSFEICKSCVDYGFSS-VMIDG-------SHLPFEEN
Flexistipes_sinusarabici           KSPVILQVSKGAREYAN-------ATMLRY--MAMGATELAKELG-----GDIP-IALHLDHGGDFDICKSCVDNGFSS-VMIDG-------SHLSFEKN
Pelobacter_carbinolicus            ASPVIIQVSKGARNYAN-------ATMLRY--MAMGAVRMAREMG-----SEIP-ISLHLDHGDSFELCQSCIESGFSS-VMIDG-------SHLPYDEN
Geobacter_lovleyi                  NSPVIIQVSKGARSYAN-------ETMLRY--MAMGAVQMARELG-----STIP-ICLHLDHGDSFELCKSCVDSGFSS-VMIDG-------SHLPYDEN
Desulfurispirillum_indicum         RSPVILQVSKGARNYAN-------GTLLRY--MAMGAVELAHQT------APIP-IALHLDHGDSFELCKDCVDSGFSS-VMIDG-------SHLSFQEN
Trichomonas_vaginalis              ESPVILQVSAGARKYAN-------QTMLRY--MAQAASEYAKEIHP---EHKLIPIVLHLDHGDSFELCKNCIDLGFSS-VMIDG-------SHLPYDEN
Entamoeba_dispar                   KSPVILQVSKGAREYAN-------ATLLRN--LAKGAVEYSKEIDP--EHKGIP-ISLNLDHGANFQICKECVDNGFSN-VMIDG-------SALPYEEN
Entamoeba_histolytica              KSPVILQVSKGAREYAN-------ATLLRN--LAKGAVEYSKEIDP--EHKGVP-ISLNLDHGANFQICKECVDNGFSN-VMIDG-------SALPYEEN
Leptotrichia_goodfellowii          GSPVILQVSTGARSYIG-------KEMLPW--LAKAATAYVEAS-----GSDIP-VALHLDHGPNFEEAKDCIEYGFSS-VMIDA-------SHHPYDEN
Leptotrichia_hofstadii             GSPVILQVSTGARKYIG-------KEMLPW--LAKAATAYVEAS-----GSDIP-VALHLDHGPNFAEAKDCIEYGFSS-VMYDG-------SHHPYDEN
Streptobacillus_moniliformis       GSPVILQVSTGARKYIG-------KEMLPF--LAKAATAYVKAT-----GSDIP-IALHLDHGPSFEACKDCIEYGFSS-VMIDA-------SHHSFDEN
Fusobacterium_mortiferum           GSPVILQCSKGALSYMG-------PEVTPL--LAKAAVDRARSM-----GSDIP-VALHLDHGPDLATVKTCIEAGFSS-VMIDG-------SHYDFAKN
Fusobacterium_gonidiaformans       GSPVILQCSAGAIKYMG-------YDVAPL--MAKAAVDRARNM-----GSDIP-VALHLDHGADLETVKKCIAAGFSS-VMIDA-------SHYDYEEN
Alkaliphilus_metalliredigens       GSPIILQVSAGARKYAN-------PIYLRK--LVEAAVEDTD-LP----------IVLHLDHGDSFEICKQCIDDGFTS-VMIDA-------SHHSFEEN
Veillonella_sp                     QSPLILQVSAGARKYAK-------HIYLVK--LVEAALEDSN-LP----------IALHLDHGDSFEICKDCVDGGFTS-VMIDA-------SHHDFDEN
Acetonema_longum                   QSPLILQVSAGARKYAR-------HIYLMK--LAEAAVEDSG-LP----------ICLHLDHGEDFEICKSCIDGGFTS-VMIDG-------SKFPFEEN
Thermosinus_carboxydivorans        QAPLILQVSAGARKYAK-------HIYLMK--LVEAAVEDSG-LP----------ICLHLDHGEDFEICKACIDGGFTS-VMIDG-------SKLPFEEN
Pelosinus_fermentans               QAPLILQVSAGARKYAK-------HIYLTK--LVEAALEDSG-LP----------IALHLDHGDDFEICKSCIDGGFSS-VMIDG-------SKYSFEEN
Desulfosporosinus_sp               NAPLILQVSAGARKYAR-------HIYLMK--LVEAAVEDTG-LP----------ICLHLDHGEDFEICKACVDGGFTS-VMIDG-------SKLPFAEN
Desulfosporosinus_acidiphilus      NAPLILQVSAGARKYAR-------HIYLIK--LVEAAVEDTG-LP----------VALHLDHGEDFEICKACVDGGFTS-VMIDG-------SKLPFAEN
Acidaminococcus_sp                 ESPLILQVSAGARKYAK-------HAYLLH--LVQAALEDTD-LP----------IALHLDHGADFEICKACVDGGFTS-VMIDG-------SKYPFEEN
Acidaminococcus_intestini          ESPLILQVSAGARKYAK-------HAYLLH--LVQAALEDTD-LP----------IALHLDHGADFEICKACVDGGFTS-VMIDG-------SKYPFEEN
Phascolarctobacterium_succinat     KSPLILQVSAGARKYAN-------HIYLMK--LVEAAIEDTG-LP----------ICLHLDHGEDFDICKACIDGGFTS-VMIDG-------SKHPFEEN
Caldicellulosiruptor_kristjans     QAPLILQVSAGARKYAK-------HIYLIK--LVEAALEDSGDLP----------IALHLDHGEDFEICKACIDGGFTS-VMIDG-------SRLPFEEN
Caldicellulosiruptor_lactoacet     QAPLILQVSAGARKYAK-------HIYLIK--LVEAALEDSGDLP----------IALHLDHGEDFEICKACIDGGFTS-VMIDG-------SRLPFEEN
Tepidanaerobacter_acetatoxydan     KAPLILQVSAGARKYAK-------PLYLKK--LVEAAVHDSG-LP----------IVLHLDHGESFEICKSCIDDGFTS-VMIDG-------SKYPFEEN
Thermosediminibacter_oceani        KAPLILQVSAGARKYAR-------PIYLKK--LVEAAVEDTG-IP----------IVLHLDHGEDFEICKACIDDGFTS-VMIDG-------SRLPFEEN
Caloramator_australicus            KAPVILQVSASALKYAN-------PKYLIK--MVEAAVEDSG-LP----------IALHLDHGENFEICKTCIDLGFTS-VMIDA-------SKYPLEEN
Eubacterium_saphenum               EAPLILQVSAGARKYAN-------PIYLKK--LVEAAIEDTG-LD----------MALHLDHGEDFDICKQCVDGGFSS-VMVDG-------SKHPFEEN
Eubacterium_infirmum               NAPLILQVSAGARKYAK-------PAYLLK--LVEAAIEDTG-LD----------IALHLDHGEDFDICKKCIDDGFTS-VMIDG-------SKHPFEEN
Acetobacterium_woodii              QAPLILQVSAGARKYAN-------PVYLRK--LAEAAVEVSG-LD----------ICLHLDHGDDFDICKACVDSGFTS-VMIDG-------SKHPFSEN
Flavonifractor_plautii             CAPMILQVSKGARAYAN-------HTYLVK--LVEAAVIECPEIP----------IALHLDHGPDFETCKSCIDGGFTS-VMIDA-------SSKPFAEN
Oscillibacter_valericigenes        SAPLILQVSKGARAYAN-------HTYLVK--LVEAAVIECPNIP----------IVLHLDHGPDFETCKSCIDGGFTS-VMIDA-------SGKPFAEN
Bryantella_formatexigens           NSPVILQVSKGARAYAN-------HTYLVK--LVEAAVQENPQIP----------IALHLDHGDTFELCKSCIDGGFTS-VMIDA-------SSKSFEDN
```

```
Stomatobaculum_longum               KSPVILQASAGARKYAN-------SIYLVK--LVEAAVELHPEIP----------IVLHLDHGADFATCKDCIDGGFTS-VMIDY-------SGHSFEEN
Selenomonas_sputigena               KSPIILQASAGARKYAK-------PAYLKH--LVQAALECE-DLP----------IALHLDHGPDFETCKSCIDDGFTS-VMIDG-------SHLSFKEN
Megasphaera_elsdenii                NSPIILQASAGARKYAK-------PPYLKH--LVEAALEDH-DLP----------IALHLDHGADFETCKDCIDGGFTS-VMIDG-------SKHSFEDN
Mitsuokella_multacida               NSPVILQASAGARKYAK-------GPYLRH--LVEAALEVN-DIP----------IALHLDHGPDFETCKACIDDGFTS-VMFDG-------SKYEFKEN
Selenomonas_infelix                 KSPLILQVSAGARKYAR-------HEYLVH--LVKAALEIN-DIP----------IALHLDHGADFDICKSCIDGGFTS-VMIDG-------SHLPYDEN
Centipeda_periodontii               KSPLILQVSAGARKYAR-------HEYLVH--LVKAALEIN-NIP----------IALHLDHGADFDICKSCIDGGFTS-VMIDG-------SHLPYAEN
Megamonas_funiformis                NAPLILQCSAGARKYAK-------HQYLVH--LVQAAIEDT-GLP----------IALHLDHGADFETCKSCIDGGFTS-VMFDG-------SHYSFKEN
Anaeroglobus_geminatus              NSPVILQASAGARKYAK-------PTYLKH--LVEAALEEYPHLP----------VALHLDHGADFETCKDCIDGGFTS-VMIDG-------SQYSFDKN
Clostridium_thermocellum            NAPLILQVSAGARKYAN-------HTYLVK--LVEAAVEET-GLP----------ICLHLDHGDSFELCKSCIDGGFTS-VMIDG-------SHLPFEEN
Acetivibrio_cellulolyticus          NAPLILQVSAGARKYAN-------HTYLMK--LIDAAIIET-GLP----------ICVHLDHGDTFELCKSCIDGGFTS-VMIDG-------SHHSFEDN
Dictyoglomus_turgidum               RSPLILQISKGARNYAK-------LIYLMK--LIEAAVQDAPDIP----------IVVHLDHGDSVELCKEVIDAGFTS-VMIDG-------SHLPFEEN
Dictyoglomus_thermophilum           RSPLILQISKGARNYAK-------LVYLMK--LIEAAVQDAPDIP----------IVVHLDHGDSVELCKEVIDAGFTS-VMIDG-------SHLPFEEN
Thermotoga_lettingae                KAPLILQISAGARKYAK-------QVYLMK--LIEAAVEDAPDVP----------VCVHLDHGDSFELCKTVIDAGFTS-VMIDG-------SHLPFEEN
Thermotoga_thermarum                RAPVILQISAGARKYAK-------QIYLIK--LIEAALEDAPDIP----------IAVHLDHGDSFELCKAVIDAGFNS-VMIDG-------SHLPFEEN
Caldisericum_exile                  RSPVILQISKGARNYAK-------LVYLMK--LIEAATQDAPEVP----------IAVHLDHGDSFELCKEVIDAGFTS-VMIDG-------SHLPFEEN
Bilophila_sp                        KAPLILQVSAGARKYAG-------QNYIVK--LIEAGLLEA-DLP----------VVLHLDHGADFDICKACVDGGFTS-VMIDG-------SHHSFEEN
Bilophila_wadsworthia               KAPLILQVSAGARKYAG-------QNYIVK--LIEAGLLEA-DLP----------VVLHLDHGADFDICKACVDGGFTS-VMIDG-------SHHSFEEN
Lawsonia_intracellularis            RAPVILQVSAGARNYAG-------KNYIVK--LIEAGVLDT-DIP----------IVLHLDHGADFAICKTCIDDGFTS-VMIDG-------SHLPFEEN
Desulfohalobium_retbaense           RSPLILQVSAGARRYAG-------QTYILK--LVEAALEES-DLP----------ICLHLDHGQDFDICKKVIDGGFTS-VMIDG-------SHLPFEEN
Desulfomicrobium_baculatum          KSPLILQVSAGARRYAG-------QGYIIK--LMEAALAEN-DLP----------VCLHLDHGQNFEICKEVIDGGFTS-VMIDG-------SHLSFEDN
Desulfovibrio_alaskensis            KAPLILQVSAGARKYAG-------QTYIVK--LIEAALSES-DLP----------VVLHLDHGSGFDICKDCIDGGFTS-VMIDG-------SHLPYEEN
Desulfonatronospira_thiodismut      QSPLILQVSAGARKYAG-------QNYIMK--LMEAALKES-DLP----------VVLHLDHGQNFEICRDVISDGFTS-VMIDG-------SHLPFEEN
Desulfovibrio_piger                 KSPVILQVSSGARSYAG-------QKYLMK--LVEAALAEDPSVP----------VVVHLDHGSSFELCRDCIDGGFTS-VMIDG-------SHLPYEEN
Dialister_succinatiphilus           ESPVILQASEGARNYAG-------QEYIVG--LVKIALQDYPEIP----------TALHLDHGSSYEICKACIDGGFSS-VMYDG-------SKHPFEEN
Dialister_micraerophilus            KSAVILQASEGARQYAG-------QDYIVS--LIKTASAQYPEIP----------VAIHLDHGSCFEVCKECIDGGFTS-VMYDG-------SRHDFEKN
Clostridium_botulinum               NSAVILQVSAGALKYAG-------PKYLKA--MVDAAIADTG-ID-----------VALHLDHGASLDVVKLAVESGFTS-VMFDG-------SHYDYEEN
Peptostreptococcus_anaerobius       NSAVIIQASKSAISYAG-------MDTLVA--MVKAASEEIG-VD-----------CALHLDHGPSFEVAKKCIDAGFTS-VMIDG-------SHLDYEEN
Arthromitus_sp                      NSAVILQCSTGAIKYAG-------IDYLIS--LVKTAAEESG-LD-----------IALHLDHGPDFETCKKCIDAGFTS-VMIDG-------SHHSFEEN
Leptonema_illini                    RSPVILQVSAGARKYAG-------HDYLVK--MVEAAATTD--VP----------IALHLDHGADFEICKAAIDGGFNS-VMIDG-------SHHGFEEN
Anaerofustis_stercorihominis        NAPVILQVSAGARKYAK-------SVYLTS--LVDAAIKDTG-VD-----------VVLHLDHGDSFELCKSCIDDGFTS-VMIDA-------SKYDLEKN
Methanocella_arvoryzae              KSPVILAVSPSAIKYAG-------IEYIYE---IARVTAVKSKVP----------TVLHLDHGTEFKDCVQCIRHGWSS-VMYDG-------SKLPFQEN
Methanocella_conradii               RSPVILATSPSAIKYAG-------IEYIYE---IARVTAAKSGVP----------TVLHLDHGTTFEDCVKCIRHGWSS-VMFDG-------SKLPLEEN
Staphylococcus_lugdunensis          KAPVILGVSEGAARYMS-------GFYTVVKMVEGLIHDLNITIP----------VAIHLDHGSSFDKCKEAIDAGFTS-VMIDA-------SHSPFEEN
Staphylococcus_aureus               NAPVILGVSEGAARYMS-------GFYTIVKMVEGLMHDLNITIP----------VAIHLDHGSSFEKCKEAIDAGFTS-VMIDA-------SHSPFEEN
Listeria_grayi                      KSPVILGVSEGAAKYMG-------GFTTVVKMTEGLVEDLKITVP----------VAIHLDHGSSFDSCKAAIDAGFSS-VMIDG-------SHHPIDEN
Brevibacillus_brevis                KSPVIFGVSEGALKYMG-------IDYTVA---IARVAAERAGVP----------VALHLDHGSNFDMVMKCIRAGFSS-VMFDG-------SHHSFEDN
Kyrpidia_tusciae                    RSPVILGTSEGAIKYMG-------IDYTAA---IALTAARNATVP----------VVLHLDHGSSYEWVLKCLRRGWSS-IMIDG-------SHHPLEEN
Halanaerobium_praevalens            NSPVILQASEGAIRYIG-------MDYVMQ---MVEAATKNTEIP----------VALHLDHGSDFESIMKCIRAGFSS-VMIDA-------SKLPFAEN
Halanaerobium_hydrogeniformans      NSPLILQASEGAIRYIG-------MDYVIK---MVEAATDKTSIP----------VALHLDHGSSFESIMNCIRAGFSS-VMIDA-------SKKEFEEN
Halothermothrix_orenii              NSPLILQTSEGAIKYIG-------IDYVIG---MVEAATKNTTIP----------VALHLDHGSSFEVVMKCIRHGYSS-VMIDG-------SHYPFEEN
Thermoanaerobacter_wiegelii         ESPLILQTTPGTIKHAG-------EDYIAA---MAEVASEKYDIP----------IALHLDHGNSFDLVVKCIRAGYTS-VMIDG-------SMLPYEEN
```

```
Helicobacter_pylori             NSPLFIQASEGAIKYMG-------IDMAVG--MVKTMCERYPHIP----------VALHLDHGTTFESCEKAVKAGFTS-VMIDA-------SHHAFEEN
Helicobacter_acinonychis        NSPLFIQASEGAIKYMG-------IDMTVG--MVKIMCERYPHIP----------VALHLDHGTTFESCEKAVKAGFTS-VMIDA-------SHHAFEEN
Thiovulum_sp                    SSPIIVEASEGAIKYAG-------AEMLYL--MTKNLSEKYPHIP----------VALHLDHGTSVESCVQAIRAGFTS-VMIDA-------SHDSFEEN
Fervidobacterium_nodosum        RAPLIIETSEGAIKYAGNGDIHKGAKYFVE--LVRLFADSV-DIP----------IALHLDHGKHFEYIIAAIKAGYSS-VMIDA-------SEEPFEEN
Thermosipho_melanesiensis       KAPIIIETSEGAIKYAGNGNPLRGARFFAE--TVKNYAESL-DIP----------VALHLDHGKNLEYIAAAIKAGYSS-VMIDA-------SHEEFEKN
Marinitoga_piezophila           NSPVIIETSQGAMKYAGNGDFRKGAELFVK--MVKTFADEV-DIP----------VALHLDHGKDFEYIVTAIKAGYSS-VMIDA-------SEHPFEEN
Mesotoga_prima                  HSPVIIETSEGAIKYAGNGDIKLGAKLFVS--MVESYARNV-DIP----------VSLHVDHGKNFKIIMAAIQAGYSS-VMIDA-------SEFPFEKN
Aquifex_aeolicus                QAPVFIQTTESAIKYAG-------IEYLAG--MVHAVKDKY-SIP----------FALHLDHGKHVETILLAIRHGYTS-VMIDY-------SDKSYEEN
Truepera_radiovictrix           RSPVLLALSEGAIKYGKRQ-----LVDMVI------HEAKRATVP----------VAVHLDHGSSYESCMQCIEWGFSS-VMIDK-------SHEDEATN
Thermus_thermophilus            KSPVILALSEGAMKYGGRA-----LTRMVV------ALAQEARVP----------VAVHLDHGSSYESVLKALREGFTS-VMIDK-------SHEDFETN
Meiothermus_silvanus            RSPVLLALSEGAIKYGGKA-----LATLVR------ELGRDASVP----------VCIHLDHGSSYESCLRAIQMGFTS-VMIDK-------SHEDTETN
Deinococcus_deserti             RSPVMVQMSEGAIKYGGQD-----LANIVI------DLAERATVP----------VALHLDHGSSYESALKAIKMGFTS-VMIDA-------SHHQFAEN
Meiothermus_ruber               RAPVFIQVSDGARKYAGLEN----LSNLVR------DMASRVSVP----------VVLHLDHGADYQMVLQALRAGFTS-VMIDA-------SHHPFEEN
Thermus_scotoductus             RAPVFIQVSDGARKYAGMEN----LANLVK------DMASRTKVP----------VVLHLDHGADFKMVMQALRAGFTS-VMIDA-------SHHPFEEN
Giardia_lamblia                 KSPVILQCSRGALKYSD-------MIYLKK--LCEAALEKHPDIP----------ICIHLDHGDTLESVKMAIDLGFSS-VMIDASHHP-------FDEN
Bacterium_phylotype_RsD17       QSPVIIQASRGALKYSN-------FTYLGY--LMKAAIIENPDIP----------IVMHLDHGNSLESAIKAIDLGFSS-VMIDGSLLEDGKTASTYDYN
Eikenella_corrodens             NAPVIVQASAGARKYAG-------APFLRH--LILAAVEEFPHIP----------VVMHQDHGASPDVCQRSIQLGFSS-VMMDGSLLADGKTPSSYEYN
Kingella_kingae                 NAPVIVQASAGARKYAG-------APFLRH--LILAAVEEFPHIP----------VVMHQDHGASPDVCQRSIQLGFSS-VMMDGSLLEDGKTPSTYEYN
Neisseria_shayeganii            NAPVIVQASAGARKYAG-------APFLRH--LILAAVEEFPHIP----------VVMHQDHGTSPDVCQRSIQLGFSS-VMMDGSLKSDGKTPADYDYN
Laribacter_hongkongensis        DAPVIVQASAGARKYAG-------APFLRH--MILAAVEEFPHIP----------VVMHQDHGTSPDVCQRSIQLGFSS-VMMDGSLKSDGKTPADYDYN
Acinetobacter_baumannii         NSPVIVQASAGARKYAG-------APFLRH--LILAAIEEWPHIP----------VVMHQDHGTSPDVCQRSIQLGFSS-VMMDGSLGADGKTPTTYDYN
Acinetobacter_sp                NSPVIVQASAGARKYAG-------APFLRH--LILAAVEEWPHIP----------VVMHQDHGTSPDVCQRSIQLGFSS-VMMDGSLGSDGKTPTSYDYN
Marinomonas_mediterranea        NSPVIMQASAGARKYAG-------SHFLRH--LIAAAIEDFPHIP----------VVMHQDHGTSPAICQRSIQLGFSS-VMMDGSLMADGKTPASYEYN
Oceanospirillum_sp              NSPVIVQASAGARKYAG-------SNFLRH--MIEAAIAEFPHIP----------VCMHQDHGTSPAVCQRTIAMGFSS-VMMDGSLMSDGKTPSSYEYN
Marinobacterium_stanieri        DSPVIVQASAGARKYAG-------SNFLRH--MILAAIEEFPDVP----------VCMHQDHGTSPAVCQRSIAMGFSS-VMMDGSLGEDGKTPTDYAYN
Oceanobacter_sp                 DSPVIVQASAGARNYAG-------APFLRH--LILAAVEEFPHIP----------VCMHQDHGTSPAVCQRSIQLGFSS-VMMDGSLGEDGKTPTEYDYN
Colwellia_psychrerythraea       DSPVIMQASAGARKYAG-------APFLRH--LILAAIEEFPHIP----------VVMHQDHGTSPTICQRSIQLGFSS-VMMDGSLMDDGKTPSSYEYN
Hahella_chejuensis              DSPVIVQASAGARKYAG-------APFLRH--LILAAIEEFPHIP----------VVMHQDHGTSPAVCQRSIQLGFSS-VMMDGSLGEDGKTPTTYEYN
Alishewanella_aestuarii         DSPVIVQASAGARKYAG-------APFLRH--LILAAVEEFPHIP----------VVMHQDHGTSLAVCQRSIQLGFSS-VMMDGSLGEDGKTPMDYAYN
Alishewanella_jeotgali          DSPVIVQASAGARKYAG-------APFLRH--LILAAVEEFPHIP----------VVMHQDHGTSLAVCQRSIQLGFSS-VMMDGSLGEDGKTPMDYAYN
Rheinheimera_nanhaiensis        DSPVIVQASAGARKYAG-------APFLRH--LILAAVEEFPHIP----------VVMHQDHGTSLAVCQRSIALGFSS-VMMDGSLGEDGKTPMDYAYN
Congregibacter_litoralis        DSPVIMQASAGARKYAG-------APFLRA--LMEAAMREFPHIP----------VVVHQDHGMSPAICQRSIALGFSS-VMMDGSLGEDGKTPMDYDYN
Pseudoalteromonas_marina        NSPVIVQGSAGARAYAG-------APFIRH--MILAAVEEWPHIP----------VVMHQDHGTSPGVCQRSIQLGFSS-VMMDGSLMSDGKTPSSYEYN
Alteromonadales_bacterium       NSPVIVQGSAGARAYAG-------APFIRH--MILAAVEEWPHIP----------VVMHQDHGTSPGVCQRSIQLGFSS-VMMDGSLMSDGKTPSSYEYN
Idiomarina_baltica              NSPVIVQASAGARKYAG-------APFLRH--LIESAVEEWPHIP----------VVVHQDHGTSPAVCQRSIQLGFSS-VMMDGSLLDDGKTPADYDYN
Shewanella_baltica              DSPVIVQASAGARKYAR-------PQFLKY--LMAAALEQYPDIP----------VCIHQDHGTDPDICQRSIQLGMSS-VMMDGSLMADGKTPASYDYN
Shewanella_violacea             DSPVIVQASAGARKYAR-------PQFLKY--LMAAALEQYPDIP----------VCIHQDHGTDPDICQRSIQLGMSS-VMMDGSLMADGKTPASYDYN
Ferrimonas_balearica            DSPVIVQASAGARKYAR-------PQFLRY--LMNAALEQYPDVP----------VCIHQDHGTSPDVCQRSIQMGFSS-VMMDGSLREDGKTPADYGYN
Pseudomonas_mendocina           DSPVIVQASAGARKYAG-------APFLRH--LILAAIEEFPHIP----------VCMHQDHGTSPDVCQRSIQLGFSS-VMMDGSLKEDGKTPADYDYN
Pseudomonas_fulva               DSPVIVQASAGARKYAG-------APFLRH--LILAAIEEFPHIP----------VCMHQDHGTSPDVCQRSIQLGFSS-VMMDGSLKEDGKTPADYEYN
Azotobacter_vinelandii          DSPVIVQASAGARKYAG-------APFLRH--LILAAIEEFPHIP----------VCMHQDHGTSPDVCQRSIQLGFSS-VMMDGSLKEDGKTPADYDYN
```

```
Halomonas_sp                        DSPVIVQASAGARKYAG-------APFLRH--LILAAVEEFPHIP----------VVMHQDHGTSPAVCQRSIQLGFSS-VMMDGSLGEDGKTPMDYDYN
Halomonas_boliviensis               DSPVIVQASAGARKYAG-------APFLRH--LILAAVEEFPHIP----------VVMHQDHGTSPAVCQRSIQLGFSS-VMMDGSLGEDGKTPTDYDYN
Chromohalobacter_salexigens         DSPVIVQASAGARKYAG-------APFLRH--LIQAAVEEFPHIP----------VVMHQDHGTSPGVCQRSIQLGFSS-VMMDGSLKEDGKTPADYAYN
Marinobacter_algicola               DSPVIVQASAGARKYAG-------APFLRH--LILAAIEEFPHIP----------VVMHQDHGTSPSVCQRSIQLGFSS-VMMDGSLGEDGKTPTDYEYN
Marinobacter_aquaeolei              DSPVIVQASAGARKYAG-------APFLRH--LILAAIEEWPHIP----------VVMHQDHGTSPAVCQRSIQLGFSS-VMMDGSLGEDGKTPTSYEYN
Thiocystis_violascens               DSPVIVQASAGARKYAG-------APFLRH--LILAAIEEWPHIP----------VCMHQDHGTSMPVCQRSIQLGFSS-VMMDGSLQEDGKTPASYEYN
Thiocapsa_marina                    DSPVIVQASAGARKYAG-------APFLRH--LILAAIEEWPHIP----------VCMHQDHGTSAAVCQRSIQLGFSS-VMMDGSLGEDGKTPTSYEYN
Allochromatium_vinosum              DSPVIVQASAGARKYAG-------APFLRH--LILAAIEEWPHIP----------VCMHQDHGTSPAVCQRSIQLGFSS-VMMDGSLGEDGKTPTSYEYN
Thiorhodococcus_drewsii             NSPVIVQASAGARSYAG-------APFLRH--LILAAIEEWPHIP----------VCMHQDHGTSMPVCQRSIQLGFSS-VMMDGSLGEDGKTPTSYEYN
Marichromatium_purpuratum           DSPVIVQASAGARKYAG-------APFLRH--LILAAIEEWPHIP----------VCMHQDHGTSPAVCQRSIQLGFSS-VMMDGSLGEDGKTPTSYDYN
Thiomonas_intermedia                NSPVILQASAGARKYAG-------APFLRH--LIEAAVEEWPHIP----------VVMHQDHGTSPGVCQRSIQLGFSS-VMMDGSLGTDGKTPTTYEYN
Francisella_novicida                NSPVILQGSAGARKYAG-------ASFIRH--LVLAAIEEYPHIP----------VCMHQDHGTSPSVCQRSIQLGFSS-VMMDGSLKSDGKTPADYEYN
Francisella_noatunensis             NSPVILQGSAGARKYAG-------APFIRH--LVLAAIEEYPHIP----------VCMHQDHGTSLSVCQRSIQLGFSS-VMMDGSLKSDGKTPSDYEYN
Kangiella_koreensis                 DSPVIIQASAGARKYAG-------SNFLRH--LIVAATEEFPHIP----------ICMHQDHGSSPAVCQQSIQLGFSS-VMMDGSLEEDMKTPASYEYN
Coxiella_burnetii                   KSPVIVQASAGARQYAG-------PQFIRA--LIVAATEEWPHIP----------ICMHQDHGASPAVCQRSIQLGFTS-VMMDGSLLEDQKTPSDYEYN
Thiothrix_nivea                     NSPVIMQGSAGARGYAG-------EPFLRH--LILAAIEMYPHIP----------VCMHQDHGAEPAVCFRSIQSGFSS-VMMDGSLMADGKTPSSYEYN
Thiobacillus_denitrificans          DSPVILQGSAGARSYAG-------EPFLRH--LILAAIEMYPQIP----------ICMHQDHGASPSICFRSIQSGFSS-VMMDGSLKEDGKTPASYEYN
Thioalkalimicrobium_cyclicum        NSPVILQGSAGARKYAG-------EPMLRH--MVAAAVEMYPHIP----------VVMHQDHGSDVGVCLRAIQSGFTS-VMMDGSLMSDMKTPASYEYN
Halothiobacillus_neapolitanus       DSPVILQGSAGARKYAG-------EPFLRH--LVSAAVEMYPHIP----------VVMHQDHGADPGVCFRAIQSGFSS-VMMDGSLMPDMKTPSSFEYN
Nitrosococcus_watsonii              DSPAIMQASAGARSYAG-------EPFLRH--LIEAAVEQYPHIP----------ICMHQDHGAEPAVCLRSIQSGFSS-VMMDGSLMADMKTPSSYEYN
Halorhodospira_halophila            DSPAIVQASAGARKYAG-------VPFYRH--LMEAAVESYPDVP----------LVVHLDHGANPGACMRAIQSGFTS-VMMDGSLKEDGKTPADYDYN
Alkalilimnicola_ehrlichii           DSPVILQSSAGARKYAG-------EPFFRH--MMHAAVEQYPDIP----------LCIHQDHGASPAVCLRSIQSGFTS-VMMDGSLREDMKTPSDYEYN
Rubrivivax_benzoatilyticus          DAPVILQASAGARKYAG-------EPFLRK--LVEAAVEQYPATP----------ICLHQDHGASPAVCVQAIRSGFTS-VMMDGSLREDQKTPASYDYN
Rubrivivax_gelatinosus              DAPVILQASAGARKYAG-------EPFLRK--LVEAAVEQYPATP----------ICLHQDHGASPAVCVQAIRSGFTS-VMMDGSLREDQKTPASYDYN
Hydrocarboniphaga_effusa            DSPVILQASAGARKYAG-------EAFLRR--LVEAAVESYPHLP----------ICMHQDHGASPAVCQASIRSGFSS-VMMDGSLREDMKTPASYDYN
Dechloromonas_aromatica             DSPVILQASAGARKYAG-------EPFLRK--LVEAAIEQYPDIP----------VCLHQDHGTSPAVCQQSMRSGFSS-VMMDGSLGPDGKTPANYAYN
Oxalobacteraceae_bacterium          DSPVILQASAGARNYAG-------EVFLRK--LVEGAIESYPHIP----------VCMHQDHGASAAVCQAAMRSGFSS-VMMDGSLLADMKTPSSYEYN
Leptothrix_cholodnii                MSPVILQASAGARKYAG-------EAYLRH--LVLAAIESHPDIP----------VVLHQDHGASAAVCVQAIRSGFSS-VMMDGSLMEDAKTPASYDYN
Methylovorus_glucosetrophus         DSPVILQASAGARKYAG-------EIFLQH--LVLAAIETYPHIP----------VVMHQDHGASADVCVQAIRLGFSS-VMMDGSLREDASTPADFAYN
Methylobacillus_flagellatus         HSPVILQASAGARKYAG-------EAFLLK--LAEAAIASYPHLP----------VVLHQDHGASPAICLHAIQSGFSS-VMMDGSLLEDGKTPASYEYN
Methylotenera_versatilis            NSPVILQGSAGARKYAG-------EAFLRH--LVLAAIETYPHIP----------VVMHQDHGQSPAVCIQAIRSGFSS-VMMDGSLMEDAKTPSTYQYN
Nitrosospira_multiformis            DSPVIMQGSAGARKYAG-------EPFLRH--LIAAAVEAYPHIP----------IVMHQDHGASPAVCVNAIRSGFSS-VMMDGSLEADAKTPSSYEYN
Gallionella_capsiferriformans       NSPVIMQGSAGARKYAG-------EPFLRH--LIEAAVEMYPHIP----------VVMHQDHGASEAVCINAIRSGFSS-VMMDGSLLADGKTPSSFEYN
Methylomirabilis_oxyfera            DSPVIMQASAGARKYAG-------EPFLRH--LFLAASEMYPDIP----------VVVHQDHGASPAVCIASIRSGFSS-VMMDGSLLADGKTPSSYDYN
Acidithiobacillus_caldus            DAPVILQASAGARKYAG-------EPFLRH--LILAAIEEYPDIP----------VVLHQDHGASPAVCIQAIRSGFSS-VMMDGSLMEDAKTPASYDYN
Magnetospirillum_magneticum         DAPVILQASAGARKYAG-------EPFLRH--LILAAIEAYPQIP----------VCMHQDHGTSPAINVRAIQSGFSS-VMMDGSLREDGKTPSDWDYN
Magnetospirillum_magnetotactic      DAPVILQASAGARKYAG-------EAFLRH--LILAAIETYPQIP----------VCMHQDHGTSPAINVRAIQSGFSS-VMMDGSLKEDGKTPSDWDYN
Phaeospirillum_molischianum         DSPVILQASAGARKYAG-------EAFLRH--LILAATEAYPQIP----------VCMHQDHGTSPSINVRAIQSGFSS-VMMDGSLKEDGKTPSDWDYN
Aromatoleum_aromaticum              DSPVIMQASAGARKYAG-------EPFLRH--LIDAAIEAYPQIP----------VVMHQDHGQSPAICMAAIRSGFSS-VMMDGSLLEDGKTPSSYDYN
Thauera_sp                          DAPVIMQASAGARKYAG-------EAFLRH--LIDAAIEAYPHIP----------VVMHQDHGQSPAICMGAIRSGFSS-VMMDGSLLEDGKTPSSYEYN
Azoarcus_sp                         DSPVIMQASAGARKYAG-------EAFLRH--LIDAAVEAYPHIP----------VVMHQDHGQSPAVCMAAIRSGFSS-VMMDGSLMEDGKTPSSYEYN
```

```
Dechlorosoma_suillum                NAPVIMQASAGARKYAG-------EAFLRH--QILAALEAYPHIP----------VVMHQDHGQSPAVCMAAIKSGFSS-VMMDGSLQEDGKSVASYDYN
Accumulibacter_phosphatis           DAPVIMQASAGARKYAG-------EPFLRH--QILAALEAYPHIP----------IVMHQDHGQSPAVCMGAIRSGFSS-VMMDGSLMADGKSVASYEYN
Herbaspirillum_sp                   GAPVIMQASAGARKYAG-------EAFLRH--LISAAVEAYPHIP----------VVMHQDHGQSPAVCMAAIRSGFSS-VMMDGSLEADGKSVASYEYN
Herbaspirillum_seropedicae          GAPVIMQASAGARKYAG-------EAFLRH--LISAAVEAYPHIP----------VVMHQDHGQSPAVCMAAIKSGFSS-VMMDGSLEADGKTVASYEYN
Collimonas_fungivorans              GAPVIMQASAGARKYAG-------EAFLRH--LIEAAVEAFPHIP----------VVMHQDHGQSPAVCMAAIKSGFTS-VMMDGSLMEDGKSVASYEYN
Herminiimonas_arsenicoxydans        GAPVIMQASAGARKYAG-------EPFLRH--LIAAAVEAYPHIP----------VVMHQDHGQTPAVCMAAIRSGFTS-VMMDGSLNADGKSVATYEYN
Polynucleobacter_necessarius        DSPVIMQASAGARKYAG-------EAFLRH--LISAAVEAYPHIP----------VVMHQDHGQSPAVCMAAIKSGFTS-VMMDGSLEADGKTVASYEYN
Janthinobacterium_sp                NSPVIMQASAGARKYAG-------EAFLRH--LIDAAVEAYPHIP----------VVMHQDHGQSPAVCMAAIRSGFTS-VMMDGSLEADGKSVASYEYN
Burkholderia_cenocepacia            GAPVIMQASAGARKYAG-------EPFLRH--LIEAAVESYPHIP----------VVMHQDHGQSPAVCTAAIRSGFTS-VMMDGSLEADGKTVASYEYN
Oxalobacter_formigenes              DSPVIMQASAGARRYAG-------EAFLRH--LIEAAVEAYPDIP----------IVMHQDHGQSPAVCMSAIRSGFSS-VMMDGSLLPDGKTAADYDYN
Ralstonia_eutropha                  NAPVIMQASAGARKYAG-------EHFLRH--LIEAAVEAYPHIP----------VVMHQDHGQSPAICQAAIDLGFSS-VMMDGSLREDGKTPADYEYN
Cupriavidus_taiwanensis             NAPVIMQASAGARKYAG-------EHFLRH--LIEAAVEAYPHIP----------VVMHQDHGQSPAICQAAIDLGFSS-VMMDGSLREDGKTPADYEYN
Cupriavidus_necator                 NAPVIMQASAGARKYAG-------EHFLRH--LIEAAVEAYPHIP----------VVMHQDHGQSPAICQGAIDLGFSS-VMMDGSLREDGKTPAEYDYN
Acidovorax_citrulli                 GAPVILQASAGARKYAG-------EPFIKH--LIQAAAEMYPHIP----------LVMHQDHGTSPEVCQGALDLGFGS-VMMDGSLMSDGKTPSSFDYN
Comamonas_testosteroni              GAPVILQASAGARKYAG-------EPFIKH--LIQAAAEMYPHIP----------LVMHQDHGTTPEVCQGALNLGFGS-VMMDGSLMSDGKTPSSFDYN
Acidovorax_radicis                  GAPVILQASAGARKYAG-------EPFIKH--LIQAAVEAFPHIP----------LVMHQDHGTSPKICQGAIDLGFGS-VMMDGSLMEDGKTPSSFDYN
Limnobacter_sp                      GAPVILQASAGARKYAG-------EPFIKH--LIQAAVEAFPHIP----------LVMHQDHGTSPKVCQGAIDLGFGS-VMMDGSLMEDGKTPASFDYN
Hylemonella_gracilis                GAPVILQASAGARKYAG-------EPFIKH--LIQAAVEAFPHIP----------LVMHQDHGTTPKVCEGAIALGFGS-VMMDGSLKEDGKTPADFAYN
Methyloversatilis_universalis       GAPVILQASAGARKYAG-------ESFVKH--LIQAATEAWPHIP----------LVMHQDHGTSPKVCQGALDLGFGS-VMMDGSLMEDGKTPASFDYN
Alicycliphilus_denitrificans        GAPVILQASAGARKYAG-------ESFIKY--LILAAAEAYSHIP----------LVMHQDHGTSPAVCEGALKLGFGS-VMMDGSLKEDGKTPADFDYN
Delftia_sp                          GAPVILQASAGARKYAG-------EPFIKH--LIQAAAEQYPHIP----------LVMHQDHGTSPAICQGALNLGFGS-VMMDGSLMEDGKTPSSFEYN
Delftia_acidovorans                 GAPVILQASAGARKYAG-------EPFIKH--LIQAAAEQYPHIP----------LVMHQDHGTSPAICQGALNLGFGS-VMMDGSLMEDGKTPSSFEYN
Ralstonia_pickettii                 GAPVILQASAGARKYAG-------ESFIKH--LIQAAVEAYPEIP----------LVMHQDHGQSPAICQGAIDLGFGS-VMMDGSLREDGKTPADFDYN
Variovorax_paradoxus                GAPVILQASAGARKYAG-------EAFIKH--LIQAAIEQYPNIP----------LVMHQDHGQNPDVCKGAIDLGFSS-VMMDGSLEADGKTIASYDYN
Hydrogenophaga_sp                   GAPVILQASAGARKYAG-------EPFIKH--LIQAAIEMYPHIP----------LVMHQDHGQSPDVCQGAINLGFSS-VMMDGSLEADGKTIASYDYN
Ramlibacter_tataouinensis           GAPVILQASAGARKYAG-------EPFVKH--LILAAIEAYPGIP----------LVMHQDHGQSPDVCQGAINLGFSS-VMMDGSLEADGKTIASYDYN
Polaromonas_naphthalenivorans       GAPVILQASAGARKYAG-------EAFIKH--LILAAVEAYPHIP----------LVMHQDHGQSPAICQGAIDLGFSS-VMMDGSLEADGKTIASFDYN
Methylibium_petroleiphilum          GAPVILQASAGARKYAG-------ENFIKH--LIQAAIETWPHIP----------LVMHQDHGQSPDVCQGAIDLGFSS-VMMDGSLQADGKTIASYDYN
Achromobacter_xylosoxidans          DSPVIMQASAGARKYAG-------EGFLKY--LIQAAVETYPHIP----------VVMHQDHGQSPKICQGAIDLGFSS-VMMDGSLKEDGKTIADYDYN
Achromobacter_arsenitoxydans        DSPVIMQASAGARKYAG-------EGFLKY--LIQAAVESYPHIP----------VVMHQDHGQSPKVCQGAIDLGFSS-VMMDGSLKEDGKTIADYDYN
Bordetella_petrii                   DSPVIMQASAGARKYAG-------EGFLKY--LIQAAVESYPHIP----------VVMHQDHGQSPKVCQGAIDLGFSS-VMMDGSLEEDGKTIADYDYN
Bordetella_pertussis                DSPVIMQASAGARKYAG-------EGFLKY--LIQAAVESYPHIP----------VVMHQDHGQSPAVCQGAIDLGFSS-VMMDGSLKEDGKTIADYDYN
Advenella_kashmirensis              NSPVIMQASAGARKYAG-------EGFLKY--LIQAAVESYPEIP----------VVMHQDHGQSPEICQGAINLGFSS-VMMDGSLMADGKTIADYEYN
Pusillimonas_sp                     DSPVIMQASAGARKYAG-------EGFLKY--LIQAAVESYPHIP----------VVMHQDHGQSPAICKGAIDLGFSS-VMMDGSLKEDGKTIAEYEYN
Alcaligenes_faecalis                DSPVIMQASAGARKYAG-------EAFLRH--LIEAAVESYPHIP----------VVMHQDHGQSPEICKGAIELGFSS-VMMDGSLLPDGKTIADFEYN
Taylorella_equigenitalis            NSPVIMQASAGARKYAG-------EPFLKH--LIQAAVEAYPEIP----------IVMHQDHGQSPEVCRGAINLGFTS-VMMDGSLMPDGKTIATYEYN
Parasutterella_excrementihomin      NAPVIMQASAGARKYAG-------EVFLEH--LIKAAIESYPHIP----------VCMHQDHGQSPAVCQGAINLGFSS-VMMDGSLMSDGKTISTFDYN
Burkholderiales_bacterium           NAPVIMQASAGARKYAG-------EVFLEH--LIKAAIESYPHIP----------VCMHQDHGQSPAVCQGAINLGFSS-VMMDGSLMSDGKTISTFDYN
Sutterella_wadsworthensis           GAPVIMQASAGARKYAG-------EAFLRH--LIEAAVEAYPDVP----------VCMHQDHGQSPAVCMGAIRSGFTS-VMMDGSLMSDGKTISTYEYN
Xanthobacter_autotrophicus          RSPVILQGSAGARKYAG-------EAFLRH--LIAAAVEAYPEIP----------VVMHQDHGASPAVCMGAIKSGFSS-VMMDGSLKEDGKTPADYDYN
Magnetococcus_marinus               DSPVILQASAGARKYAG-------EAFLRH--QILAALELYPHIP----------ICMHQDHGQSPAICQAAIRSNFSS-VMMDGSLREDGKTPSDWDYN
```

```
Nostoc_punctiforme                  DSPVILQASRGARNYAG-------ENFLRH--LILAAVETYPHIP----------IVMHQDHGNAPATCYSAIKNNFTS-VMMDGSLEADAKTPASFEYN
Anabaena_variabilis                 DSPVILQASRGARNYAG-------ENFLRH--LILAAVETYPEIP----------IVMHQDHGNAPSTCYSAIKNNFTS-VMMDGSLEADAKTPASFEYN
Microcystis_aeruginosa              DSPVILQASRGARKYAG-------ENFLRH--LITAAVETYPHIP----------IVMHQDHGNEPATCYSAIRNGFTS-VMMDGSLEADAKTPATYEYN
Lyngbya_sp                          NSPVILQASRGARKYAG-------ENFLRH--LILAAIETYPHIP----------IAMHQDHGNSPATCYSAMRNGFTS-VMMDGSLEADAKTPASFEYN
Arthrospira_platensis               NSPVILQASRGARSYAG-------ENFLRH--LILAAVETYPHIP----------VAMHQDHGNSPATCYSAMRHGFTS-VMMDGSLEADAKTPSDFEYN
Oscillatoria_sp                     NSPVILQASRGARSYAG-------ENFLRH--LILAAVETYPHIP----------IVMHQDHGNAPGTCYSAIRNGFTS-VMMDGSLEADAKTPASYEYN
Cyanothece_sp                       DSPVILQASRGARQYAG-------ENFLRH--LILAAAESYPHIP----------IAMHQDHGNSPATCYSAIRNGFTS-VMMDGSLEADAKTPASFEYN
Synechococcus_elongatus             DSPVILQASRGARSYAG-------ENFLRH--LILAAVETYPHIP----------IAMHQDHGNSPATCYSAIKNGFTS-VMMDGSLEADAKTPASYEYN
Prochlorococcus_marinus             DSPVILQASRGARTYAG-------EIFLRH--LIIAATETYPNIP----------VVMHQDHGNDPSTCYSAAINGFTS-VMMDGSLEADAKTPSSYEYN
Paulinella_chromatophora            DSPVILQASRGARSYAG-------ENFLRH--LILAAVETYPDIP----------VVMHQDHGNSPATCFGAAANGFTS-VMMDGSLEADAKTPASYEYN
Synechococcus_sp                    NSPVILQASRGARKYAG-------EHFLRH--LILAAVETYPHIP----------VAMHQDHGNSPATCYSAIRNGFTS-VMMDGSLKEDAKTPASYEYN
Gloeobacter_violaceus               NSPVILQASRGARKYAG-------EAFLRH--MVLAAVEEYPEIP----------IVMHQDHGNSPATCYSAIKNGFTS-VMMDGSLMEDAKTPASYEYN
Sinorhizobium_meliloti              DSPVILQASRGARAYAG-------DAFLRH--LILGAAEEYPHIP----------VCLHLDHGDQPSTCISAITNGFTS-VMMDGSLEKDGKTVASYEYN
Stappia_aggregata                   DAPVIIQASRGARSYAH-------DVMLKH--MMDAVVEIYPHIP----------VCVHLDHGNAPQTCMTAIQAGFTS-VMMDGSLEADGKTPANWDYN
Labrenzia_alexandrii                DSPVIIQASRGARAYAH-------DVMLKH--MMDAVVEIYPHIP----------VCVHLDHGNAAQTCMTAIQAGFTS-VMMDGSLEADGKTPASWDYN
Polymorphum_gilvum                  DSPVIIQASRGARSYAH-------DVMLKH--MMDAVTEIYPHIP----------VCVHLDHGNEPATCMTAIQAGFTS-VMMDGSLTADGKTPADWTYN
Bradyrhizobium_sp                   DAPVIIQASRGARSYAN-------DIMLKH--MMDAVTEIYPHIP----------VCVHLDHGNEAATCMTAIQAGFTS-VMMDGSLKADGKSPADWTYN
Agrobacterium_radiobacter           DAPVIIQASRGARAYAN-------DIMLKH--MMDAVVEIYPHIP----------VCVHLDHGNDASSCMTAIQAGFTS-VMMDGSLKADAKTPADWDYN
Rhizobium_sp                        DAPVIIQASRGARAYAN-------DIMLKH--MMDAVVEIYPHIP----------VCVHLDHGNDASSCMTAIQAGFTS-VMMDGSLKADAKTPADWDYN
Brucella_melitensis                 DAPVILQASRGARSYAN-------DIMLRH--MMDAVTEIYPHIP----------VCVHLDHGNEAATCMSAIQAGFTS-VMMDGSLKADGKTPADWDYN
Brucella_suis                       DAPVILQASRGARSYAN-------DIMLRH--MMDAVTEIYPHIP----------VCVHLDHGNEAATCMSAIQAGFTS-VMMDGSLKADGKTPADWDYN
Ochrobactrum_anthropi               DAPVILQASRGARSYAN-------DIMLRH--MMDAVTEIYPHIP----------VCVHLDHGNEAATCMTAIQAGFTS-VMMDGSLKADGKTPADWDYN
Sinorhizobium_fredii                DSPVILQASRGARAYVN-------DIMLKH--MMEAVTEIYPHIP----------VCVHLDHGNEPSTCVTAIQHGFTS-VMMDGSILADGKTAADWDYN
Mesorhizobium_amorphae              KSPVILQASRGARAYAN-------DVVLAK--LIDALVEIHPDIP----------VCMHLDHGNNEATCVTAIQYGFTS-VMMDGSLKEDGKSPADYDYN
Mesorhizobium_loti                  KSPVILQASRGARAYAN-------DVVLAK--LIDALVEIHPDIP----------VCMHLDHGNNEATCVTAIQYGFTS-VMMDGSLKEDGKSPADYAYN
Methylobacterium_nodulans           DAPVILQASRGARAYAN-------DVILAK--LIDGLVEIYPHIP----------VCMHLDHGNNEATCATAIQYGFTS-VMMDGSLKADGKTPADYAYN
Methylobacterium_radiotolerans      DSPVILQASRGARAYAN-------DVVLAK--LIDGLVEIYPHIP----------VCMHLDHGNNEATCATAIQYGFTS-VMMDGSLKADGKTPADYTYN
Phenylobacterium_zucineum           DSPVIMQASRGARSYAN-------DIVLKH--LIDAMAEMYPHVP----------ICMHQDHGNNEATCATAIQYGFTS-VMMDGSLMADGKTPADYDYN
Caulobacter_sp                      NSPVIIQASRGARNYAN-------DIMLAR--MIDALAEIYPDIP----------VCMHQDHGNGPATCATAIQYGFTS-VMMDGSLMEDAKTPATYEYN
Caulobacter_crescentus              NSPVIIQASRGARNYAN-------DIMLAK--MIDALVDIYPHIP----------VCMHQDHGNGPATCATAIQYGFTS-VMMDGSLMEDAKTPASYEYQ
Paracoccus_denitrificans            DAPVILQASRGARSYAG-------DIMLKR--MVEALAEMNPGVP----------ICLHQDHGNNLATCMSAIRHGFTS-VMMDGSLHEDMKTPADYDYN
Paracoccus_sp                       DAPVILQASRGARSYAG-------DIMLRR--MVEALAEMNPTIP----------ICLHQDHGNNLATCMSAIRHGFTS-VMMDGSLKEDMKTPADYDYN
Pelagibaca_bermudensis              DAPVILQASRGARKYAG-------DIMLRH--MVQALAEMFPSNH----------IVMHQDHGNNDATCLSAIRHGFTS-VMMDGSLEEDMKTPASYAYN
Citreicella_sp                      DAPVILQASRGARKYAG-------DIMLRH--MVQALAEMFPGNH----------IVMHQDHGNNDATCLSAIRHGFTS-VMMDGSLEEDMKTPATYEYN
Rhodopseudomonas_palustris          DAPVIIQASRGARSYAN-------DIMLAK--MIDALAEMYPDIP----------LCMHQDHGNDEATCATAIKYGFTS-VMMDGSLKADAKTAADYEYN
Starkeya_novella                    DAPVIMQASRGARSYAG-------DIMLAR--MIDALTEMYPSIP----------ICMHQDHGNNEATCLSAIQHGFTS-VMMDGSLKADAKTPADYDYN
Azospirillum_amazonens              DAPVILQASRGARSYAG-------DLMLAH--MIDALEGLYPTIP----------LCLHQDHGNSEATCLTALQHGFTS-VMMDGSLMADAKTPAPYAYN
Rhodobacter_sphaeroides             DAPVIIQASRGARSYAN-------DIMLAK--MIEALAAIYPEIP----------LCMHQDHGNNEATCMTAIRHGFTS-VMMDGSLKADAKTPADYDYN
Aurantimonas_manganoxydans          DAPVIMQASRGARSYAK-------DIMLEK--MMEALVEMFPEIP----------VCIHQDHGNDEQTCLTAIRHGFTS-VMMDGSLEADMKTPASYEYN
Octadecabacter_arcticus             DSPVIMQASRGARSYAN-------DIVLSH--IIQAAIELYPHIP----------VCMHQDHGNSPATCLSAITHGYSS-VMMDGSLAADGKTPTNFQEN
Sulfitobacter_sp                    DSPVIMQASRGARAFAN-------DIVLGH--LIRAAIEMYPHLP----------ICMHQDHGNSPQTCLSALMNGFSS-VMMDGSLSEDGTTPTDFEYN
```

```
Rhodospirillum_photometricum    DSPVILQASRGARSYAG-------DIMIKH--MIQAVAEMYPDIP----------VCMHQDHGNSLGTCLSAISANFTS-VMMDGSLETDAKTPATYEYN
Pirellula_staleyi               DSPVIIQASRGARSYSQ-------DAYLRH--LMLAAAELYPNIP----------IVMHQDHGNSVATCLSAIDNGFTS-VMMDGSLKEDGKTPADFDYN
Blastopirellula_marina          DSPVIVQASRGARAYSQ-------DNYLRH--LMIAASELYPQIP----------IVMHQDHGNSPETCMSAIENGFTS-VMMDGSLKEDGKTPADFDYN
Rhodopirellula_baltica          DSPVIIQASRGARAYTQ-------DTYLRH--LMVAAAELYPQIP----------IVMHQDHGNSPETCLSAIENGFTS-VMMDGSLEADGQTPADYDYN
Koribacter_versatilis           QSPVIIQASRGARSYSQ-------DKFLYH--LMLAAAELYPEIP----------LVMHLDHGNSVETCKSAIDLGFTS-VMMDGSLMPDGKTPSTFEYN
Solibacter_usitatus             QSPVIIQASRGARAYSQ-------DRFLYH--LMIAATEIYPEIP----------TVLHLDHGNSVATCKSAIDMGFTS-VMMDGSLMEDGKTPSSFEYN
Anaerolinea_thermophila         QAPFIIQISKGARKYTD-------KRMLEA--IIRAADEIFPDAI----------FAVHLDHGDE-ETCMDAIESGFYSSVMIDA-------SHEDFETN
Diplosphaera_colitermitum       KAPFIIQISKGARKYTD-------KAMLEG--MIRSADSIFPEAI----------FAVHLDHGDE-ETCYDCINSGFYSSVMIDA-------SHDPFEKN
CONSERVATION                                                                                ***                 * *

                                        210       220       230       240       250       260       270       280       290       300
                                ....|....|....|....|....|....|....|....|....|....|....|....|....|....|....|....|....|....|....|....|
Chlorobium_phaeovibrioides      VALTKKVVEYAHQHD---VTVEGELGVLAGIEDEVS------------AEHHTYTQPEEVEDFVAKTGVDSLAISIGTSHGAFKFKPGE-----------
Pelodictyon_phaeoclathratiform  IELTKKVVAYAHQFD---VTVEGELGVLAGIEDEVS------------AAHHTYTQPEEVEDFVSKTGVDSLAISIGTSHGAFKFKPGE-----------
Chlorobium_chlorochromatii      VALTKQVVEYAHQYD---VTVEGELGVLAGIEDEVS------------ATHHTYTQPEEVEDFVAKTGVDSLAISIGTSHGAFKFKPGE-----------
Prosthecochloris_aestuarii      VTLTRQVVDYAHQYD---VTVEGELGVLAGVEDEVA------------SETHTYTQPEEVEDFVTRTGVDSLAIAIGTSHGAFKFKPGE-----------
Chlorobaculum_parvum            VKLTRKVVEFAHQHD---VTVEGELGVLAGIEDDVH------------AAEHTYTEPDQVEDFVGKTGVDSLAIAIGTSHGAFKFKPGE-----------
Victivallis_vadensis            IKETRRVVEYAHQFG---VTVEGELGVLAGVEDDVK------------AESHTYTQPEEVEDFVKRTGVDSLAIAIGTSHGAFKFKPGE-----------
Brachyspira_murdochii           VELTRSVVEYAHKYD---VTVEGELGVLAGVEDDVS------------AEHHTYTQPEEVEDFVKKTGVDSLAISIGTSHGAYKFKPGQ-----------
Brachyspira_pilosicoli          VELTRSVVEYAHKYD---VTVEGELGVLAGVEDDVV------------AESHTYTRPEEVEDFVKKTGVDSLAISIGTSHGAYKFKPGQ-----------
Alistipes_indistinctus          VALTKKVVDYAHQFD---VTVEGELGVLAGVEDEVS------------SDHHTYTRPEEVVDFVTKTGCDSLAISIGTSHGANKFKPEQCTRD-ANGVLV
Tannerella_sp                   VALTKKVVEYAHQFD---VTVEGELGVLAGVEDEVS------------AEHHTYTKPEEVVDFVTKTGCDSLAISIGTSHGANKFKPEQCTRN-ADGILV
Tannerella_forsythia            VALTKQVVEYAHQHD---VTVEGELGVLAGVEDDVA------------AEHHTYTRPEEVVDFVTKTGCDSLAISIGTSHGANKFKPEQCKRD-ANGRLV
Bacteroides_intestinalis        VALTKKVVDYAHQFD---VTVEGELGVLAGVEDEVS------------AEHHTYTDPEEVIDFATRTGCDSLAISIGTSHGAYKFKPEQCHVDPKTGRLV
Bacteroides_uniformis           VALTKKVVDYAHQFD---VTVEGELGVLAGVEDEVS------------AEHHTYTNPEEVIDFATRTGCDSLAISIGTSHGAYKFKPEQCHVDPATGRLV
Parabacteroides_merdae          VALTKKVVDYAHQFD---VTVEGELGVLAGVEDEVS------------SDHHTYTDPEEVIDFAHRTGCDSLAISIGTSHGAYKFTPEQCHIDPKTGKMV
Parabacteroides_sp              IALTKKVVEYAHQFD---VTVEGELGVLAGVEDEVS------------SDHHTYTNPEEVIDFATRTGCDSLAISIGTSHGAYKFTPEQCTIDPVTGKMV
Capnocytophaga_sp               VALTKKVVEYAHQFD---VTVEGELGVLAGVEDDVV------------AEHHTYTRPEEVVDFVTKTGCDSLAISIGTSHGAYKFKPEQCHVDPATGRLV
Paraprevotella_clara            VALTKKVVEYAHQFD---VTVEGELGVLAGVEDDVV------------AEHHTYTRPEEVVDFVTKTGCDSLAISIGTSHGAYKFKPEQCHVDPATGRLV
Paraprevotella_xylaniphila      VALTKKVVEYAHQFD---VTVEGELGVLAGVEDDVV------------AEHHTYTRPEEVVDFVTKTGCDSLAISIGTSHGAYKFKPEQCHVDPATGRLV
Bacteroides_salanitronis        VALTKKVVEYAHQYD---VTVEGELGVLAGVEDEVS------------AEHHTYTNPEEVIDFATRTGCDSLAISIGTSHGAYKFKPEQCHVDPKTGRLV
Dysgonomonas_gadei              VALTKKVVEYAHQFD---VTVEGELGVLAGVEDEVS------------AEHHTYTKPEEVVDFVTKTGCDSLAISIGTSHGANKFTPEQCTRD-ANGHLV
Dysgonomonas_mossii             IALTKKVVEYAHQFG---VTVEGELGVLAGVEDEVV------------AEHHTYTRPEEVVDFATRTGCDSLAISIGTSHGANKFTPEQCTRD-EKGHLV
Paludibacter_propionicigenes    IALTKKVVEYAHAHG---VSVEGELGVLAGVEDDVV------------AEHHTYTRPEEVVDFVTRTGVDSLAISIGTSHGANKFTPAQCTRD-ENGILI
Alistipes_sp                    VALTKQVVDYAHQFD---VTVEGELGVLAGVEDEVS------------AEHHTYTDPKDVVDFVSKTGVDSLAISIGTSHGANKFKPEQCTRN-AEGILV
Odoribacter_laneus              VALTRKVVDYAHQFD---VTVEGELGVLAGVEDEVS------------AEHHTYTRPEEVEDFVSKTGVDSLAISIGTSHGANKFKPEQCTRN-ADGVLV
Porphyromonas_endodontalis      VALTRKVVDYAHQFD---VTVEGELGVLAGIEDDVQ------------AETHTYTDPAQVEDFVKKTGVDSLAISIGTSHGAYKFTPAQCTRN-EEGVLV
Candidatus_Azobacteroides       VILTRKVVEYAHRYD---ISVEGELGVLAGIEDDVV------------AEHHTYTQPEEVEDFVSKTGVDSLAISIGTSHGANKFKPEQCIRS-AEGVLI
Sphaerochaeta_pleomorpha        VALTKKVVEYAHQFG---VTVEGELGVLAGIEDEVS------------SAVSHYTQPSEVIDFVSKTGVDSLAISIGTSHGANKFTPEQCTRN-AEGILI
Sphaerochaeta_coccoides         IAVTKKVVEYAHKFD---VTVEGELGVLAGIEDDVV------------AETSHYTKPEEVIDFVSRTGVDSLAISIGTSHGANKFTPEQCTRN-ADGVLI
Spirochaeta_caldaria            VALTKKVCEFAHSRKDY-VSVEGELGVLAGVEDDVS------------AEHSHYTQPEEVEDFVRKTGVDSLAISIGTSHGRTKFKPEQCTRD-ANGILI
```

```
Treponema_saccharophilum          VALTKKVCDYAHEHD---VTVEGELGVLGGVEDDVA------------AEESKYTKPEEVQDFVSKTGVDSLAISIGTSHGRCKFTPAQCTRT-ADGILV
Treponema_sp                      VALTKKVVEYAHAHD---VTVEAELGVLGGVEDDVA------------AEESKYTKPEEVIDFTSKTGCDSLAISIGTSHGRCKFTPEQCTRT-ADGVLI
Melioribacter_roseus              VELTAQVVEYAHKYD---VTVEGELGVLAGIEDDVQ------------SDVTHYTKPEEVEDFVKKTGVDSLAISIGTSHGANKFKPEQCTRN-EDGILI
Spirochaeta_thermophila           VELTRKVVEYAHKYD---VTVEGELGVLAGIEEHVS------------SEKTIYTDPNQVEDFVKRTGVDSLAIAIGTSHGAVKFKPEQCTRL-PDGRLV
Prevotella_multisaccharivorax     IELTRKCVEYAHKYD---VTVEAELGVLAGVEDEVA------------SEESHYTKPEEVIDFSKRSGCDSLAISIGTSHGAYKFKPEQCTRDPKTGRLI
Prevotella_disiens                VALTKKVVEYAHQFD---VTVEGELGVLAGVEDDVV------------AEESHYTRPEEVVDFAKRTGCDSLAISIGTSHGAYKFTPEQCTRDPKTGKLV
Fibrobacter_succinogenes          IALTKKVVEYAHAHD---VTVEAELGVLAGVEDEVA------------SEVSHYTKPEEVIDFATRTGCDSLAISIGTSHGAYKFKPEQCTRN-AQGKLV
Elusimicrobium_minutum            IALTKKVVEYAHAND---VTVEAELGVLAGIEDEVS------------AEHHTYTDPAQVEDFVKRTGCDSLAISIGTSHGAYKFKVKPG--------ES
Hippea_maritima                   VEITAKVVEYAHKYD---VSVEGELGVLAGIEEDVK------------AEKSIYTNPDDVEDFVKSTGVDSLAIAIGTSHGAYKFKVKPG--------EQ
Deferribacter_desulfuricans       IEITRKVVEYAHQFD---VTVEGELGVLAGIEEDVK------------AEKSHYTNPDEVEEFVERTGVDSLAISIGTSHGAYKFKVKPG--------EQ
Calditerrivibrio_nitroreducens    IAVTRKVVEYAHQFD---VTVEGELGVLAGIEDDVS------------AEHSHYTDPAQVEEFVAKTGVDSLAISIGTSHGAYKFKVKPG--------EE
Flexistipes_sinusarabici          VELTKRVVDYAHQYE---VTVEGELGVLAGIEDDVS------------AEKSHYTNPEEVEDFVNRTGVDSLAISIGTSHGAYKFKVAPG--------ES
Pelobacter_carbinolicus           VALTRRVVEYAHAHD---VTVEGELGVLAGIEDEVV------------AEHSTYTKPEEVEDFVSKTGVDSLAISIGTSHGAYKFKVAPG--------EE
Geobacter_lovleyi                 VALCKKVVEYAHQFD---VSVEGELGVLAGIEDEVS------------AEHSTYTKPEEVEDFVKKTGVDSLAISIGTSHGAYKFKAG----------QP
Desulfurispirillum_indicum        MALTKKVVEYAHQFD---VSVEGELGVLAGIEDEVS------------SEVSHYTDPEEVEEFVRFTGVDSLAISIGTSHGAFKFAPG----------KP
Trichomonas_vaginalis             VALTKKVVEYAHSRPDY-VTVEGELGVLAGVEDDVK------------AECHTYTRPEEVQDFVTKTGVDSLAIAIGTSHGAYKFPPGT-----------
Entamoeba_dispar                  VKLTKQVVEYAHQFG---VTVEGELGVLSGVEDDVA------------AAEHVFTDPKDVEAFVKDTGVDALAISIGTSHGAYKFKPG-----------M
Entamoeba_histolytica             VKLTKQVVEYAHKFG---VTVEGELGVLSGVEDDVA------------AAEHVFTDPKDVEAFVKDTGVDALAISIGTSHGAYKFKPG-----------M
Leptotrichia_goodfellowii         VKESKEVADFAHKHD---VTVEAELGVLAGVEDDVV------------AEKHIYTQPDEVEDFVKKTGVDSLAIAIGTSHGAHKFKPGD-----------
Leptotrichia_hofstadii            VAEAKQVADFAHQHD---VTVEAELGVLAGIEDDVV------------AAEHVYTQPDEVEDFVTKTGVDSLAIAIGTSHGAHKFKPGD-----------
Streptobacillus_moniliformis      IELSKKVAEFAHQND---VTVEAELGVLAGVEDDVV------------AEETIYTQPDEVEEFVNRTGVDSLAIAIGTSHGAHKFKPGD-----------
Fusobacterium_mortiferum          IEVSKEVVEFAHAKD---VTVEAELGVLAGIEDDVK------------AESHTYTNPDEVEEFVTKTGVDSLAIAIGTSHGAHKFKPGE-----------
Fusobacterium_gonidiaformans      IKVTKEVVEYAHKNAGEYVSVEAELGVLAGIEDDVH------------AEEHKYTNPEEVIDFVGRTGVDSLAIAIGTSHGAHKFKPGE-----------
Alkaliphilus_metalliredigens      IEITKKVVDYAHSKG---VVVEAELGRLAGVEDDVNVS----------AEDASYTNPEQAVEFVKRTGVDSLAIAIGTSHGAYKFKGE------------
Veillonella_sp                    VRITKQVVEYAHAHG---VVVEAELGRLAGVEDDVNVS----------DADARFTDPEQAAEFVHLTGVDSLAIAIGTSHGAYKFKGD------------
Acetonema_longum                  ITVTKKVVEYAHARG---VTVEAELGRLAGVEDAVKVS----------SKDASYTDPDQAVEFVERTGVDSLAIAIGTSHGAYKFKGE------------
Thermosinus_carboxydivorans       IALTKKVVDYAHERG---VTVEGELGRLAGVEDAVKVS----------EREATYTDPDQAVEFVERTGVDSLAIAIGTSHGAYKFKGE------------
Pelosinus_fermentans              IALTKKVVEYAHAHG---VVVEAELGKLAGVEDAVKVS----------AKDATYTDPDEAVEFVERTGCDSLAIAIGTSHGAYKFKGE------------
Desulfosporosinus_sp              IAVTKKVVEYAHAKG---VVVEAELGRLAGIEDAVNVR----------AKDSSYTDPEQAGEFVHQTGCDSLAIAIGTSHGAYKFKGE------------
Desulfosporosinus_acidiphilus     IAVTKKVVEYAHAKG---VVVEAELGRLAGVEDAINVS----------AEDSSYTDPEQAAEFVQKTGCDSLAVAIGTSHGAYKFKGE------------
Acidaminococcus_sp                IELTKRVVDYAHSKG---VVVEAELGKLAGVEDAVKVN----------TKDATYTDPDQAVEFVERTGVDSLAIAIGTSHGAYKFKGK------------
Acidaminococcus_intestini         IELTKRVVDYAHSKG---VVVEAELGKLAGVEDAVKVN----------TKDATYTDPDQAVEFVERTGVDSLAIAIGTSHGAYKFKGK------------
Phascolarctobacterium_succinat    IEMTRRVVEYAHERG---VVVEAELGRLAGVEDAVKVN----------TKDATYTDPDQAVEFVERTGCDSLAIAIGTSHGAYKFKGK------------
Caldicellulosiruptor_kristjans    IALTKKVVEYAHERG---VVVEAELGKLAGIEDNVKVA----------EHEAAFTDPDQAAEFVERTGVDSLAVAIGTSHGAYKFKGD------------
Caldicellulosiruptor_lactoacet    IALTKKVVEYAHERG---VVVEAELGKLAGIEDNVKVA----------EHEAAFTDPDQAAEFVERTGVDSLAVAIGTSHGAYKFKGD------------
Tepidanaerobacter_acetatoxydan    IELTKRVVDYAHERG---VVVEAELGRLAGIEDAVKVS----------EQDALFTDPDEAVEFVERTGVDSLAIAIGTSHGAYKFKGE------------
Thermosediminibacter_oceani       IALTRRVVEYAHERG---VVVEGELGRLAGVEDNVKVS----------DREATFTDPDQALEFVERTGVDSLAVAIGTSHGAYKFKGE------------
Caloramator_australicus           IKITKQVVEYAHSKG---VVVEAELGKLAGVEDEVKVS----------AREATFTDPEEAYEFVHKTGVDSLAVAIGTSHGAYKFKGE------------
Eubacterium_saphenum              VALTKQVVDYAHERG---VVVEAELGKLAGIEDNINVD----------AREATFTDPEQAKEFVERTGVDSLAVAIGTSHGAYKFKGE------------
Eubacterium_infirmum              IALTKQVVEYAHAHG---VVVEAELGKLAGVEDNIKVD----------ARSATFTDPDEAVEFVERTGVDSLAVAIGTSHGAYKFKGE------------
Acetobacterium_woodii             IALTKKVVDYAHDHG---VVVEAELGKLAGIEDDVNVD----------AHNATFTDPDEAVEFVERSGCDSLAITIGTSHGAFKFKGE------------
```

```
Flavonifractor_plautii          IEITRKVVEYAHDHG---VVVEAELGALAGIEDDVQVS----------AEESHYTHPEEVEEFVSKTGCDSLAIAIGTSHGAYKFTADQCTRN-EKGELV
Oscillibacter_valericigenes     IEITKKVVEYAHDHG---VVVEAELGTLAGIEDDVKVK----------SEDSSYTHPEEVEEFVSKSGCDSLAIAIGTSHGAYKFKPG-----------T
Bryantella_formatexigens        IALTKQVVEYAHAHG---VVVEAELGTLAGVEDEVCVE----------AGHESYTRPEEVEEFVDKTGCDSLAIAIGTSHGAYKFKASQCTRN-ADGVLV
Stomatobaculum_longum           IAETKKVVEYAHAHG---VVVEAELGTLAGVEDDVRVE----------EGMSSYTRPEEVEEFVSRTGCDSLAIAIGTSHGAYKFKPG-----------T
Selenomonas_sputigena           IELTKRVVEYAHAHG---VVVEGELGQLAGIEDDVKVA----------AEDAHYTRPEEVEEFVSSTGVDSLAIAIGTSHGAFKFKPEQCTKN-AQGVLV
Megasphaera_elsdenii            IALTKKVVEYAHAHG---VVVEGELGQLAGIEDDVKVA----------AHEAHYTRPEEVEEFVSRTGVDSLAIAIGTSHGAFKFTPEQCTRN-ADGVLV
Mitsuokella_multacida           IKRTQEVVAYAHEHG---VVVEAELGKLAGIEDDVKVA----------AHEAAYTQPEEVEEFVRETGVDSLAIAIGTSHGAFKFKPEQCTRN-ADGVLV
Selenomonas_infelix             VALAKRVAEYAHAHG---VVVEAELGQLAGIEDDVNVS----------AEDASYTKPEEVQDFVEKTGVDSLAIAIGTSHGAFKFTPDQCTRN-ADGVLV
Centipeda_periodontii           VALAKRVAEYAHAHG---VVVEAELGQLAGIEDDVNVS----------AENASYTKPEEVQDFVEKTGVDSLAIAIGTSHGAFKFTPDQCTRN-ADGVLV
Megamonas_funiformis            IEKTKEVVEYAHAHG---VVVEAELGQLAGIEDDVKVA----------AHEAHYTDPNEVEEFVKETGVDSLAIAIGTSHGAYKFTPDQCTRN-EQGILV
Anaeroglobus_geminatus          AELAKRVADYAHERG---VVVEAELGQLAGIEDDVKVD----------AQQASYTRPEEVEEFVTRTGVDSLAIAIGTSHGAYKFRPEQCTKN-EQGVYV
Clostridium_thermocellum        IKLTKQVVDYAHSKG---VVVEGELGRLAGIEDDVNVS----------EADAAFTDPDQAEEFVKRTGVDSLAIAIGTSHGAYKFKGE------------
Acetivibrio_cellulolyticus      IKLTKQVVEYAHDKG---VTVEGELGRLAGIEDAVNVS----------DADAAFTDPDQVQEFVEKTGVDSLAIAIGTSHGAFKFKGE------------
Dictyoglomus_turgidum           VRITKEVVDYAHPRG---VAVEGELGRLVGVEEHVVVS----------EREASFTDPDKAVEFVERTGVDSLAIAIGTSHGAYKFKGE------------
Dictyoglomus_thermophilum       VRITKEVVDYAHPRG---VAVEGELGRLVGVEEHVVVS----------EREATYTDPDKAVEFVERTGVDSLAIAIGTSHGAYKFKGE------------
Thermotoga_lettingae            VKLTKQVVDYAHPRG---VVVEGELGRLVGVEEHVVVS----------EKEASYTDPDKAVEFVERTGVDSLAIAIGTSHGAYKFKGE------------
Thermotoga_thermarum            VKLTRQVVEYAHPRG---VVVEGELGRLVGVEEHVVVD----------EREAVFTDPDKAVEFVERTGVDSLAISIGTSHGAYKFKGE------------
Caldisericum_exile              VALTKKVVEYAHSKN---VVVEGELGKLQGIEEHVVSN------------EAVYTDPDKAVEFVERTGVDSLAIAIGTSHGAYKFKGE------------
Bilophila_sp                    IAVTKRVVEYAHAHG---VWVEAELGRLAGVEEDVS------------SEHSIYTDPDQAVEFVERSGCDSLAIAIGTSHGAYKFKGE------------
Bilophila_wadsworthia           IAVTKRVVEYAHAHG---VWVEAELGRLAGVEEDVS------------SEHSIYTDPDQAVEFVERSGCDSLAIAIGTSHGAYKFKGE------------
Lawsonia_intracellularis        IALTKQVVDYAHAHG---VWVEAELGRLAGVEDDVS------------SEHSVYTDPDQAVEFVQRSGCDSLAIAIGTSHGAYKFKGK------------
Desulfohalobium_retbaense       IALTKEVVEYAHPRG---VWVEAELGQLAGVEDDVQ------------AEENVYTNPDEAVEFVERTGCDSLAIAIGTSHGAYKFSGQ------------
Desulfomicrobium_baculatum      IALTKQVVAYAHDRG---VWVEAELGQLAGVEDDVD------------VEHSVYTNPDQAAEFVGRTGCDSLAIAIGTSHGAYKFAGE------------
Desulfovibrio_alaskensis        IAVTRRVVEYAHDKG---VWVEAELGQLAGVEEDVS------------AEHSVYTDPDQAVEFVERTGCDSLAIAIGTSHGAYKFTGK------------
Desulfonatronospira_thiodismut  IALTKQVVECAHGQG---VWVEAELGRLAGVEDEVS------------SAENVFTDPDEAVEFVERTGCDSLAIAIGTSHGAYKFKGE------------
Desulfovibrio_piger             IALTKQVVEYAHPRG---IWVEAELGKLAGVEEHVS------------NADHVYTDPDQAVDFVERTGCDSLAVAIGTSHGAYKFKGE------------
Dialister_succinatiphilus       VKVTKQIVEYAHDKG---VVVEAELGRLAGVEDMVSVD----------AKDAIFTDPDQAAEFVERTGCDSLAIAIGTSHGAYKYKGD------------
Dialister_micraerophilus        IKITKQVVEYAHAHG---VVVEAELGRLAGVEDLVSVD----------AKDAIFTDPDQAVEFVERTGCDSLAIAIGTSHGAYKYSGE------------
Clostridium_botulinum           VAKTKEVVEYAHSHG---VVVEAELGVLAGVEDDVQ------------SDVHIYTDPEQAVDFVNRTGVDSLAIAIGTSHGAYKFAG-------------
Peptostreptococcus_anaerobius   VAITKQVVEYAHQHN---VSVEAELGVLAGVEDEVS------------SDVHKYTEPDQAVDFVNRTGVDSLAIAIGTSHGAFKFKG-------------
Arthromitus_sp                  VKLTKEVVEYAHSKG---VCVEAELGVLAGVEDDVV------------ADSHIYTNPDEAVEFVKQTNCDSLAIAIGTSHGAFKFPKD-----------F
Leptonema_illini                IRLTRRVVDYADERG---VFVEAELGQLAGVEDDVS------------AEHSVFTDPDQAVEFVHRTGCHSLAVAIGTSHGAYKFKGA------------
Anaerofustis_stercorihominis    IEITKQVVEYAHDHG---VTVEAELGKLAGVEDAVKVA----------AKDATYTDPEEAAEFVEKTGVDSLAIAIGTSHGAYKFSGE------------
Methanocella_arvoryzae          IAMTKKIVEFAHAAG---VSVEAELGKLAGVEGHVSVS----------EKDAIFTNPEEAKIFVEQTGVDALAVAIGTSHGAFKFKGE------------
Methanocella_conradii           IRQTAEIVRIAHAAG---VSVEAELGRLAGVEGHVSVA----------ERDAIFTNPDEAKLFVERTGVDALAVAIGTSHGAYKFKGE------------
Staphylococcus_lugdunensis      IEITSKVVEYAHQHG---VSVEAELGTVGGQEDDVVG-------------GIIYADPKECQELVEKTGIDALAPALGSVHGPYK--GE------------
Staphylococcus_aureus           VATTKKVVEYAHEKG---VSVEAELGTVGGQEDDVVAD------------GIIYADPKECQELVEKTGIDALAPALGSVHGPYK--GE------------
Listeria_grayi                  VAMTKKVVDYAHAKG---VSVEAEVGTVGGTEDGVTG-------------GINYADPNECLRVVKEAEVDALAAALGSVHGPYS--GE------------
Brevibacillus_brevis            LRLTKQVVEAAHAVG---VSVEGELGTIGGVEDDLSVD----------EEDATLANPEEAIRFWEETKVDYVAIAVGTAHGMYK--GV------------
Kyrpidia_tusciae                IRLTKQVVDACKVLG---VSVEGELGRIGGTEDELTVD----------EREASLVRPEEAKRLVEETGIDALAPAIGSAHGRYK--GK------------
Halanaerobium_praevalens        IALTKKVVEAAHNVG---VSVEAELGTIGGTEDDHTVA----------EKDAMYTDPDQAKEFIEATGVDALAIAIGTAHGVYE--GE------------
```

```
Halanaerobium_hydrogeniformans  IALTKKVVEAAHSVG---VSVEAELGTIGGTEDDHTVD----------EKDAMYTDPDQALEFVERTGVDALAIAIGTAHGVYA--GE------------
Halothermothrix_orenii          VRLVKKVVEAAHSVG---VSVEAELGKIGGTEDNVSVD----------EKDATYTDPDEAVEFVERTGVDALAIAIGTAHGVYK--GK------------
Thermoanaerobacter_wiegelii     VELTKKVVEVAHAAG---VTVEAELGNIVGVEDDMYVK----------EDKSAFTDPKMAVDFVEKTGVDSLAIAIGTAHGMYK--GE------------
Helicobacter_pylori             LELTSKVVKMAHNAG---VSVEAELGRLMGIEDNISVD----------EKDAVLVNPKEAEQFVKESQVDYLAPAIGTSHGAFKFKGE------------
Helicobacter_acinonychis        LELTSKVVKMAHNAG---VSVEAELGRLMGIEDNISVD----------EKDAVLVNPKEAEQFVKESQVDYLAPAIGTSHGAFKFKGE------------
Thiovulum_sp                    LAKTKAVVDIAHAVG---VGVEAELGRLMGIEDNIVVS----------ERDAVLVNVAEAEKFVKESGVDYLAPAIGTSHGAFKFKGE------------
Fervidobacterium_nodosum        MKKTKEIVKIAHAAG---VSVEAELGQLAGIEDNVSAA------------ENVLVDPEQAKIFVEETGVDFLAPAIGTSHGAFKFKGE------------
Thermosipho_melanesiensis       LEITKEVVKWAHAAG---VSVEAELGQLAGIEDNVVAK------------ENVLVDPQQAKIFVEETNVDFLAPAIGTSHGAFKFKGE------------
Marinitoga_piezophila           MKRTKEIVRIAHAAG---VSVEAELGQLAGVEDEAVAA------------ENVLVNPEEAKIFVEETGVDFLAPAVGTSHGAFKFKGE------------
Mesotoga_prima                  VAETKKIVEIAHSLG---VSVEAELGRLVGIEDNVAVE----------SHEAALVDPDEAKLFAEETGVDFLAPAIGTSHGAFKFKGE------------
Aquifex_aeolicus                LKVTKYVVSICAALG---ISVEAELGKLVSVEDEVVSR------------EEVLVDPEQAKEFVDRTGIDALAPAIGTAHGAFKFKGE------------
Truepera_radiovictrix           TRETKRVVEAAHAVG---VTVEAEIGRLGGIEEHVVVS----------EEEAILTKPDEAERFMQATGADYLAVAIGTSHGANKGKGR------------
Thermus_thermophilus            VRETKRVVEAAHAVG---VTVEAELGRLAGIEEHVAVD----------EKDALLTNPEEARIFMERTGADYLAVAIGTSHGAYKGKGR------------
Meiothermus_silvanus            IRETKRVVEAAHAVG---VTVEAEIGRLGGVEEHVAVS----------AEEAFLTNPEEAKMFMDATGADYLAVAIGTSHGAYKGKGR------------
Deinococcus_deserti             VHETRRVVEAAHAMG---ISVEAEIGRLGGIEEHIVVD----------EKDAFLTDPEEAVQFVEQTGTDYLAIAIGTSHGAYKGKGR------------
Meiothermus_ruber               VRETKKCVEAAHAVG---VSVEAELGRLQGIEDNIVVD----------AKEAFLTDPDEAARFVEATGIDYLAVAIGTSHGAYKGKGR------------
Thermus_scotoductus             VAETKKVVEAAHAVG---VSVEAELGRLQGIEDNIQVS----------EAEAFLTDPDEAERFVAETGIDYLAIAIGTSHGAYKGKGR------------
Giardia_lamblia                 VRITKEVVAYAHARG---VSVEAELGTLGGIEED------------VQN-TVQLTEPQDAKKFVELTGVDALAVAIGTSHGAYKFKSESDI---------
Bacterium_phylotype_RsD17       IKVTRSVVEYAHARG---VSVEAEIGTLGGIEDG------------VGSGKIHLTDPKEAERFVNETDVDSLAIAIGTSHGAYKFKGAAN----------
Eikenella_corrodens             VDVTRTVVNFSHACG---VSVEGEIGVLGNLETGEAGEEDGVGAVGKLSHDQMLTSVEDAVRFVNDTGVDALAIAVGTSHGAYKFTRPPTG---------
Kingella_kingae                 VEATRKVVQFSHACG---VSVEGEIGVLGNLETGEAGEEDGVGAEGKLSHDQMLTSVEDARRFVQDTGVDALAIAIGTSHGAYKFTRPPTG---------
Neisseria_shayeganii            VDVTRTVVNFAHACG---VSVEGEIGCLGNLETGDAGEEDGIGADVKLSHDQMLTSVEEAASFVKDTGVDALAIAIGTSHGAYKFSRKPTG---------
Laribacter_hongkongensis        VDVTRTVVNFAHACG---VSVEGEIGCLGSLETGKAGEEDGVGAEGVLDHSQLLTDPEEAARFVKDTGVDALAIAIGTSHGAYKFTKKPTG---------
Acinetobacter_baumannii         VDVTRQVVAMAHACG---VSVEGEIGCLGSLETGMAGEEDGVGAEGVLDHSQLLTSAEEAKQFVADTNVDALAIAVGTSHGAYKFTRPPTG---------
Acinetobacter_sp                VDVTRRVVAMAHACG---VSVEGEIGCLGSLETGLAGEEDGVGAEGVLDHSQLLTSVEEATQFVADTNVDALAIAVGTSHGAYKFTRPPTG---------
Marinomonas_mediterranea        IDVTRRTVEFAHACG---VSVEGELGCLGSLETGQAGEEDGVGAEGILSHAQMLTDPDEAKEFVEATGVDALAVAIGTSHGVYKFTRPPTG---------
Oceanospirillum_sp              VDVTRRTVEMAHACG---VSVEGELGCLGSLETGQAGEEDGIGAEGTLSHDQMLTDPDEAADFVKKTGVDALAIACGTSHGAYKFTRPPTG---------
Marinobacterium_stanieri        VDVTRRTVEMAHACG---VSVEGELGCLGSLETGQAGEEDGIGAEGTLTHDQMLTDPDEAADFVTQTNVDALAIACGTSHGAYKFTKPPTG---------
Oceanobacter_sp                 VDVTKRTVDMAHACG---VSVEGELGCLGSLETGEAGEEDGIGAVGKLTHDQMLTNPEEAADFVKQTGVDALAIACGTSHGAYKFTRPPTG---------
Colwellia_psychrerythraea       VDVTRRTVEMAHACG---VSVEGELGCLGSLETGMAGEEDGVGAEGVLTKEQMLTDPEEAADFVNKTQVDALAIACGTSHGAYKFTRPPTG---------
Hahella_chejuensis              VDVTRRTVEMAHACG---VSVEGELGCLGSLETGTAGEEDGIGAEGVLSHDQMLTDPDEAADFVKKTQVDALAIAIGTSHGAYKFSRPPTG---------
Alishewanella_aestuarii         VEVTRRVVEIAHACG---VSVEGELGCLGSLETGEAGEEDGIGADCKLSHDQMLTDPEEAAQFVRETQVDALAIACGTSHGAYKFSRPPTD---------
Alishewanella_jeotgali          VEVTRRVVEIAHACG---VSVEGELGCLGSLETGEAGEEDGIGADCKLSHDQMLTDPEEAAQFVRETQVDALAIACGTSHGAYKFSRPPTD---------
Rheinheimera_nanhaiensis        ADVTRKVVEIAHACG---VSVEGELGCLGSLETGAAGEEDGIGADCILSHDQMLTDPEEAAQFVRETQVDALAIACGTSHGAYKFSRPPTG---------
Congregibacter_litoralis        VDVTRTAVAMAHACG---VSVEGELGCLGSLETGEAGEEDGSGAAGKLTHDQLLTDPEEAAAFVKATEVDALAIACGTSHGAYKFTRPPTG---------
Pseudoalteromonas_marina        VEVTRETVAMAHACG---VSVEGELGVLGSLETGEAGEEDGVGAEGKLTTDQMLTDPEEAADFVNKTHVDALAIACGTSHGAYKFTRPPTD---------
Alteromonadales_bacterium       VEVTRETVAMAHACG---VSVEGELG-VGSLETGEAGEEDGVGAEGKLTTDQMLTDPEEAADFVNKTHVDALAIACGTSHGAYKFTRPPTD---------
Idiomarina_baltica              VRVTKQTVEMAHACG---VSVEGELGCLGSLETGEAGEEDGVGAEGKLSHDQLLTDPEEAAAFVAATDVDALAIACGTSHGAYKFTRPPTG---------
Shewanella_baltica              VDVTRRTVAFAHACG---VSVEGEIGCLGSLETGQAGEEDGVGASGILSHDQMLTSPEEAARFVADTHVDALAIAIGTSHGAYKFSRKPTG---------
Shewanella_violacea             VGVTRRTVAFAHACG---VSVEGEIGCLGSLETGEAGEEDGIGAAGILTMDQMLTTPEEAARFVSDTHVDALAIAIGTSHGAYKFSRKPTG---------
Ferrimonas_balearica            VQVTQRTVAFAHACG---VSVEGEIGCLGSLETGMAGEEDGVGAEGVLSHDQLLTDPEEAARFVAATKVDALAIAIGTSHGAYKFSKKPTG---------
```

```
Pseudomonas_mendocina               VRVTQQTVAFAHACG---VSVEGELGCLGSLETGMAGEEDGVGAEGVLDHSQLLTDPEEAADFVKKTQVDALAIAIGTSHGAYKFTKPPTG---------
Pseudomonas_fulva                   VRVTQQTVAFAHACG---VSVEGELGCLGSLETGMAGEEDGVGAEGVLDHSQLLTDPEEAADFVKKTQVDALAIAIGTSHGAYKFTKPPTG---------
Azotobacter_vinelandii              VRVTRQTVAFAHACG---VSVEGELGCLGSLETGQAGEEDGVGAEGTLDHSQMLTDPEEAARFVKETQVDALAIAIGTSHGAYKFTKPPTG---------
Halomonas_sp                        VDVTRRTVEMAHACG---VSVEGELGCLGSLETGMAGEEDGIGAEGVLSHDQLLTDPEEAAAFVKATQVDALAIAIGTSHGAYKFTKPPTG---------
Halomonas_boliviensis               VNVTRRAVEMAHACG---VSVEGELGCLGSLETGMAGEEDGIGAEGKLDMEQLLTDPEEAAAFVKATHVDALAIAIGTSHGAYKFTKPPTG---------
Chromohalobacter_salexigens         VDVTRRAVEMAHACG---VSVEGELGCLGSLETGQAGEEDGVGAEGTLDHSQLLTDPEEAAQFVAATQVDALAIAIGTSHGAYKFTRPPTG---------
Marinobacter_algicola               VDVTRRTVEMAHACG---VSVEGELGCLGSLETGQAGEEDGIGAEGTLDHSQMLTDPEEAADFVKKTHVDALAIAIGTSHGAYKFTRPPTG---------
Marinobacter_aquaeolei              VDVTRRTVEMAHACG---VSVEGELGCLGSLETGQAGEEDGIGAEGTLDHSQMLTDPEEAADFVQKTQVDALAIAIGTSHGAYKFTRPPTG---------
Thiocystis_violascens               VDVTRSVVAISHACG---VSVEGELGCLGSLETGQAGEEDGIGAEGTLDHSQLLTDPEEAADFVKKTGVDALAIAIGTSHGAYKFTRPPTG---------
Thiocapsa_marina                    VEVTRRVVEMAHACG---VSVEGELGCLGSLETGQAGEEDGIGAEGILDHSQLLTDPEEAADFVKKTSVDALAIAIGTSHGAYKFTRPPTG---------
Allochromatium_vinosum              IEVTRRVVEMAHACG---VSVEGELGCLGSLETGQAGEEDGIGAEGTLDHSQLLTDPNEAADFVKKTGVDALAIAIGTSHGAYKFTRPPTG---------
Thiorhodococcus_drewsii             VDITRQVVEIAHACG---VSVEGELGCLGSLETGQAGEEDGIGAEGTLNHSQLLTDPEEAADFVKKTAVDALAIAIGTSHGAYKFTRPPTG---------
Marichromatium_purpuratum           VDVTRRVVEIAHACG---VSVEGELGCLGSLETGTAGEEDGIGAEGTLDHSQLLTDPEEAADFVAKTKVDALAIAIGTSHGAYKFTRPPTG---------
Thiomonas_intermedia                VDVTRRVVEMSHSCG---VSVEGELGCLGSLETGTAGEEDGIGAEGTLDHSQLLTDPEEAADFVKKTQVDALAIAIGTSHGAYKFTRQPTG---------
Francisella_novicida                VNVTKTVSDMAHACG---VSVEGELGCLGSLETGQAGEEDGIGAEGTLSMDQLLTDPEEAADFVRRTKVDALAIAIGTSHGAYKFTKPPTG---------
Francisella_noatunensis             VNVTKTVSDIAHACG---VSVEGELGCLGSLETGQAGEEDGVGAEGTLFMDQMLTDPEEAADFVKKTKVDALAIAIGTSHGAYKFTKPPTG---------
Kangiella_koreensis                 VRVTKLATEMAHACG---VSVEGELGCLGSLETGMAGEEDGSGAEGVLSHDQLLTDPEEAAQFVKDTKVDALAIAIGTSHGAYKFTRPPTG---------
Coxiella_burnetii                   VHTTALTTQMAHACG---VSVEGELGCLGSLETSRAAEEDGSGAEGELSRDQMLTDPEQAADFVNKTKVDALAIAIGTSHGAYKFSRPPTG---------
Thiothrix_nivea                     VDTTRKVVELAHACG---VSVEGELGCLGSLETGMMGEEDGHGAEGALDHSQLLTDPEEAADFVKKTGVDALAIAIGTSHGAYKFTQKPTG---------
Thiobacillus_denitrificans          VETTRKVSELAHACG---VSVEGELGCLGSLETGKAGEEDGHGAEGTLDHSMLLTDPDEAADFVSKTRVDALAIAIGTSHGAYKFTQKPTG---------
Thioalkalimicrobium_cyclicum        AGITAEVVKIAHAGG---VSVEGELGCLGSLETGMMGEEDGHGSDEKLDHSALLTDPEEAAQFVRETNVDCLAVAVGTSHGAYKFTSKPSD---------
Halothiobacillus_neapolitanus       VEVTRKVAEMSHFVG---VSVEGELGCLGSLETGKMGEEDGHGAEGELDHSMLLTDPEEAAQFVKMTDVDALAIAIGTSHGAYKFTQKPTG---------
Nitrosococcus_watsonii              VNVTRQVVDMAHSGG---VSVEGELGCLGSLETGMAGEEDGSGAEGQLSHDQLLTNPEEAADFVKKTKVDALAIAIGTSHGAYKFTRPPTG---------
Halorhodospira_halophila            ASVTRRAAEMAHAGG---VSVEGEIGVLGSLETGEAGKEDGVGAEGKMDKDKLLTDPEEAAQFVRDTHVDALAIACGTSHGAYKFTRPPTG---------
Alkalilimnicola_ehrlichii           ARVTRTVSEIAHAGG---VSVEGELGCLGSLETGEAGEEDGSGAEGKLSKEMMLTDPEEAAQFVRETHVDALAIAIGTSHGAYKFTRPPTG---------
Rubrivivax_benzoatilyticus          VEVTCRVVEIARAVG---VSVEGELGCLGSLETGTAGEEDGVGAEGTLDHAQLLTDPEQAADFVARTGVDALAIAIGTSHGAYKFTRQPTG---------
Rubrivivax_gelatinosus              VEVTRRVVEIARAVG---VSVEGELGCLGSLETGTAGEEDGVGAEGTLDHAQLLTDPEQAADFVARTGVDALAIAIGTSHGAYKFTRQPTG---------
Hydrocarboniphaga_effusa            VKVTSKVAEMAHAVG---VSVEGELGCLGSLETGEAGEEDGVGAAGVLDHSQLLTDPGQAAEFVAATGVDALAIAIGTSHGAYKFTRPPTG---------
Dechloromonas_aromatica             VLVTRKVVEMAHAIG---VSVEGELGCLGSLETGEAGEEDGIGAAGKLDRSQMLTDPDEAADFVARTGVDALAIAIGTSHGAYKFTRPPTG---------
Oxalobacteraceae_bacterium          VDVTRNVVAMAHCIG---VSVEGELGCLGSLESGQAGEEDGSGATGILSHAQLLTDPEQAADFVARTGVDALAIAIGTSHGAYKFKRKPTG---------
Leptothrix_cholodnii                VRVTADVCRIAHAVG---VSVEGELGCLGSLETGDAGEEDGVGAVGQLSHDQMLTDPAEARDFVARTGVDALAIAIGTSHGAYKFTREPTG---------
Methylovorus_glucosetrophus         VEVTRRVVDIAHAVG---VSVEGELGVLGSLETGMAGEEDGVGAEGHLDHSQLLTDPEEAADFVRFTNVDALAIAIGTSHGAYKFSRPPSG---------
Methylobacillus_flagellatus         VEVTRQVVMAAHAVG---VSVEGELGVLGSLETGLAGEEDGVGAEGRLDRSQLLTSPEEAVDFVARTGVDALAVAIGTSHGAYKFKRPPTG---------
Methylotenera_versatilis            VDVTRKIVEIAHAIG---VSVEGELGVLGSLETGLAGEEDGSGAVGVLNHDQMLTDPEEAADFVRRTGVDALAIAIGTSHGAYKFSKPPTG---------
Nitrosospira_multiformis            VEVTGKVVEMAHAVG---VSVEGELGCLGSLESGMGEAEDGHGAEGKLSHDQLLTDPEQAADFVKQTGVDALAIAIGTSHGAYKFTRKPTG---------
Gallionella_capsiferriformans       VNISRRVAEMSHAVG---VSVEGELGCLGSLESGEGEKEDGHGAEGVLSHDQLLTDPEEAAEFVRQTGVDALAIAIGTSHGAYKFTRPPTG---------
Methylomirabilis_oxyfera            VATTAHIAEVAHAIG---VSVEGELGCLGSLETGTGEKEDSHGAEGTLSRDQLLTDPEQAAQFVKATKVDALAIAIGTSHGAYKFSKKPEG---------
Acidithiobacillus_caldus            VTVTAKVVEMAHAVG---VTVEGELGCLGSLETGMAGEEDGVGAEGCLSHDQLLTDPEEAAQFVKATKVDALAIAIGTSHGAYKFTQPPTG---------
Magnetospirillum_magneticum         VAVTKQVVDIAHACG---VSVEGELGCLGSLETGTAGKEDGVGAEGKLDHSQLLTDPDEAAEFVKLTQVDALAIAIGTSHGAYKFTRPPTG---------
Magnetospirillum_magnetotactic      VAVTKQVVDIAHACG---VSVEGELGCLGSLETGTAGKEDGVGAEGKLDHSQLLTDPDEAAEFVKATKVDALAIAIGTSHGAYKFTRPPTG---------
Phaeospirillum_molischianum         VRVTRQVVDIAHACG---VSVEGELGCLGSLETGTAGEEDGVGATGKLDHSQLLTDPNEAAEFVKQTQVDALAIAIGTSHGAYKFTRPPTG---------
```

```
Aromatoleum_aromaticum           VAVSREVVKFSHAIG---ITVEAELGCLGSLETGQAGEEDGLGAEGTLDHSMLLTDPDQAADFVRQTDCDALAIAIGTSHGAYKFSRKPTG---------
Thauera_sp                       VAVTREVVKFSHAIG---VSVEAELGCLGSLETGQAGEEDGVGAEGTLDHSQMLTDPDQAADFVKQTDCDALAIAIGTSHGAYKFTRKPTG---------
Azoarcus_sp                      VAVTREVVKFSHAIG---VTVEAELGCLGSLETGMAGEEDGIGAEGKLDHSALLTDPDQAADFVKQTDCDALAIAIGTSHGAYKFTRKPTG---------
Dechlorosoma_suillum             VAVTQEVVKFSHAIG---VSVEAELGVLGSLETMKADKEDGHGAEGHMTREDLLTDPDQAADFVARTNCDALAIAIGTSHGAYKFTKKPTG---------
Accumulibacter_phosphatis        VDVTRKVVEFAHAIG---VSVEGELGVLGSLETMRGDKEDGHGAEGTMTREQLLTDVEQAADFVKQTQCDALAIAIGTSHGAYKFTKKPTG---------
Herbaspirillum_sp                VEVSRKVVEFSHAIG---VTVEAELGVLGSLETMKGDKEDGHGADGTMTREQLLTDVDQAADFVKQTQCDALAIAIGTSHGAYKFSRKPTG---------
Herbaspirillum_seropedicae       VEVSRKVVEFSHAIG---VTVEAELGVLGSLETMKGDKEDGHGADGTMTREQLLTDVNQAADFVKATQCDALAIAIGTSHGAYKFSRKPTG---------
Collimonas_fungivorans           VEVSRKVVEFSHAIG---VTVEAELGVLGSLETMMGDKEDGHGADGKMTREQLLTDVSQAADFVKQTQCDALAIAIGTSHGAYKFSRKPTG---------
Herminiimonas_arsenicoxydans     VEVSRKVVEFSHAIG---VTVEAELGVLGSLETMMGDKEDGHGADGKMTREQLLTDVEQAADFVKRTQCDALAIAIGTSHGAYKFSRKPTG---------
Polynucleobacter_necessarius     VDVSKEVVKFSHSIG---VTVEAELGVLGSLETMQGDKEDGHGADGKMTREQLLTDVEQAADFVKATQCDALAIAIGTSHGAYKFSKKPTG---------
Janthinobacterium_sp             VEVSREVVKFSHAIG---VTVEAELGVLGSLETMKGDKEDGHGADGTMTREQLLTDVAQAADFVQRTQCDALAIAIGTSHGAYKFTRKPTG---------
Burkholderia_cenocepacia         VDVSRKVVEMAHSIG---VTVEAELGVLGSLETMKGDKEDGHGAEGTMTREQLLTDPEQAADFVKLTQCDALAIAIGTSHGAYKFSKKPTG---------
Oxalobacter_formigenes           VEVTKEVVKFSHAVG---VTVEAELGVLGSLETMKADKEDGIGAEGTMTREQLLTNPDQAVDFVAQTQCDALAIAIGTSHGAYKFTRKPTG---------
Ralstonia_eutropha               VDVTRKVVQLSHAIG---VTVEGELGCLGSLETGEAGEEDGIGAEGKLDHSMLLTDPEQAADFVKATQLDALAIAIGTSHGAYKFTRKPTG---------
Cupriavidus_taiwanensis          VDVTRKVVQLSHAIG---VTVEGELGCLGSLETGEAGEEDGIGAEGKLDHSMLLTDPEQAADFVKATQLDALAIAIGTSHGAYKFTRKPTG---------
Cupriavidus_necator              IDVTRKVVQLAHAVG---VTVEGELGCLGSLETGEAGEEDGIGAVGVLDHSMLLTDPEQAADFVKATQLDALAIAIGTSHGAYKFTRKPTG---------
Acidovorax_citrulli              VDVTQKVVAMAHKVG---ATVEGELGCLGNLETGDAGEEDGIGAEGKLDHSQMLTDPEEAAQFVKATQLDALAIAIGTSHGAYKFSRPPTG---------
Comamonas_testosteroni           VDVTQKVVAMAHQIG---ATVEGELGCLGNLETGEAGEEDGIGAEGKLDHSQMLTDPEEAAVFVKATQLDALAIAIGTSHGAYKFSRKPTG---------
Acidovorax_radicis               VEVTRKVVDMAHKVG---VTVEGELGCLGNLETGEAGEEDGIGADVKLDHSQMLTDPEEAAVFVKATQLDALAIAIGTSHGAYKFSRKPTG---------
Limnobacter_sp                   LEVTRKVVEMAHKVG---VTVEGELGCLGNLETGEAGEEDGIGADVKLDHSQMLTDPEEAAVFVKGTQLDALAIAIGTSHGAYKFSRKPTG---------
Hylemonella_gracilis             VDVTRKVVDMAHKVG---VTVEGELGCLGNLETGEAGEEDGIGAEGKLDHSQLLTDPEEAAQFVKATQLDALAIAIGTSHGAYKFSRPPTG---------
Methyloversatilis_universalis    VDVTRKVVEMAHRIG---ATVEGELGCLGNLETGEAGEEDGIGAEGKLDHSQMLTDPEEAAVFVKATQLDALAIAIGTSHGAYKFSRKPTG---------
Alicycliphilus_denitrificans     VRVTRQVVEMAHKVG---ATVEGELGCLGSLETGEAGEEDGIGAAGKLSHAQMLTDPEEAAQFVKTTQLDALAIAIGTSHGAYKFTRPPTG---------
Delftia_sp                       VDVTRKVVEMAHKLG---ATVEGELGCLGSLETGMAGEEDGVGAEGVLDHSALLTDPEEAAQFVKATQLDALAIAIGTSHGAYKFTRKPTG---------
Delftia_acidovorans              VDVTRKVVEMAHKLG---ATVEGELGCLGSLETGMAGEEDGVGAEGVLDHSALLTDPEEAAQFVKATQLDALAIAIGTSHGAYKFTRKPTG---------
Ralstonia_pickettii              VDVTRRVVEMAHKVG---VTVEGELGCLGSLETGMAGEEDGHGAEGKLDHSSLLTDPEEAAQFVKATQLDALAIAIGTSHGAYKFTRKPTG---------
Variovorax_paradoxus             VDVTKKVSDMAHRLG---VTVEGELGCLGSLETMKGDKEDGHGTDDTMTREQLLTDPEQAADFVKRTQIDALAIAIGTSHGAYKFTREPTG---------
Hydrogenophaga_sp                VDVTKKVVDMAHKLG---VTVEGELGCLGSLETMKGDKEDGHGTDATMTREQLLTDPEQAADFVKKTQLDALAIAIGTSHGAYKFTRKPTG---------
Ramlibacter_tataouinensis        VEVTRKVVGMAHKVG---VTVEGELGCLGSLETMKGDKEDGHGTDATMTREQLLTDPEQAADFVKKTQLDALAIAIGTSHGAYKFTRKPTG---------
Polaromonas_naphthalenivorans    VEVTRKVVEMAHATG---VSVEGELGCLGSLETMQGDKEDGHGTDAVMTHDQLLTDPEQAADFVKRTQLDALAIAIGTSHGAYKFSRKPTG---------
Methylibium_petroleiphilum       VEVTRKVVDMAHKLG---VTVEGELGCLGSLETMKGDKEDGHGTDATMTVEQLLTDPEEAADFVKRTQLDALAIAIGTSHGAYKFTRKPTG---------
Achromobacter_xylosoxidans       VEVTKKVVDTAHKLG---VTVEGELGCLGSLETMEGDKEDGHGADGKLTMDQLLTDPEQAADFVRRTQLDALAIAIGTSHGAYKFTRKPTG---------
Achromobacter_arsenitoxydans     VEVTKKVVDIAHKLG---VTVEGELGCLGSLETMEGDKEDGHGADGKLTMDQLLTDPEQAADFVRRTQLDALAIAIGTSHGAYKFTRKPTG---------
Bordetella_petrii                VDVTKKVVDIAHKLG---VTVEGELGCLGSLETMKGDKEDGHGAEGTMTREQLLTDPEQAADFVRKTQLDALAIAIGTSHGAYKFTRKPTG---------
Bordetella_pertussis             VDVTKKVVDMAHKLG---VTVEGELGCLGSLETMQGDKEDGHGADGKLTMEQLLTDPEQAADFVRRTQLDALAIAIGTSHGAYKFTRKPTG---------
Advenella_kashmirensis           VEVTKKVVDLAHKVG---VTVEGELGCLGSLETMKGDKEDGHGAEGTMTMEQLLTDPEQAADFVRRTQLDALAIAIGTSHGAYKFTRKPTG---------
Pusillimonas_sp                  LDVTRRVVEMAHKLG---VTVEGELGCLGSLETMKGDKEDGHGAEGTMTMEQLLTDPEQAADFVRQTQLDALAIAIGTSHGAYKFTRKPTG---------
Alcaligenes_faecalis             VEVTRKVVDMAHKLG---VTVEGELGCLGSLETMQGDKEDGHGADGVLTLDQLLTDPEQAAEFVSRTQLDALAIAIGTSHGAYKFTRKPTG---------
Taylorella_equigenitalis         VETTREVVQMAHAIG---VSVEGELGVLGSLETMKGDKEDGHGAEGTLTRDQLLTDPEQAAEFVKATQVDALAIAIGTSHGAYKFTRKPTG---------
Parasutterella_excrementihomin   VNVTKEVVNMAHRVG---VSVEGELGCLGSLETMKGDKEDGHGTDAEMTRDQLLTDPDQAAQFVEETQVDALAIAIGTSHGAYKFTRKPTG---------
Burkholderiales_bacterium        VNVTKEVVNMAHRVG---VSVEGELGCLGSLETMKGDKEDGHGTDAEMTRDQLLTDPDQAAQFVEETQVDALAIAIGTSHGAYKFTRKPTG---------
```

```
Sutterella_wadsworthensis        LETTRRVVEMAHSIG---VSVEGELGCLGSLETMKGDKEDGHGTDAKMTREQLLTDPDQAAEFARETQLDALAIAIGTSHGAYKFTRRPTG---------
Xanthobacter_autotrophicus       AAVTAKVVELAHAVG---VSVEGELGCLGSLETGKGEAEDGHGAEGALDHDKLLTDPEEAAQFVKATQCDALAIAIGTSHGAYKFTRKPTG---------
Magnetococcus_marinus            VATTAKVVEFSHAIG---VSVEGELGVLGSLETGMAGEEDGVGAEGKMEKDALLTDPEEAARFVKETHVDALAIAIGTSHGAYKFTRKPTG---------
Nostoc_punctiforme               VNVTREVVNVAHALG---VSVEGELGCLGSLETGAGEAEDGHGFEGTLDHSQLLTDPDEAVDFVEATQVDALAVAIGTSHGAYKFTRKPTG---------
Anabaena_variabilis              VNVTREVVNVAHALG---VSVEGELGCLGSLETGAGEAEDGHGFEGTLDHSQLLTDPDEAVNFVEATQVDALAVAIGTSHGAYKFTRKPTG---------
Microcystis_aeruginosa           VNVTAEVVKVAHSIG---ASVEGELGCLGSLETGKGEAEDGHGFEGELDHSMLLTDPDEAVDFVERTQVDALAVAIGTSHGAYKFTRKPTG---------
Lyngbya_sp                       VDVTGEVVKVAHSIG---VSVEGELGCLGSLETGMGEAEDGHGAEGKLSHDQLLTDPDEAVEFVEKTQVDALAVAIGTSHGAYKFTRKPTG---------
Arthrospira_platensis            VRVTAEVVNVAHSIG---VSVEGELGCLGSLETGMGDKEDGHGAEGVLTRDQLLTDPDQAVEFVERTQVDALAVAIGTSHGAYKFSRKPTG---------
Oscillatoria_sp                  VNVTSEVVKVAHSIG---VSVEGELGCLGSLETGMGEAEDGHGAEGVLSHDQLLTDPDQAVDFVEQTQVDALAVAIGTSHGAYKFTRKPTG---------
Cyanothece_sp                    VAVTAEVVKVAHAVG---ASVEGELGCLGSLETGKGDKEDGHGFEGTLSKEQLLTDPDEAVEFVERTQVDALAIAIGTSHGAYKFTRKPTG---------
Synechococcus_elongatus          VEVTRQVVDVAHSIG---VSVEGELGCLGSLETGKGEAEDGHGFEGTLDHSQLLTDPDQAVDFVEKTQVDALAVAIGTSHGAYKFTRKPTG---------
Prochlorococcus_marinus          VAVTKKVVDFAHSVG---VSVEGELGCLGSLETGKGEAEDGHGFEGELSKDMLLTDPSEASDFVAKTKVDALAIAIGTSHGAYKFTRKPTG---------
Paulinella_chromatophora         VNVTKEVVDVAHAIG---VSVEGELGCLGSLETGKGEAEDGHGFEGSLSKEQLLTDPTEAADFVARTKVDALAIAIGTSHGAYKFTRKPTG---------
Synechococcus_sp                 VAVTAEVVKVAHAVG---VSVEGELGCLGSLETGRGEAEDGHGAEGALSHDQLLTDPDQAVDFVEQTQVDALAVAIGTSHGAYKFSRKPTG---------
Gloeobacter_violaceus            VEVTAKVTEVAHALG---VSVEGELGCLGSLETGAGDKEDGHGFEGTLSHDQLLTDPDEAADFVERTGVDALAIAIGTSHGAYKFSRKPDG---------
Sinorhizobium_meliloti           VAVTAEVVKIAHAAG---VSVEGELGCLGNLETGAGDKEDGHGFEGKLSREELLTDPEQALDFVSKTGVDALAVAIGTSHGAYKFTREPDG---------
Stappia_aggregata                VGVTKTVTDMSHLGG---ISVEGELGVLGSLETGMGDKEDGHGAEGKLSHDQLLTDPEEAVKFVQETKVDALAIAMGTSHGAYKFTRKPDG---------
Labrenzia_alexandrii             VGVTKTVTDMAHLGG---ISVEGELGVLGSLETGMGDKEDGHGAEGKLTEDQLLTDPDEAVKFVKETKVDALAIAMGTSHGAYKFTRKPDG---------
Polymorphum_gilvum               VGVTKTVVDMAHLGG---ISVEGELGVLGSLETGMGDKEDGHGAEGKLSHDQLLTDPDEAVKFVRETKVDALAIAMGTSHGAYKFTRKPDG---------
Bradyrhizobium_sp                VGVTKTVTDMAHLGG---ISVEGELGVLGSLETGMGDKEDGHGAEGKLSHDQLLTNPDEAVKFVQETKVDALAIAMGTSHGAYKFTRKPDG---------
Agrobacterium_radiobacter        VGVTKTVTEMAHLGG---VSVEGELGVLGSLETGMGEAEDGHGAEGKLSHDQLLTNPDEAVKFVRETKVDALAIAMGTSHGAYKFTRRPDG---------
Rhizobium_sp                     VGVTKTVTEMAHLGG---ISVEGELGVLGSLETGMGEAEDGHGAEGKLSHDQLLTNPDEAVKFVRETKVDALAIAMGTSHGAYKFTRRPDG---------
Brucella_melitensis              VSVTRQVSEMSHLGG---ISVEGELGVLGSLETGMGDVEDAHGAEGKLSHDQLLTDPDEAVKFVRETKVDALAIAMGTSHGAYKFSRKPDG---------
Brucella_suis                    VSVTRQVSEMSHLGG---ISVEGELGVLGSLETGMGDAEDGHGAEGKLSHDQLLTDPDEAVKFVRETKVDALAIAMGTSHGAYKFSRKPDG---------
Ochrobactrum_anthropi            VSVTRQVSEMSHLGG---ISVEGELGVLGSLETGMGDAEDGHGAEGVLSHDQLLTDPDEAVKFVRETKVDALAIAMGTSHGAYKFSRKPDG---------
Sinorhizobium_fredii             VKVTRSVSDTAHWAG---VSVEGELGVLGSLETGTGEAEDGHGVEGKLDPHQLLTDPEEAAKFVAETKVDALAVAMGTSHGAYKFSRRPDG---------
Mesorhizobium_amorphae           SGITKRVVDMAHWGG---VSVEGEIGVLGSLESGGGEQEDGHGVEGAISHDQLLTDPEQAVQFVKDTHVDALAVAMGTSHGAYKFSRKPDG---------
Mesorhizobium_loti               SGITKRVVDMAHWGG---VSVEGEIGVLGSLESGGGEQEDGHGVEGAISHDQLLTDPEQAVEFVKDTHVDALAVAMGTSHGAYKFSRKPDG---------
Methylobacterium_nodulans        VEITRKVAEMAHWAG---VSVEGELGVLGSLESGKGEAEDGHGAEGALSHDQLLTDPDEAVKFVQATKVDALAVAMGTSHGAYKFTRKPDG---------
Methylobacterium_radiotolerans   VEITRNVTKMAHWAG---VSVEGELGVLGSLESGQGEAEDGHGAEGTLSHDQLLTDPDEAVKFVEATKVDALAVAMGTSHGAYKFTRKPDG---------
Phenylobacterium_zucineum        VRVTRAVVDMAHWVG---ASVEGELGVLGSLETGMGEKEDGHGFEGKLGHDQLLTNPDQAVEFVKATEVDALAIAMGTSHGAYKFTRKPDG---------
Caulobacter_sp                   VAVTRKVVEMAHACG---VSVEGELGVLGSLESGMGEAEDGHGFEGVLSHSELLTDPDQAVDFVQQTGVDALAIAMGTSHGAYKFTRKPDG---------
Caulobacter_crescentus           VEVTRKVVQMAHSCG---VSVEGELGVLGSLETGMGEAEDGHGFEGKLSHDELLTDPDQAVDFVAQTGVDALAIAMGTSHGAYKFTRKPDG---------
Paracoccus_denitrificans         VAVTAKVSEAAHAVG---ASVEGELGVLGSLETGEAAAEDGSGAEGKLDHSQLLTDPDQAVDFVARTRCDALAIACGTSHGAYKFSRKPDG---------
Paracoccus_sp                    VAVTAKVAEAAHAVG---ASVEGELGVLGSLETGEAAAEDGSGAEGKLDHSQLLTDPDQAVDFVTRTRCDALAIACGTSHGAYKFSRKPDG---------
Pelagibaca_bermudensis           VEITRRVTTAAHAVG---ASVEGELGVLGSLETGEAAAEDGSGAEGKLSHDQLLTDPDQAVEFVMATKCDALAIACGTSHGAYKFTRKPDG---------
Citreicella_sp                   VEITRRVTTAAHAVG---ASVEGELGVLGSLETGEAAAEDGSGAEGKLSHDQLLTDPEQAVDFVLATRCDALAIACGTSHGAYKFTRKPDG---------
Rhodopseudomonas_palustris       VDITRRVTDMAHWVG---ASVEGELGVLGSLEHGGGEQEDGHGVEGKVSREQLLTDPDQAVDFVRATKVDALAIAMGTSHGAYKFSRKPDG---------
Starkeya_novella                 VTITRRVVDAAHWVG---ASVEGELGVLGSLEHGSGEQEDGHGAEGALSHDQLLTDPDQAVDFVARTKVDALAIAMGTSHGAYKFTRKPDG---------
Azospirillum_amazonens           VDITRRVAEAAHWVG---ASVEGELGVLGSLETGQGEAEDGHGAEGVLSLDQLCTDPDQALDFVAATRVDALAIAMGTSHGAYKFSRQPDG---------
Rhodobacter_sphaeroides          VDITARVSHMAHWVG---ASVEGELGVLGSLETGESEAEDGHGAEGKLDHSQLLTDPDQAVDFVKKTQVDALAIACGTSHGAYKFSRKPDG---------
```

```
Aurantimonas_manganoxydans       VAITERVTRMAHWVG---ASVEGELGVLGSLESGEGEKEDGHGFEGKLSEDQLLTDPDQAVDFVRQTHVDALAIAMGTSHGAYKFTRKPDG---------
Octadecabacter_arcticus          VQVTSKVVELAHMVG---VSVEGEIGHLGSLETGKGEAEDGHGFEGSLSRDMLLTDPEEAETFVSLTNVDALAIAIGTSHGAYKFTRKPDG---------
Sulfitobacter_sp                 ATVTAKVSEMAHLVG---ASIEGEIGHLGSLETGKGEAEDGHGFEGELSREQLVTNADEAAEFVRRTEVDALAVAIGTSHGAYKFTRKPDG---------
Rhodospirillum_photometricum     CTVTGKVAECAHLVG---VSVEGELGCLGSLETGAGEKEDGHGFEGTLDHSQLLTDPDQAADFVARTQVDALAVAIGTSHGAYKFTRKPTG---------
Pirellula_staleyi                VQVTKDVVKLAHARN---VGVEGELGCLGSLESGHGEAEDGHGAEGQLSHDQLLTDPDEAAQFVAATGVDALAVAIGTSHGAYKFTKKPDG---------
Blastopirellula_marina           VAVTKKVVEMAHAKN---VGVEGELGCLGSLESGTGEAEDGHGAVGELSHDQLLTDPDEAFEFVKATGVDALAVAIGTSHGAYKFTSKPTG---------
Rhodopirellula_baltica           VKVTAEVVKMAHARG---VSVEGELGCLGSLEDGGGEQEDGHGAVGTLTHDQLLTDPDEAQRFVEETGVDALAVAIGTSHGAYKFTSKPTG---------
Koribacter_versatilis            VKATRAVVELAHDKG---VTVEGEIGVLGGIEDGHGAGGTG------LEH---ITDPEQAVEFVEKTGLDALAIAIGTSHGAYKFNKKPDG---------
Solibacter_usitatus              VAVTKEVVELAHAKG---VTVEGEIGCLGGIEDGHGAGGDG------LSH---LTDPDQAVEFVKQTGLDALAIAIGTSHGAYKFSSKPTG---------
Anaerolinea_thermophila          IAITRRVVEAAHARG---IVVEAELGQLGGVEEHVSVD----------ESNAKLTDPDNAKEFVERTGCDSLAVAIGTSHGAFKFTGN------------
Diplosphaera_colitermitum        VAITKRVVQAAHAKG---ISVEAELGMLGGVEEDVKVE----------DGHATLTDPKEAEEFVKQTGCDSLACAIGTSHGAFKFKGK------------
CONSERVATION                                          * * *     *                                        *   *  **

                                         310       320       330       340       350       360       370       380       390       400
                                 ....|....|....|....|....|....|....|....|....|....|....|....|....|....|....|....|....|....|....|....|
Chlorobium_phaeovibrioides       DPKIRLDILQEIEKRIP-GFPIVLHGSSSVPQDLVKTINEHGGRLKDAIGIGEDQLRLASKSAVCKINIDSDGRLAMTAAIRKVLD--EKPEEFDPRKYL
Pelodictyon_phaeoclathratiform   DPKIRLDILTEIEHRIP-GFPIVLHGSSSVPQDLVKTINEHGGKLKDAIGISEDQLRLAAKSAVCKINIDSDGRLAMTAAIRKVLD--EKPEEFDPRKYL
Chlorobium_chlorochromatii       DPKIRLDILAEIEKRIP-GFPIVLHGSSSVPQDLVATINQNGGKLKDAIGISEEQLRLAAKSAVCKINIDSDGRLAMTAAIRKVLA--EKPEEFDPRKYL
Prosthecochloris_aestuarii       DHKIRLDILSEIEKRIP-GFPIVLHGSSSVPQDLVKTINEHGGKLKDAIGISEEQLRKAAQSAVCKINIDSDGRLAMTAAIRKVLD--EKPEEFDPRKYL
Chlorobaculum_parvum             DHKIRLDILAEIEKRIP-GFPIVLHGASSVPQDLVQMINAHGGKLKDAVGIGEDQLREAARSAVCKINIDSDGRLAMTAAVRKVLD--EKPEEFDPRKYL
Victivallis_vadensis             NPKIRLDILAEIEKRIP-GFPIVLHGSSSVPQDLVKIINENGGKLKDAIGIGEDQLRAAAKSAVCKINIDSDGRLAMTAAIRKVFN--EKPAEFDPRKYL
Brachyspira_murdochii            NPEIRLDILKEIEKKIP-GFPIVLHGSSSVPQEYVKMINEYGGKLDDAIGIPEEQLREASKSAVCKINIDSDSRLAMTAAIRKVFH--DNPKEFDPRKYL
Brachyspira_pilosicoli           NPQIRLDILKEIEKKIP-GFPIVLHGSSSVPQEYVKMINENGGKLDDAIGIPEEQLREASKSAVCKINIDSDSRLAMTAAIRKVFH--DNPKEFDPRKYL
Alistipes_indistinctus           PPPLAFDVLDGVMKELP-GFPIVLHGSSSVPQEEVATINKYGGALKDAIGIPEEELRKAAASAVCKINIDSDSRLAMTAAIRKVFV--EKPAEFDPRKYL
Tannerella_sp                    PPPLRFDVLEGVEKELP-GFPIVLHGASSVPQEEVATINKYGGALKDAIGIPEEQLRKAAKSAVCKINIDSDSRLAMTAAIREVFA--TKPAEFDPRKYL
Tannerella_forsythia             PPPLAFDILEAVEKELP-GFPIVLHGSSSVPQEDVDTINKYGGALKDAIGIPEEQLRKAAKSAVCKINIDSDSRLAMTAAIRKVFA--EKPAEFDPRKYL
Bacteroides_intestinalis         PPPLAFEVLDAVMEKLP-GFPIVLHGSSSVPQEEVETINKFGGKLEAAIGIPEEELRKAAKSAVCKINIDSDSRLAMTAAIRKVFA--EKPAEFDPRKYL
Bacteroides_uniformis            PPPLEFAVLDAVMEKLP-GFPIVLHGSSSVPQEEVDTINKYGGKLEAAIGIPEEQLRKAAKSAVCKINIDSDSRLAMTAAIRKTFA--EKPAEFDPRKYL
Parabacteroides_merdae           PPPLAFNVLDAVMEKLP-GFPIVLHGSSSVPEEEVETINKYGGALKAAIGIPEEELRKAAKSAVCKINIDSDSRLAMTAAVRKVFA--EKPAEFDPRKYL
Parabacteroides_sp               PPPLAFDVLDAVMEKLP-GFPIVLHGSSSVPQEEVETINKFGGALKAAIGIPEEWLRKAATSSVCKINIDSDSRLAMTAAIRKTFA--EKPAEFDPRKYL
Capnocytophaga_sp                PPPLAFDVLDGVMKELP-GFPIVLHGSSSVPQEEVDTINKYGGKLEAAIGIPEDELRKAAKSAVCKINIDSDSRLAMTAAVRKVFA--EKPAEFDPRKYL
Paraprevotella_clara             PPPLAFDVLDGVMKELP-GFPIVLHGSSSVPQEEVDTINKYGGKLEAAIGIPEDELRKAAKSAVCKINIDSDSRLAMTAAVRKVFA--EKPAEFDPRKYL
Paraprevotella_xylaniphila       PPPLAFDVLDGVMKELP-GFPIVLHGSSSVPQEEVDTINKYGGKLEAAIGIPEDELRKAAKSAVCKINIDSDSRLAMTAAVRKVFA--EKPAEFDPRKYL
Bacteroides_salanitronis         PPPLAFDVLDAVMEKLP-GFPIVLHGSSSVPQEYVDMINQYGGKLEAAIGIPEEELRKAAKSAVCKINIDSDSRLAMTAAVRKVFV--EKPAEFDPRKYL
Dysgonomonas_gadei               PPPLRFDVLEGCEKELP-GFPIVLHGASSVPQEEVETINKYGGALKDAIGIPEEQLRKAAKSAVCKINIDSDSRLAMTAAVREVFA--TKPGEFDPRKYL
Dysgonomonas_mossii              PPPLRFDILEAIEKELP-GFPIVLHGASSVPQEEVATINKYGGALKDAIGIPEEQLRRAAKSAVCKINIDSDSRLALTAAVREVFA--TKPAEFDPRKYL
Paludibacter_propionicigenes     PPPLRFDILEEIEKQIP-GFPIVLHGASSVPQEYVATINKFGGALKDSIGIPEEQLRRAAASAVCKINIDSDGRLAMTAAVREVLA--TKPGEFDARKYL
Alistipes_sp                     PPELRFDILAEIEKELP-GFPIVLHGSSSVPQEYVKIINTHGGALKDAVGIPEEQLRKAAKSAVCKINIDSDGRLAMTAAIRKIFV--DQPAEFDPRKYL
Odoribacter_laneus               PPPLRFDILKEIEKRIP-GFPIVLHGSSSVPQDKVAIINKYGGALKDAIGIPEDQLREAAKSAVCKINIDSDGRLAMTAAIREVFA--LKPAEFDPRKYL
Porphyromonas_endodontalis       PPPLRFDILEEVQRRIP-GFPIVLHGSSSVPQDKVKIINANGGALKDSIGIPEEQLRKASKTNVCKVNIDSDGRLAMTAAIREVFV--NKPAEFDPRKYL
Candidatus_Azobacteroides        PPPLRFDILEAIEERIP-DFPIVLHGASSVPQEQVAVINNNGGKLKDAIGIPEEQLRCAAKSAVCKINIDSDGRLAMTAAIRSTLN--MHPEWFDPRQYL
```

```
Sphaerochaeta_pleomorpha            PPPLRFDILEEIEKKLP-GFPIVLHGSSSVPMEYVNMINEYGGKLKDSVGIPEEQLRLASKSAVCKINIDSDARLAMTAMVRKVFA--EKPGEFDPRKYL
Sphaerochaeta_coccoides             PPPLRFDILKEIEDKLP-GFPIVLHGSSSVPVEFVKMINEHGGKLKDSVGIPEEQLRQAAKSAVCKINIDSDGRLAMTGTIRRVFD--EKPGEFDPRKYL
Spirochaeta_caldaria                PPPLRFDILEEIEKRIP-GFPIVLHGSSSVPIEYVKLIEKYGGSMKDSVGIPEEQLRRAAKSAVCKINIDSDGRLAMTAMIRKVFA--EKPDEFDPRKYL
Treponema_saccharophilum            PPPLAFDVLEGVEKMIP-GFPIVLHGSSSVPVEYVKMIEQFGGKIPDSVGIPEEQLRKAAKSAVCKINIDSDGRLAMTAMIRKVFA--EHPDEFDPRKYL
Treponema_sp                        PPPLAFDVLEAIEKKLP-GFPIVLHGSSSVPVEYVKIIEQFGGKIPDSVGIPEEQLRKAAKSAVCKINIDSDGRLAMTAAIRRHFV--EHPGDFDPRQYL
Melioribacter_roseus                PPPLRFDILEEIEKRIP-GFPIVLHGASSVPPELVKIINSNGGRLKDAVGIPEDQLRRAARSAVCKINIDSDGRLAMTAAIRKYLN--EHPEEFDPRKYL
Spirochaeta_thermophila             PPPLRFDILEEIERRLP-GFPIVLHGSSSVPPQYVEMINKYGGKLKDAVGIPEDQLRKAARSAVCKINIDSDGRLAMTAMIRKVLH--EQPEVFDPRKYL
Prevotella_multisaccharivorax       PPPLAFNVLHEIEKKLP-GFPIVLHGSSSVPQKYVDIINKYGGKLPDAVGIPEDQLRKASKSAVCKINIDSDSRLAYTAGVRETLA--NHPEYFDPRQYG
Prevotella_disiens                  PPPLAFDVLEAVEKELP-GFPIVLHGSSSVPEKYVEIINANGGNMPNAIGIPEEQLRKATKSAVCKINIDSDSRLAFTAGVRETMK--AHPEYFDPRQYC
Fibrobacter_succinogenes            PPPLAFDVLHAIEQKLP-GFPIVLHGSSSVPQDEVDTINAHGGKLPDAVGIPEEQLREASRSAVCKINIDSDSRLAMTAAIRKYFD--EHPEHFDPRQYL
Elusimicrobium_minutum              VPPLRFDILEEVSNRLP-GFPIVLHGASSVDQKAVATINQYGGKLDNAVGIPEDQLRKAAKSAVCKINVDSDGRLVMTAAVRKVFV--TKPEEFDPRKYL
Hippea_maritima                     PPPLRFDILEEVEKRIP-GFPIVLHGASSVLQEYVEMINKYGGDLSGAVGVPEDQLRKAAQSAVCKINIDSDGRLAMTAKVREILW--TQPAEFDPRKYL
Deferribacter_desulfuricans         VPPLRFDILEEIERRLP-GFPIVLHGASSVIPEYVELINKYGGNLEGAVGVPEDQLRKAATSAVCKINIDSDGRLAFTAKVREFLW--NNPKEFDPRKYL
Calditerrivibrio_nitroreducens      VPPLRFDILEEVEKRLP-GFPIVLHGASSVVPEYVELINKYGGKLEGAVGVPEEQLRRAAASAVCKINIDSDGRLAFTAKVREYLA--NNPKEFDPRKYL
Flexistipes_sinusarabici            VPPLRFDILEEVENRLP-GFPIVLHGASSVMQEYVDIINKYGGNLEGTAGVPEDQLRKAAKSAVCKVNIDSDGRLAFTAKVREFLY--NNPEVFDPRKYL
Pelobacter_carbinolicus             PPALRFDILSEVEKRIP-GFPIVLHGASSVVPAYVELINRYGGNLEGAVGVSEEQLRRAASSAVCKINIDSDGRLAMTAKVREYLA--NNPKDFDPRKYL
Geobacter_lovleyi                   VPPLRFDILAECEKRLP-GFPIVLHGASSVVQEYVELINQNGGKMEGAVGVPEEQLRQAAASAVCKINIDSDGRLAVTAKVREYFG--KDPKEFDPRKYL
Desulfurispirillum_indicum          VPPLRFDILEEIERRLP-GFPIVLHGASSVVPEYVDMINAYGGDIKGALGVPEEQLRRAASSAVCKINIDSDGRLAFTAAIRKALA--ENPAEFDPRKYL
Trichomonas_vaginalis               KAEIRLDILHEIEKKLP-GFPIVLHGSSSIPQEYVEMVNKYGGHMPEAVGIPEHQLREAAASAVCKINIDSDGRMVMTGTIRRLFS--EHPDWFDPRQYL
Entamoeba_dispar                    HPRIRLDILHEVEKRIP-GFPIVLHGSSSVPQEWVAVINQYGGKLESAIGIPEDQIREAVKSAVCKVNIDSDGRLAMTGSIRRYLA--EHPKDFDPRQYL
Entamoeba_histolytica               HPRIRLDILHEVEKRIP-GFPIVLHGSSSVPQEWVAVINQYGGKLESAIGIPEDQIREAVKSAVCKVNIDSDGRLAMTGSIRRYLA--EHPKDFDPRQYL
Leptotrichia_goodfellowii           KPQIRLDILKEIEQRIP-GFPIVLHGSSSVPKKFVDMINKFGGQIADAIGIPDEQLREASKSAVSKINVDTDGRLAFTAGIREVLA--TKPGEFDPRKYV
Leptotrichia_hofstadii              DPKLRLDILEEIEKRIP-GFPIVLHGSSAVPHQFVEMINQYGGKIADAIGIPDSELRKAAKSAVAKINVDTDGRLAFTAGIREVFA--KNPGEFDPRKYV
Streptobacillus_moniliformis        DPKIRLDILKEIEKRIP-GFPIVLHGSSSVPQQFVKKIIQYGGAMKDAIGIPDEQLLLSSKSAVAKINVDTDGRLAFTAGVREVLA--NNPGEFDPRKYA
Fusobacterium_mortiferum            DPKLRLDILEEIERRIP-GFPIVLHGSSAVPQQYTTMIKEFGGEVKDAIGIPDSELRKAAKSAVAKINVDTDGRLAFTAAIRRVLG--TTPKEFDPRKYL
Fusobacterium_gonidiaformans        DPKLRLDVLDAVAEKLG-SFPIVLHGSSAVPKKYVDMIKEFGGEMKDAIGIPDSELRGATKSTVAKINVDTDGRLAFTAGVRQVLG--TNPKEFDPRKYL
Alkaliphilus_metalliredigens        -PKLDFDRLAKISSLLP-NFPLVLHGASTVLPEFVEMCNQYGGNIPGAQGVPEEMLTQAASLGVCKINIDTDLRLAMTAAIRKELI--ENPGEFDPRKYL
Veillonella_sp                      -PYLDFDRLKKVGELLP-NFPIVLHGASSVLPEFVAKCNEFGGNIPGAQGVPEAMLRQAAQMSVCKINIDTDLRLAMTASVREYLA--QNPSEFDPRKYL
Acetonema_longum                    -PSLDFARLEKISNLLP-EFPLVLHGASTVLPEFVAKCNQYGGKIDGAQGVPEEMLKQAGKLGVCKINIDTDLRLAMTASIREHFA--HNPGDFDPRQYL
Thermosinus_carboxydivorans         -PTLDFERLEKISKMLP-GFPLVLHGASSVLPEFVAKCNQYGGKIQGAQGVPEEMLLKAGQMGVCKINIDTDLRLAMTASIREHLA--LHPGDFDPRQYL
Pelosinus_fermentans                -PSLDYARLEKISSLLP-NYPLVLHGASTVLPEFVAKCNQFGGHIPGAQGVPEDMLLRAGKFGVCKINIDTDLRLAMTASIREHFV--NNPSDFDPRQYL
Desulfosporosinus_sp                -AKLDFARLEEITALLP-GFPLVLHGASTVLPDFVAKCNQYGGELKGAQGVPEELLRKAGTMGVCKINIDTDLRLAMTASIREYLA--VNPADFDPRQYL
Desulfosporosinus_acidiphilus       -PKLDFARLQKITALLP-GFPLVLHGASTVLPEFVAKCNQYGGELKGAQGVPEELLHKAGTMGVCKINIDTDLRLAMTASIREHFA--VNPADFDPRQYL
Acidaminococcus_sp                  -PELDFARLEKISNLLP-NFPLVLHGASTVIPAFVEECNRYGGKLDGAQGVPEDMLLKAGKYGVCKINIDTDLRLAMTASIRKYLA--EHPEDFDPRSYL
Acidaminococcus_intestini           -PELDFARLEKISNLLP-NFPLVLHGASTVIPAFVEECNRYGGKLDGAQGVPEDMLLKAGKYGVCKINIDTDLRLAMTASIRKYLA--EHPEDFDPRSYL
Phascolarctobacterium_succinat      -PELDFARLEKNTNMLP-GYPLVLHGASTVIPSFVEECNKYGGKLDGAQGVPEDMLLQAGKFGVCKINIDTDLRLAMTASIRKHLV--ENPGDFDPRQYL
Caldicellulosiruptor_kristjans      -PRLDFERLQKIVEKLPKDFPIVLHGASTVLPEFVEMCNKYGGNIPGAKGVPEDMLRKAAELGVRKINIDTDLRLAMTAAIRKHLY--EHPDHFDPRQYL
Caldicellulosiruptor_lactoacet      -PRLDFERLQKIVEKLPKDFPIVLHGASTVLPEFVEMCNKYGGNIPGAKGVPEDMLRKAAELGVRKINIDTDLRLAMTAAIRKHLY--EHPDHFDPRQYL
Tepidanaerobacter_acetatoxydan      -PKLDFERLEKISNMLP-GFPIVLHGASSVPSEFVELCNKYGGQIPGAQGVPESMLRKAAEMGVCKINIDTDLRLAMTASVRKYLA--ENPSHFDPRQYL
Thermosediminibacter_oceani         -AYLDFERLKKITEKLP-GFPLVLHGASSVLPEYVEICNRYGGNIPGAQGVPEDMLRKAASMGVCKINIDTDLRLAMTATIRRYFS--ENPSEFDPRKYL
Caloramator_australicus             -AKLDFERLEEIQKKLP-GFPLVLHGASAVMPEFVEKCNRYGGKLEGAQGIPVEMLRKAASMAICKINMDTDLRLAMTATIREVFA--TKPEEFDPRKYL
```

```
Eubacterium_saphenum            -PRLDFERLKEIHALIP-DVPLVLHGASTVIPEFVDKCNEYGGEIPGARGVPEDMITEAVKHGVCKVNIDTDLRLAMTAEIRKFMV--ENPAEFDPRKYL
Eubacterium_infirmum            -PYLDYERLKKIHSLLP-DTPLVLHGASSVLKEFVDRCNKFGGQIPGAQGVPEEMIRESTKYGICKVNIDTDLRLAMTAEIRRILI--ENPSEFDPRKYL
Acetobacterium_woodii           -PRLDFDRLHKISDLLP-QYPLVLHGASSVPKEFVDLCNQYGGQIPGAQGVPEEMLRKAAKSGVCKINIDTDLRLAMTASIRQVFV--ENPGWFDPRQYL
Flavonifractor_plautii          PPPLRFDILDEVSKRLP-GFPIVLHGSSSVPQEYVKMINENGGKMPDAVGIPEEQLRQAARLAVCKINIDSDLRLAMTGTIRQFFA--EHPDKFDPREYL
Oscillibacter_valericigenes     KPQLRFDVLEEVSRRLP-GFPIVLHGSSSVPKEFVEKINKFGGNMPGAVGVPEDQLRHAAELSVCKINIDSDLRLAMTACIREHFA--EAPSDFDPRQYL
Bryantella_formatexigens        PPPLRFDVLEECIKRLP-GFPIVLHGSSSVPQEFVKMVNQYGGNMPDAIGIPEEELRHAAELAVCKINIDSDIRLAMTGNIRKYFA--EHPDHFDPRQYL
Stomatobaculum_longum           KPQLRLDILEEVKKRLP-GFPIVLHGASSVPQEYVKIINANGGQLKDAIGVPEDQLREAARSAVCKINIDSDLRLGMTAGIRQHFN--EHPDHFDPRQYL
Selenomonas_sputigena           PPELRFDILAEIEKRLP-GFPIVLHGASSVIPKYVKIINDNGGNLADAIGIPEDQLRKAAKSAVCKINIDSDLRLAMTAGIREHYQ--QHPEHFDPRQYI
Megasphaera_elsdenii            PPTLRFDILEEVSKRLP-GFPIVLHGASSVVPEYVKIINENGGNLADAIGIPEDQLRKAASMAVCKINIDSDLRLGLTAGIRQHMA--QHPEHFDPRQYL
Mitsuokella_multacida           PPELRFDILAEIEKKIP-EFPIVLHGASSVIPKYVKIINENGGKLDDAVGIPEDQLRKAAKSAVCKINIDSDLRLAMTAGIRQHFF--AHPEHFDPRQYC
Selenomonas_infelix             PPPLRFDILEEIEKRIP-GFPIVLHGASSVIPKYVKIINENGGHMPDAVGIPEDQLRRAAKSSVCKINIDSDLRLAMTAGIREHFK--KEPSHFDPRQYL
Centipeda_periodontii           PPPLRFDILEEIEKRIP-GFPIVLHGASSVIPKYVKIINENGGHMPDAVGIPEDQLRRAAKSSVCKINIDSDLRLAMTAGIREHFK--KEPSHFDPRQYL
Megamonas_funiformis            PPPLRFDILEEVAKRLP-NFPIVLHGASSVPQDFVKIINENGGHMPDAVGVPEDQLRKAASMAVCKINIDSDLRLAMTAGIRKHFN--DHPDHFDPRQYV
Anaeroglobus_geminatus          PPELRFDILEEVSKRLP-DFPIVLHGASSVLPEYVKIINENGGKLDKAIGIPEEQLRHAASLAVCKINIDSDLRLALTAGVRQHLA--QHPDHFDPRQYL
Clostridium_thermocellum        -AKLRFDILEEIEKRLP-GFPIVLHGASSVIPEYVDMINKYGGDMPGAKGVPEDMLRKAASMAVCKINIDSDLRLAMTATIRKYFA--ENPSHFDPRQYL
Acetivibrio_cellulolyticus      -ARLRFDILEEVQKRLP-GFPIVLHGASSVMPEYVEMINKFGGNMPGAKGVPEEMLRQAAKMAVCKINIDSDLRLALTGSIRKYFA--ENPSHFDPRQYL
Dictyoglomus_turgidum           -PKLDFDRLREIAKRLP-GFPLVLHGASSVLPEYVEKANQYGAKLGGAKGVPEEMIREATKMGICKVNIDTDLRLAVTATIREVFA--LHPEEFDPRKYL
Dictyoglomus_thermophilum       -PKLDFDRLREIAKRLP-GFPLVLHGASSVLPEYVEKANQYGAKLGGAKGVPEEMIREATKMGICKVNIDTDLRLAVTATIREIFA--LHPEEFDPRKYL
Thermotoga_lettingae            -PKLDFERLHEIAKRLP-NFPLVLHGASSVLQEFVQKANQYGGKLSGAQGVPEDMIRKATTMGVCKVNIDTDLRLAVTATIREIFA--LHPEEFDPRKYL
Thermotoga_thermarum            -PRLDFERLQEIAKRLP-GFPLVLHGASSVLQDLVEKANKYGAKIVGAQGVPEEMIRKATIMGICKVNIDTDLRLAFTATIREVLA--TKPEEFDPRKYL
Caldisericum_exile              -PKLDFERLQEIARKLP-NFPLVLHGASSVLPQYVEKINKYGGNIGDAKGVPEDMLRKATTMGITKINIDTDLRLAMTATIREIFA--LHPEEFDPRKYL
Bilophila_sp                    -AKLDFERLEKIGNLMP-GYPLVLHGASSVPQEFVEMCNTYGGKVAGAAGVPEELLRKAAGMAICKINIDTDIRLAMTASIRKQLV--EHPEEFDPRGYL
Bilophila_wadsworthia           -AKLDFERLEKIGSLMP-GYPLVLHGASSVPQEFVEMCNTYGGKVAGAAGVPEELLRKAAGMAICKINIDTDIRLAMTASIRKQLV--EHPEEFDPRGYL
Lawsonia_intracellularis        -PELDFARLETIGNLLP-DFPLVLHGASSVSQQYVDMCNKYGGKIAGSAGVPEEFLRKAASLAVCKINIDTDIRLAMTASIRKFFV--EHPEEFDPRSYL
Desulfohalobium_retbaense       -PRLDLERLETITTKLP-EFPLVLHGASTVLPELVDMANSYGGQIAGAKGVPEDLLRQAAKSGVCKINIDTDIRLAMTANIRKFLA--ENPAEFDPRKYL
Desulfomicrobium_baculatum      -AKLDFDRLEKIKALLP-DYPIVLHGASSVPQEFVDMANQYGAQIAGAKGVPEDLLRKAAASAVCKINIDTDIRLAMTATIRKYLA--ENPSHFDPRQYL
Desulfovibrio_alaskensis        -ATLDFDRLSTITDKLP-GFPLVLHGASSVPQEFVAMANQYGGEIGGAQGVPEDLLRKSASYGVCKINIDTDIRLAMTACIRKHFA--ENPADFDPRAYL
Desulfonatronospira_thiodismut  -PTLDFDRLEQISSRLP-GYPLVLHGASSVLPEFIDMANRYGGQVSDAKGVPEDFLRRAAKSGVCKINIDTDIRLAMTAVIRKFFH--EKPQEFDPRKYL
Desulfovibrio_piger             -AKLDFERLEEIGKRLP-NYPLVLHGASSVPQEFVEACNKFGGQVGGARGVPEDMLRKAAGMGVCKINVDTDIRLAVTASIRQYLV--EHPEAFDPRAYL
Dialister_succinatiphilus       -PYLDYERLEKVGKLLP-GYPIVLHGASTVIPEFVEKCNQYGGKVLGAKGVPEDMLRKAATMAVCKINIDTDIRLAMTSAIREALY--KDPSNFDPRNYL
Dialister_micraerophilus        -PYLDYERLEKIGKLLP-DYPIVLHGASTVIQHYVKKCNEYGGKVNGAKGVPEEMLRKAATMAVCKINIDTDIRLAMTAAVRQALI--EHPENFDPRKYL
Clostridium_botulinum           EAQLRFDILEEIQSKLP-GYPIVLHGASSVDPESVDTCNKYGGEIRGAKGVPADMLRKASSMAVCKINMDTDLRLAMTAGIRKVLS--EDPKQFDPRKYL
Peptostreptococcus_anaerobius   EANLRFDILEEIQGKLP-NFPIVLHGASAVDPESVATCNKFGGDIAGAKGVPVDMLRKASSMAVCKINMDTDLRLAMTAAIRKFLA--ENPSAFDPRKYI
Arthromitus_sp                  KPSLKFEILEEIQNKLP-GFPIVLHGASAVDPDSVQTCNKYGGKIKDAVGIPVDMLRRASSMAVCKINMDTDLRLAMTANIRKVFA--DNPSEFDPRKYL
Leptonema_illini                -ARLDFERLSRIHTLMP-GYPLVLHGASSVPAALIEEANRYGAKIEDAQGIPEEMLREATTMGVAKINIDTDLRLAAVMALRRAMA--GNPAEFDPRKFF
Anaerofustis_stercorihominis    -PSLDYARLEKVCELIP-NFPIVLHGASSVPQEFVELNNKYGAKIPGAQGVPEEMLAKAAKLGVAKINVDTDIRLAMTAAIRKVFV--EEPEVFDPRGYL
Methanocella_arvoryzae          -ANLDFERLQKIEKLVG--IPIVLHGASGVPKEVLEKAAKYGAKLPGAAGVPNDAIQKAISLGVAKINIDTDIRLTMTAAIRQVLT--EHPEEFDPRKIF
Methanocella_conradii           -AVLDFERLEKIERLVN--IPIVLHGASGVPKDVLEKAAKYGARLPGAAGVPDEAIRKAISLGVAKINIDTDIRLAMTAAIRQVLA--EHPEEFDPRKIM
Staphylococcus_lugdunensis      -PKLGFKEMEEIGASTG--LPLVLHGGT--------------------GIPTKDIQKAIPYGTAKINVNTENQIASAKAVRDVLN--KDQDVYDPRKYL
Staphylococcus_aureus           -PKLGFKEMEEIGLSTG--LPLVLHGGT--------------------GIPTKDIQKAIPFGTAKINVNTENQIASAKAVRDVLN--NDKEVYDPRKYL
Listeria_grayi                  -PKLGFDEMKEISELTG--APLVLHGGS--------------------GIPEHQIKKAIELGHSKINVNTECQIVWTAAVREKLA--TDDKVYDPRKVI
```

```
Brevibacillus_brevis                -PKIRYDIIEKVASNIG--APIVLHGGS---------------------GVPDEAIIESIRLGVGKINVNTESQVACTETVRKVLA--AKPNEIDPRKYL
Kyrpidia_tusciae                    -PQLDFDRLATVRDLCK--LPLVLHGGS---------------------GIPDEDIRKAISLGVAKINVNTENQEAFTAKVREIFA--ADREVYDPRKYL
Halanaerobium_praevalens            -PELDFERLDKIKKLIE--MPVVLHGAS---------------------GISAADLKTGVKYGVNKVNVNTDFQQVFTAKVKEIFT--EKPKLYDPRKYC
Halanaerobium_hydrogeniformans      -PELDFERLKTIKKLID--MPVVLHGAS---------------------GISAEDLNTAVGYGVNKVNVNTDFQQSFTAKIKEVFA--EKPELYDPRKYC
Halothermothrix_orenii              -PELDFDRLTDIKSKLD--MPLVLHGAS---------------------GVPTEDVEKAVKLGINKVNVNTDFQQAFTERIKEIFD--EKPDLYDPRKYC
Thermoanaerobacter_wiegelii         -VKLDFERLKEIASLVD--IPLVLHGAS---------------------GVPDELVKKAIALGICKLNIATELKIPFTNAIKEVFK--NNPDESDPRKFL
Helicobacter_pylori                 -PKLDFERLQEVKRLTN--IPLVLHGASTIPDDVRKSYLDAGGDLKGSKGVPFEFLQESVKGGINKVNTDTDLRIAFIAEVRKVAN--EDKSQFDLRKFF
Helicobacter_acinonychis            -PKLDFERLQEVKRLTN--IPLVLHGASAIPDNVRKSYLDAGGDLKGSRGVPFEFLQESVKGGINKVNTDTDLRIAFIAEVRKVAN--EDKSQFDLRKFF
Thiovulum_sp                        -PKLDFERLQDVKKVTN--IPLVLHGASSIPENVRQEFEGTGGDLKGSKGVPFEFLTEAIKGGINKVNTDTDLRIAFMSQVRKVAN--ENPSEFDLRKFF
Fervidobacterium_nodosum            -AKLDFERLQKVKKLTG--IPLVLHGASSVLPEYVKLAEEYGADLGGAKGVPEEELKKCVQFGINKVNTDTDLRIAFIAGMRKFLK--ENPKEFDPRHYL
Thermosipho_melanesiensis           -AKLDFERLKRVKELTK--IPLVLHGASSVPTKFVELAEKYGANLGGAKGVPQEDIKKCVELGINKVNTDTDLRIAFLAGLRKHLS--ENEKEFDPRKYF
Marinitoga_piezophila               -AKIDYDRIKKVKEYTK--IPLVLHGASSVVPEFVEIAEKHGADFGGAKGVPADILREAVKCGINKVNTDTDLRIAFVAGLREFLN--NNPKEFDPRKYF
Mesotoga_prima                      -ARLDFARLKKVKDLTG--LPLVLHGASSVPEDIKKLAETYGADFKGAKGVPGEILAESVKFGINKVNTDTDLRMAFIASLREFLS--KNPGEFDPRKYF
Aquifex_aeolicus                    -PKLDFERLKKVKELTG--IALVLHGASGVYEEYVEKINKYGGDIKGAKGVPDEELKKAVELGINKVNTDTDLRLAFISKLRELMA--LEKKVIDPRKFL
Truepera_radiovictrix               -PFIDHQRIQEIAARIP--NPLVMHGASGVPNALVERFRASGGELQYASGIHDEDVQKAIEQGIAKINTDTDLRLAFTTRVREVLR--QKPGEFDPRKIL
Thermus_thermophilus                -PFIDHPRLARIAELVP--APLVLHGASAVPQELVERFRAAGGEIGEASGIHPEDIKKAISLGIAKINTDTDLRLAFTALVRETLG--KNPKEFDPRKYL
Meiothermus_silvanus                -PFIDHERIKAIAAAIP--NPLVMHGASSVPQSLVDAFRAAGGEIGEAAGIHPEDIQKGIKAGIAKINTDTDLRLGFVAKIRQILK--DNPKEFDPRKFL
Deinococcus_deserti                 -PFIDHERIEKIEKLLG--IPLVAHGSSGVPAEIVQRFRDSGGEIGDAFGIADEDLQRATQHGIAKVNVDTDLRLAMTVGIREILK--NSPKEFDPRKIM
Meiothermus_ruber                   -PYIDHARLEQIAAKVS--LPLVLHGSSGVPQWLKDKMAASGADLGDPTGIHDEDVKKAIPNGIAKINIDTDLRLALTAGIREVVV--GNPREFDPRKIL
Thermus_scotoductus                 -PYIDHKRLEEISKRVS--IPLVLHGASGVPTWLKEKLLATGAELKEATGIHDEDIKKAIPNGIAKINIDTDLRLAMTLGIREVVV--GNPKEFDPRKII
Giardia_lamblia                     --RLAIDRVKTISD-LT-GIPLVMHGSSSVPKDVKDMINKYGGKMPDAVGVPIESIVHAIGEGVCKINVDSDSRMAMTGAIRKVFV--EHPEKFDPRDYL
Bacterium_phylotype_RsD17           ---LAFDVLKEIRA-LI-DIPIVLHGASSVPKELTGEVNKYGGKMPGATGVPMSNLQEAIKLGVSKINVDTDGRLALTAAIRKVFT--ESPEKFDPRDYL
Eikenella_corrodens                 -DVLRIDRIKEIHAALP-NTHIVMHGSSSVPQEWLKVINEHGGGIGETYGVPVEEIVEGIKHGVRKVNIDTDLRLASTGAIRKFLA--ENPAEFDPRKYL
Kingella_kingae                     -EVLRIDRIKEIHAALP-NTHIVMHGSSSVPQEWLKIINEYGGAIGETYGVPVEEIVEGIKHGVRKVNIDTDLRLASTGAIRQFMA--ENPAEFDPRKYL
Neisseria_shayeganii                -DVLRIDRIKEIHAALP-NTHLVMHGSSSVPQDWLAVINEYGGNIGETYGVPVEEIVEGIKHGVRKVNIDTDLRLASTGAIRRYLA--QHPAEFDPRKYL
Laribacter_hongkongensis            -DVLRIDRIKEIHARIP-NTHLVMHGSSSVPQEWLKVINEFGGEIPETYGVPVEEIVEGIRHGVRKVNIDTDLRLASTGAVRRFLA--QHPAEFDPRKFL
Acinetobacter_baumannii             -DILAIDRIKEIHVALP-NTHLVMHGSSSVPQEWLKVINEFGGNIGDTYGVPVEQLVEAIKHGVRKINIDTDLRLASTGAIRRFMA--ENPAEFDPRKYF
Acinetobacter_sp                    -DILAIDRIKEIHAALP-NTHLVMHGSSSVPQDWLKIINEHGGQIGETYGVPLEQLVEAIKHGVRKINIDTDLRLASTGAIRSFMS--ENPAEFDPRKYL
Marinomonas_mediterranea            -DILDIDRISTIAEKLP-NTHIVLHGSSSVPQDWLSVINEYGGEIPETYGVPVEEIVKAIRFGVRKVNIDTDLRLAATGAIRRSLA--EHKGNFDPRKYL
Oceanospirillum_sp                  -DILAIDRIKAINERIP-GTHLVMHGSSSVPQEWLAVINEFGGEIPETYGVPVEQIVEGIKYGVRKVNIDTDLRLASTGAIRRFLA--ENPAEFDPRKYL
Marinobacterium_stanieri            -DILAIDRIKAIHERIP-TTHLVMHGSSSVPQEWLAVINEFGGEIPETYGVPVEQIVEGIKYGVRKVNIDTDLRLASTGAVRRFLA--ENPAEFDPRKFL
Oceanobacter_sp                     -DILAIDRIKEIHARIP-DTHLVMHGSSSVPQDWLAIINEFGGEIPETYGVPVEQIVEGIKYGVRKVNIDTDLRLASTGAVRRFLA--ENPAEFDPRKYL
Colwellia_psychrerythraea           -DILAIDRIKAIHQRIP-HTHLVMHGSSSVPQDWLAVINEFGGKIPETYGVPVEQIQHGIKNGVRKINIDTDLRLAATGSIRRFLA--ENPSEFDPRKFL
Hahella_chejuensis                  -DILAIDRIKAIHERIP-TTHLVMHGSSSVPQDWLAIINEFGGEIAETYGVPVEEIQEGIKYGVRKVNIDTDLRLASTGAIRRFMG--QNKSEFDPRKYL
Alishewanella_aestuarii             -DILAIDRIKAIHERIP-DTHLVMHGSSSVPQDWLEIINEYGGAIPETYGVPVEQIQQGIKYGVRKVNIDTDLRLASTGAIRRYLA--KHPAEFDPRKYL
Alishewanella_jeotgali              -DILAIDRIKAIHERIP-DTHLVMHGSSSVPQDWLEIINEYGGAIPETYGVPVEQIQQGIKYGVRKVNIDTDLRLASTGAIRRYLA--KHPAEFDPRKYL
Rheinheimera_nanhaiensis            -DILAIDRIKAIHERIP-DTHLVMHGSSSVPQDWLQIINEYGGQIPETYGVPVEQIQQGIKYGVRKVNIDTDLRLASTGAIRRYLA--KHPAEFDPRKYL
Congregibacter_litoralis            -DILAIDRIKEINARIP-GTHLVMHGSSSVPQDWLEIINEYGGEIPETYGVPVEQIQEGIRHGVRKVNIDTDLRLASTGAVRRFLA--HNPAEFDPRKYL
Pseudoalteromonas_marina            -DILAIDRIKAIHARIP-NTHLVMHGSSSVPQEWLEIINQYGGEIPETYGVPVEQIVEGIKHGVRKVNIDTDLRLASTGAIRRHMA--LNPSNFDPRKYL
Alteromonadales_bacterium           -DILAIDRIKAIHARIP-NTHLVMHGSSSVPQEWLEIINQYGGEIPETYGVPVEQIVEGIKHGVRKVNIDTDLRLASTGAIRRHMA--LNPSNFDPRKYL
Idiomarina_baltica                  -DILSIDRIKEIHRRIP-NTHLVMHGSSSVPQDWLKIINEFGGEIPETYGVPVEQIKEGIKNGVRKVNIDTDLRLASTGAVRRHLA--ENPSNFDPRKYL
```

```
Shewanella_baltica                  -DVLRIDRIKEIHARIP-NTHLVMHGSSSVPQEWLQIINQYGGQIPETYGVPLEEIVEGIKHGVRKVNIDTDLRLASTGAVRKFLA--EHPSEFDPRKFL
Shewanella_violacea                 -EVLRIDRIKEIHARIP-NTHLVMHGSSSVPQEWLKVINEYGGEIPETYGVPLEEIVEGIKHGVRKVNIDTDLRLASTGAVRKFLA--ENPSEFDPRKFL
Ferrimonas_balearica                -DVLRIDRIKEIHARIP-DTHLVMHGSSSVPQEWLEVINRFGGEIPETYGVPVEEIVEGIKYGVRKVNIDTDLRLASTGAVRKFLA--ENPSEFDPRKFL
Pseudomonas_mendocina               -DVLSIERIKEIHKRIP-NTHLVMHGSSSVPQEWLKVINEFGGDIKETYGVPVEEIVEGIKHGVRKVNIDTDLRLASTGAIRRMMA--EHPSEFDPRKFF
Pseudomonas_fulva                   -DVLSIERIKEIHKRIP-NTHLVMHGSSSVPQDWLAIINEYGGDIKETYGVPVEEIVEGIKYGVRKVNIDTDLRLASTGAIRRFMA--QNPAEFDPRKYF
Azotobacter_vinelandii              -DILAIDRIKEIHKRIP-DTHLVMHGSSSVPQDWLKVINEYGGDIKETYGVPVEEIVEGIKYGVRKVNIDTDLRLASTGAIRRFMG--QSPSEFDPRKYL
Halomonas_sp                        -DTLSIQRIKEIHARIP-DTHLVMHGSSSVPQEWLAVINEFGGEIPETYGVPVEEIVEGIKYGVRKVNIDTDLRLASTGAVRRFLA--KHPGEFDPRKYL
Halomonas_boliviensis               -DTLSIQRIKEIHARIP-DTHLVMHGSSSVPQEWLEVINQYGGEIPETYGVPVEEIVEGIKHGVRKVNIDTDLRLASTGAVRRFLA--ENPSEFDPRKFL
Chromohalobacter_salexigens         -DILSIQRIKEIHARIP-DTHLVMHGSSSVPQEWLEVINAHGGEIPETYGVPVEEIVEGIKHGVRKVNIDTDLRLASTGAVRRFLA--ENPSEFDPRKFL
Marinobacter_algicola               -DILAIEQIKAIHKRIP-DTHLVMHGSSSVPQEWLKIINEYGGEIPETYGVPVEEIVEGIKHGVRKVNIDTDLRLASTGATRRFLA--QNPAEFDPRKFL
Marinobacter_aquaeolei              -DILAIDQIKAIHARIP-DTHLVMHGSSSVPQEWLKVINEFGGEIPETYGVPVEEIVEGIKHGVRKVNIDTDLRLASTGAVRRFMA--QNPAEFDPRKFL
Thiocystis_violascens               -DILAIERIKEIHKRIP-TTHLVMHGSSSVPQDWLAIINQYGGAMGETYGVPVEEIVEGIKHGVRKVNIDTDLRMSSTGAIRKFLA--ENPKEFDPRKYF
Thiocapsa_marina                    -DILAIERIKEIHKRIP-NTHLVMHGSSSVPQDWLAIINQYGGDMGETYGVPVEEIVEGIKNGVRKVNIDTDLRMSSTGAIRKFLA--ENPKEFDPRKYF
Allochromatium_vinosum              -DILAIERIKEIHTRIP-TVHLVMHGSSSVPQEWLKIINEHGGDMGETYGVPVEEIVEGIKHGVRKVNIDTDLRMASTGAIRKSLH--DDRKNFDPRKYF
Thiorhodococcus_drewsii             -DILAIERIKEIHARIP-NTHLVMHGSSSVPQEWLKIIDEFGGDMGETYGVPVEEIAEGIKHGVRKVNIDTDLRMASTGAIRKHLA--ENRKNFDPRKYF
Marichromatium_purpuratum           -DILAISRIKEIHQRIP-NTHLVMHGSSSVPQEWLAIINEFGGDMGETYGVPVEEIVEGIKHGVRKVNIDTDLRMASTGAIRKHLA--ENRANFDPRKYY
Thiomonas_intermedia                -DILAIGRIKEIHARIP-NTHLVMHGSSSVPQEWLKIINSYGGDMGETYGVPVEEIVEGIKHGVRKVNIDTDLRMASTGATRKFLA--EHPKEFDPRKFL
Francisella_novicida                -DVLSIKRVKEIHARIP-DTHLVMHGSSSVPQDWLEVINTYGGAMGETYGVPVEEIVEAIKYGVRKINIDTDLRMAATGAIRRFLA--ENPAEFDPRKYN
Francisella_noatunensis             -DVLSIKRVKEIHARIP-DTHLVMHGSSSVPQDWLEVINTYGGAMGETYGVPVEEIVEAIKYGVRKINIDTDLRMAATGAIRRFMA--ENPAEFDPRKYN
Kangiella_koreensis                 -DVLAIERIKAIHERIP-DTHLVMHGSSSVPQDWLATINEYGGDLGETYGVPVEEIQEGIKHGVRKVNIDTDLRLAATGAVRKFLK--HNPKEFDPRKYN
Coxiella_burnetii                   -EVLVIDRIKAIHERLP-DTHLVMHGSSSVPQEWLQIINEYSGEIPETYGVPVEEIQKGIRHGVRKVNIDTDLRLASTGAIRRYLA--KNPSQFDPRKYL
Thiothrix_nivea                     -QVLRIDRVKEIHARIP-SVHLVMHGSSSVPEEWLEIINNFGGDMGQTYGVPVSEIVEGIKNGVRKVNIDTDLRMASTGSIRRHLE--QNKSNFDPRKFL
Thiobacillus_denitrificans          -KVLRIDRVKEIHARIP-NVHLVMHGSSSVPEDWLAIINSYGGDMGQTYGVPVEEIVEGIKNGVRKVNIDTDLRMASTGAIRKHLA--ENKSNFDPRKFL
Thioalkalimicrobium_cyclicum        -DVLKIDQIKKIHARIP-TTHLVMHGSSSVPEEWLSIINNYGGDMGQTYGVPVEAIVEGIKYGVRKVNIDTDLRMASTGAIRKHLA--ENTSNFDPRKFF
Halothiobacillus_neapolitanus       -KVLRIDRVKEIHARIP-NTHLVMHGSSSVPEDWLEIINNYGGDMGQTYGVPVEEIVEGIKHGVRKVNIDTDLRMASTGAIRKHLH--DNPANFDPRKFF
Nitrosococcus_watsonii              -DILAIERIKEIHARIP-DTHLVMHGSSSVPQDWLKIIHEFGGDIGETYGVPVKEIQEGIRHGVRKVNVDTDLRLASTGAIRKNLA--ENPKNFDPRKYL
Halorhodospira_halophila            -DILAISRIKEIHQRLP-DTHLVMHGSSQVPQEWLELINRFGGEIPETYGVPVEEVQEGIRNGVRKVNIDTDLRLASTGAVRKHLA--ENPSNFDPRKFL
Alkalilimnicola_ehrlichii           -DILAIERIKEIHARIP-DTHLVMHGSSSVPQEWLEIINQFGGEMPETYGVPVEEIQEGIRHGVRKVNVDTDLRLAATGAVRRHLA--ENPSNFDPRKFL
Rubrivivax_benzoatilyticus          -DILAIDRIAAIHAKIP-NTHLVMHGSSSVPQEWLQIIRQHGGSIRETYGVPVEEIVRGIRHGVRKINIDTDIRLAMTGAMRKVMA--EKPEEFDPRAFL
Rubrivivax_gelatinosus              -DILAIDRIAAIHAKIP-NTHLVMHGSSSVPQEWLAIIRQHGGAIRETYGVPVEEIVRGIRHGVRKINIDTDIRLAMTGAMRKVMA--EKPEEFDPRAFL
Hydrocarboniphaga_effusa            -EILAIDRVKAIHARIP-NTHLVMHGSSSVPQEWLTVIREFGGRIKETYGVPVEEIRLGIRHGVRKVNIDTDIRLAMTGAMRQKMG--QKPDEFDPRAFL
Dechloromonas_aromatica             -EILAIDRIKAIHARIP-NTHLVMHGSSSVPQEWLAVIRQYGGAIKETYGVPIGEIVEGIRHGVRKVNIDTDIRLAMTGAMRKAMA--EKPEEFDPRAFW
Oxalobacteraceae_bacterium          -DILAIDRIRAIHRRIP-ETHLVMHGSSSVPQEWLDIIRSHGGTIRETYGVPVEEILVGIKNGVRKINIDTDIRLSMTGAMRRTMA--EHPEEFDPRRFF
Leptothrix_cholodnii                -DILAIDRIARIHAAVP-DTHLVMHGSSSVPQEWLAIIRQYGGDIKQTYGVPVDEIVRGIRSGVRKVNIDTDIRLAMTGAMRQVFA--TQPGEFDPRKAL
Methylovorus_glucosetrophus         -DILAIERIREIHARLP-NTHLVMHGSSSVPQEWLQLINAHGGEIPETYGVPVEEIVQGIRHGVRKVNIDTDLRMAFIGSAREFLHGGQHNRELDPRKLL
Methylobacillus_flagellatus         -ETLAISRLREIHARLP-NTHLVMHGSSSVPQEWLQVINEYGGEIPETYGVPVEEIVKGIRSGVRKVNIDTDLRLAFTGALRRFLGQPEHTAEFDPRKIL
Methylotenera_versatilis            -KTLAISRIKEIHARIP-NTHLVMHGSSSVPQEWLQIINEFGGDMGETYGVPVEEIVEGIKHGVRKINIDTDLRMAYTGSIRKFFT--EKRKEFDPRKFL
Nitrosospira_multiformis            -DILAIERIKEIHARIP-NTHLVMHGSSSVPQDWLDIIREYGGEMKETYGVPVEEIQEGIKYGVRKVNIDTDIRLAMTGAIRRHLA--KNKSEFDPRKYL
Gallionella_capsiferriformans       -AVLRIDRIREIHERLP-NTHLVMHGSSSVPQEWLKIINEFGGTMGETYGVPVEEIVEGIKNGVRKVNIDTDLRMASTGATRRYLA--QNTKDFDPRKFL
Methylomirabilis_oxyfera            -DVLVMERVKEIHARIP-DTHLVMHGSSSVPQEWLKIIREFGGDMPQTYGVPIEEIQLGIKHGVRKVNIDTDLRLAATGAIRQDLA--QNKKNFDPRKFL
Acidithiobacillus_caldus            -KVLRIDRVKEIHARIP-DTHLVMHGSSSVPQEWLKVIHEFGGDIGETYGVPVEEIQEGIKHGVRKVNIDTDLRLAATGAVRKSLY--TNPKNFDPRKFL
```

```
Magnetospirillum_magneticum            -EVLAIKRIKEIHARIP-NTHLVMHGSSSVPQDLLKIVNDYGGALGETYGVPVGEIVEGIKHGVRKINIDTDLRLAVTGCIRKYFH--DNPKKFDPRDYL
Magnetospirillum_magnetotactic         -EVLAIKRIKEIHARIP-NTHLVMHGSSSVPQDLLKIVNDYGGALGQTYGVPVEEIVEGIKHGVRKVNIDTDLRLAVTGCIRKYFH--DNPKKFDPRDYL
Phaeospirillum_molischianum            -DVLAISRVKEIHARIP-NTHLVMHGSSSVPQDWLKIINDNGGDLGETYGVPVEEIVEAIKHGVRKVNIDTDLRMASTGAIRKFFN--DEPKKFDPRDYF
Aromatoleum_aromaticum                 -DILAIDRVKAIHARIP-NTHLVMHGSSSVPQELLEIIRQHGGDMKETYGVPVEEIQTAIGFGVRKINIDTDIRLAMTGAVRKFMA--ENPSKFDPREFL
Thauera_sp                             -DILAIERVKAIHARIP-NTHLVMHGSSSVPQELLEIIRQYGGDMKETYGVPVEEIVQGIKYGVRKINIDTDIRLAMTGAIRKFMF--ENPSKFDPREYN
Azoarcus_sp                            -DILAIDRIKAIHARLP-NTHLVMHGSSSVPQDLLEIIRQYGGDMKETYGVPVEEIVEGIKYGVRKINIDTDIRLAMTGAIRKFFV--ENPSKFDPREFL
Dechlorosoma_suillum                   -DILAIDRIKEIHARIP-NTHLVMHGSSSVPQDLLAEIREFGGDMKETYGVPVEEIVNGIKHGVRKVNIDTDIRLAMTGAVRRYLF--ENPSKFDPRDFL
Accumulibacter_phosphatis              -DILAIQRIKEIHERIP-NCHLVMHGSSSVPQELLAEIREFGGDMKETYGVPVEEIVTGIKHGVRKVNIDTDIRLAMTGAIRRYFA--ENPSKFDPRDYL
Herbaspirillum_sp                      -DILAIDRIKEIHARIP-NTHLVMHGSSSVPQELLAEIREFGGNMKETYGVPVEEIQEGIKHGVRKINIDTDIRLAMTGAIRRFFI--ENPEKFDPRDYL
Herbaspirillum_seropedicae             -DILAIDRIKEIHARIP-NTHLVMHGSSSVPQELLAEIREFGGDMKETYGVPVEEIQEGIKHGVRKINIDTDIRLAMTGAIRRYLF--ENPSKFDPRDYL
Collimonas_fungivorans                 -DILAIERIKEIHTRIP-NTHLVMHGSSSVPQELLAEIREFGGDMKETYGVPVEEIQEGIRHGVRKINIDTDIRLAMTGAIRRYLI--ENPSKFDPRDYL
Herminiimonas_arsenicoxydans           -DILAIERIREIHLRIP-NTHLVMHGSSSVPQELLAEIREFGGDMKETYGVPVEEIQIGIKNGVRKINIDTDIRLAMTGAIRRYLM--ENPSKFDPRDYL
Polynucleobacter_necessarius           -DILAIDRIKEIHARIP-NTHLVMHGSSSVPQELLAEIREFGGDMKETYGVPVEEIQEGIKNGVRKINIDTDIRLAMTGAIRRYFI--ENPSKFDPRDYL
Janthinobacterium_sp                   -DILAIDRIKEIHARIP-NTHLVMHGSSSVPQELLAIIREFGGDMKETYGVPVEEIQEGIRHGVRKINIDTDIRLAMTAAIRKYLF--ENPSKFDPRDFL
Burkholderia_cenocepacia               -DILSIQRIKEIHARIP-NTHLVMHGSSSVPQELLAEIREFGGDMKETYGVPVEEIQEGIKHGVRKINIDTDLRLAITGAIRRYLF--ENPGKFDPRDYL
Oxalobacter_formigenes                 -DILAIDRLKEIHARLP-NTHLVMHGSSSVPQELLALIREYGGDIRETYGVPVEELQESIKFGVRKINIDTDIRLAMTAAIRRYLM--QNPSHFDPREYL
Ralstonia_eutropha                     -DILAINRIKEIHARIP-NTHLVMHGSSSVPQELLEEIRKFGGDMKETYGVPVEEIQEAIKYGVRKINIDTDIRLAMTGAIRRFFV--ENPSKFDPREYL
Cupriavidus_taiwanensis                -DILAINRIKEIHARIP-NTHLVMHGSSSVPQELLEEIRKFGGDMKETYGVPVEEIQEAIKYGVRKINIDTDIRLAMTGAIRRFFV--ENPSKFDPREYL
Cupriavidus_necator                    -DILAISRIKEIHARIP-NTHLVMHGSSSVPQELLEEIRKFGGDMKETYGVPVEEIQEAIKYGVRKINIDTDIRLAMTGAIRRFFA--ENPSKFDPREYL
Acidovorax_citrulli                    -DILAISRVKEIHARIP-NTHLVMHGSSSVPQELLAVINQYGGKMKETYGVPVKEIQEAIKYGVRKINIDTDIRLAMTGAVRKFLA--ENPDKFDAREWL
Comamonas_testosteroni                 -DILAISRVKEIHARIP-NTHLVMHGSSSVPQELLAIINQYGGKMKETYGVPVEEIQEAIKYGVRKINIDTDIRLAMTGAVRKFLA--ENPDKFDAREWL
Acidovorax_radicis                     -DILAISRVKEIHQRIP-NTHLVMHGSSSVPAELLAIINQYGGKMKETYGVPVEEIQEAIKHGVRKINIDTDIRLAMTGAVRKFLF--ENPDKFDAREWL
Limnobacter_sp                         -DILAISRVKEIHARIP-NTHLVMHGSSSVPAELLAIINQYGGKMKETYGVPVEEIQEAIKYGVRKINIDTDIRLAMTGAVRKFLF--ENPDKFDAREWL
Hylemonella_gracilis                   -DILAISRVKEIHARIP-NTHLVMHGSSSVPQELLALINQYGGKMKETYGVPVSEIQEAIKHGVRKINIDTDIRLAMTGAVRKFLA--ENPDKFDAREWL
Methyloversatilis_universalis          -DILAISRVKEIHARIP-NTHLVMHGSSSVPQDLLDIINQYGGKMKQTYGVPVPEIQEAIKYGVRKINIDTDIRMAMTGAVRKFLT--ENPEKFDMREWM
Alicycliphilus_denitrificans           -DVLAISRVKEIHARIP-NTHLVMHGSSSVPQDLLDIINQYGGKMKQTYGVPVKEIQEAIKHGVRKINIDTDIRMAMTGAVRKFQA--ENPDKFDMREWM
Delftia_sp                             -DILSIQRVKEIHARLP-NTHLVMHGSSSVPQELLEIIRQYGGAMKETYGVPVEEIQEAIKHGVRKINIDTDIRLAMTGAVRKFLA--ENPEKFDAREWL
Delftia_acidovorans                    -DILSIQRVKEIHARLP-NTHLVMHGSSSVPQELLEIIRQYGGAMKETYGVPVEEIQEAIKHGVRKINIDTDIRLAMTGAVRKFLA--ENPEKFDAREWL
Ralstonia_pickettii                    -DILAISRVKEIHARIP-NTHLVMHGSSSVPQELLAIINQYGGKMKETYGVPVEEIQEAIKYGVRKINIDTDIRLAMTGAVRKFLA--ENPDKFDAREWL
Variovorax_paradoxus                   -DILAIDRIKEIHRRIP-NTHLVMHGSSSVPQELLAIIRQYGGNMKETYGVPVKEIQEAIKHGVRKINIDTDIRLAMTGAVRKFLA--ENPEKFDAREWL
Hydrogenophaga_sp                      -DILAIERIAEIHRRLP-NTHLVMHGSSSVPQELLAQINQYGGKMKETYGVPVEEIQKAIKFGVRKINIDTDIRLAMTGAVRKFLT--ENPDKFDAREWL
Ramlibacter_tataouinensis              -DILAIDRIKEIHRRIP-NTHLVMHGSSSVPQELLAVIREYGGKMKETYGVPVEEIQEAIKYGVRKINIDTDIRLAMTGAVRKFLA--GNPDKFDMREWM
Polaromonas_naphthalenivorans          -DILAIERVKEIHRRIP-NTHLVMHGSSSVPQDLLAIINQYGGKMKETYGVPVEEIQKAIQFGVRKINIDTDIRLAMTGAVRKFLA--ENPDKFDAREWL
Methylibium_petroleiphilum             -DILAIKRIKEINARIP-NTHLVMHGSSSVPQDLLAEIRKYGGDMKETYGVPVSEIQEAIKFGVRKINIDTDIRLAMTAAIRKFFV--ENPGKFDPREYL
Achromobacter_xylosoxidans             -DILSISRIKEIHARLP-NTHLVMHGSSSVPQELLAEIREFGGNMKETYGVPVEEIQEAIKYGVRKINIDTDIRLAMTGAIRRFFA--ENPEKFDPREYL
Achromobacter_arsenitoxydans           -DILSISRIKEIHARLP-NTHLVMHGSSSVPQELLAEIREFGGNMKETYGVPVEEIQEAIKYGVRKINIDTDIRLAMTGAIRRFFA--ENPEKFDPREYL
Bordetella_petrii                      -DILSISRIKEIHARLP-NTHLVMHGSSSVPQELLAEIREFGGDMKETYGVPVEEIQEAIKFGVRKVNIDTDIRLAMTGAIRRFFA--ENPSKFDPREYL
Bordetella_pertussis                   -DILSIARIKEIHARLP-NTHLVMHGSSSVPQDLLAEIREFGGDMKETYGVPVEEIQEAIKFGVRKINIDTDIRLAMTGAIRRFFA--ENPSKFDPREYL
Advenella_kashmirensis                 -DILSINRIKEIHARLP-NTHLVMHGSSSVPQELLAEIREFGGDMKETYGVPVEEIQEAIKFGVRKVNIDTDIRLAMTGAIRRFLG--ENPGKFDPREYN
Pusillimonas_sp                        -DILSISRIKEIHKRLP-NTHLVMHGSSSVPQDLLAEIREFGGDMKETYGVPVEEIQEAIKFGVRKVNIDTDIRLAMTGAIRRYMG--ENPSKFDPREYL
Alcaligenes_faecalis                   -DILSIERVKEIHRRLP-NTHLVMHGSSSVPQELLAEIRQFGGNMKETYGVPVEEIQEAIKYGVRKVNIDTDIRLAMTAAIRRFLF--ENPEKFDPREYL
```

```
Taylorella_equigenitalis            -DILSIQRIKEINARLP-NTHIVMHGSSSVPQEYLEQIRMYGGDMKETYGVPVEEIQEAIKYGVRKVNIDTDIRLAMTAAVRKFLY--ENPSKFDPREFN
Parasutterella_excrementihomin      -DILSIQRVKEIHARIP-NTHLVMHGSSSVPQEYLEEIRKYGGQFRTTYGVPVEEIVEAIKYGVRKVNIDTDIRLAMTAAIRKYFV--ENPEAFDPRAYL
Burkholderiales_bacterium           -DILSIQRVKEIHARIP-NTHLVMHGSSSVPQEYLEEIRKYGGQFRTTYGVPVEEIVEAIKYGVRKVNIDTDIRLAMTAAIRKYFV--ENPEAFDPRAYL
Sutterella_wadsworthensis           -EILSIDRVKEIHARLP-NTHLVMHGSSSVPQEYLAEIREFGGDMRETYGVPVEEIQKAIKHGVRKVNVDTDIRLAMTAAIRRYLV--ENPSGFDPRAYL
Xanthobacter_autotrophicus          -DILAIDRVKAIHQRIP-TTHLVMHGSSSVPQDLLEEIRTYGGDIPETYGVPVEEIQEGIRYGVRKVNIDTDIRLAMTAGMRRVGA--KNKSEFDPRKFL
Magnetococcus_marinus               -DILAIGRIAEINARIP-NTHLVMHGSSSVPQELLAIINEFGGEIPETYGVPVEEIVRGIQHGVRKINVDTDLRLAMTGAIRKYFH--KNPSAFDPRGYN
Nostoc_punctiforme                  -EILAISRIEEIHRRLP-NTHLVMHGSSSVPEDLLALINQYGGAIPETYGVPVEEIQKGIKSGVRKVNIDTDNRLAITAAVREALA--KKPEEFDPRHFL
Anabaena_variabilis                 -EILAISRIEEIHRRLP-NTHLVMHGSSSVPEDLIALINEYGGAIPETYGVPVEEIQKGIKSGVRKVNIDTDNRLAITAAVREALA--KNPKEFDPRHFL
Microcystis_aeruginosa              -EILAISRIEEIHNRLP-NTHLVMHGSSSVPEDLLELINQFGGAIPETYGVPVEEIQKGIKSGVRKINIDTDCRLAITAAVREALF--SNPKEFDPRFFL
Lyngbya_sp                          -EVLAISRIEEIHRRLP-NTHLVMHGSSSVPEDLIAIINQYGGKIPETYGVPVEEIQKGIKSGVRKVNIDTDNRLAITAAFREAAA--KDPANFDPRHFL
Arthrospira_platensis               -EILAISRIEEIHSRLP-NTHLVMHGSSSVPEDLIALINQYGGQIPETYGVPVEEIQKGIKSGVRKVNIDTDNRLAITAAIREAAA--KDPSNFDPRHFM
Oscillatoria_sp                     -EVLAISRIEEIHRRLP-NTHLVMHGSSSVPEDLLALINQYGGKIRETYGVPVEEIQKGIQSGVRKINIDTDNRLAITAAVREALA--AKPEEFDPRHFM
Cyanothece_sp                       -EILAISRIEEIHKRLP-NTHLVMHGSSSVPQEWLDIINQYGGQIPETYGVPVEEIQKGIKSGVRKVNIDTDNRLAITAAIREAAA--KDPANFDPRHFM
Synechococcus_elongatus             -EILAISRIEEIHRRLP-NTHLVMHGSSSVPQELLELINEYGGQIPETYGVPVEEIQKAIKLGVRKINIDTDNRLAFTAAVREAAA--KDPSNFDPRHFN
Prochlorococcus_marinus             -EVLAISRIAEIHKAIP-NTHLVMHGSSSVPQEWLDMINKFGGAIPETYGVPVEEIQEGIRNGVRKVNIDTDNRLAFTAAVREAAA--ADPTNFDPRHFN
Paulinella_chromatophora            -EVLAISRIAEIHKAIP-NTHLVMHGSSSVPQEWLEMINKYGGAIPETYGVPVEEIQEGIRNGVRKINIDTDNRLAFTAAIREAAF--SDPANFDPRHFN
Synechococcus_sp                    -DILDMERIAEIHRRLP-NTHLVMHGSSSVPKELIDIINQYGGSIPETYGVPLEEIQRGIKNGVRKVNIDTDNRLAITAAVRRALA--ENPKDFDPRSFL
Gloeobacter_violaceus               -AILAIDRIREIHRRLP-NTHLVMHGSSSVPQDLQDLINAHGGAIPQTWGVPVTEIQEGIRNGVRKVNVDTDNRLAITAAVREALA--KEPAEFDPRKFL
Sinorhizobium_meliloti              -EILSIETIAKINKRLP-NTHLVMHGSSSVPADLQELFNAYGGKMKKTWGVPVSEIQKAIPLGVRKVNIDTDLRLAFTGEIRKHHI--EHPDNFDPRNYL
Stappia_aggregata                   -EILAMHVIEEIHRRLP-ETHLVMHGSSSVPQDLQDIINQYGGQMPQTWGVPVEEIQRGIKNGVRKVNIDTDNRMAMTGQIRKILS--ENPGEFDPRKYL
Labrenzia_alexandrii                -DILAMHVIEEIHRRLP-DTHLVMHGSSSVPQDLQDIINQYGGEMPQTWGVPVEEIQRGIKNGVRKVNIDTDNRMAITGQIRKILQ--DNPGEFDPRKYL
Polymorphum_gilvum                  -EILAMHVIEEIHRRLP-ETHLVMHGSSSVPQDLQDIINKYGGKMPQTWGVPVEEIQRGIKNGVRKINIDTDNRMAMTGQIRKILA--ENPGEFDPRKYL
Bradyrhizobium_sp                   -DILAMNVIEEIHRKLP-NTHLVMHGSSSVPQDLQDIINEFGGKMKPTWGVPVSEIQRGIKHGVRKINIDTDNRMAMTGQIRKVFK--EHPEEFDPRKYL
Agrobacterium_radiobacter           -SVLAMEVIEAIHRKLP-NTHLVMHGSSSVPEDLQEIINKYGGQMKPTWGVPVEEIQRGIKNGVRKINIDTDGRMAMTGQIRRVLQ--EDPSEFDPRKYM
Rhizobium_sp                        -SVLAMEVIEAIHRKLP-NTHLVMHGSSSVPEDLQEIINKYGGQMKPTWGVPVEEIQRGIKNGVRKINIDTDGRMAMTGQIRRVLQ--EDPSEFDPRKYM
Brucella_melitensis                 -SVLAMHVIEEIHRKLP-NTHLVMHGSSSVPEELQEIINKYGGQMKPTWGVPVEEIQRGIKHGVRKINIDTDNRMAMTGQIRRILQ--EEPSEFDPRKYL
Brucella_suis                       -SVLAMHVIEEIHRKLP-NTHLVMHGSSSVPEELQEIINKYGGQMKPTWGVPVEEIQRGIKHGVRKINIDTDNRMAMTGQIRRILQ--EEPSEFDPRKYL
Ochrobactrum_anthropi               -SVLAMNVIEEIHRKLP-NTHLVMHGSSSVPEELQEIINKYGGKMKPTWGVPVEEIQRGIKHGVRKINIDTDNRMALTGQIRRVLQ--EDPSEFDPRKYL
Sinorhizobium_fredii                -DVLAMDVIEAIHRKLP-NTHLVMHGSSSVPEELQEIFNRFGGRMKPTWGVPLEEILRGIRHGVRKVNIDTDCRMAMTGQVRRVLS--EDPTEFDPRKYL
Mesorhizobium_amorphae              -AVLAMNVIEEIHRRLP-NMHLVMHGSSSVPEDLQEIINKYGGQMKPTWGVPVEEIQRGIKHGVRKINIDTDNRMALTGAIRKVLT--ENPSEFDPRKYL
Mesorhizobium_loti                  -AVLAMNVIEEIHRRLP-NMHLVMHGSSSVPEDLQEIINKYGGQMKPTWGVPVEEIQRGIKHGVRKINIDTDNRMALTGAIRKVLT--ENPSEFDPRKYL
Methylobacterium_nodulans           -AVLAMHVIEEINRRLP-NTHLVMHGSSSVPQELQDIINQYGGEMKPTWGVPVEEIQRGIKHGVRKINIDTDNRMAMTGQIRKVLT--ENKAEFDPRKYL
Methylobacterium_radiotolerans      -EVLAMNVIEEIHRRLP-TTHLVMHGSSSVPQDLQDIINQYGGQMKPTWGVPVEEIQRGIKHGVRKINIDTDNRMAMTGQIRKVLT--ENPSEFDPRKYL
Phenylobacterium_zucineum           -EVLAMNVIEEIHQRLP-NTHLVMHGSSSVPQELQDIINQYGGQMPQTWGVPIEEIQRGIKHGVRKINIDTDNRMAITGAIRKVFA--EKPGEFDPRGYL
Caulobacter_sp                      -DVLAMHVIEEIHQRLP-NTHLVMHGSSSVPQDLQDIINQYGGAIPQTWGVPIEEIQRGIKHGVRKVNVDTDNRLAITGAIRKILA--EHPHEFDPRAYL
Caulobacter_crescentus              -DVLAMNVIEEIHRRLP-NTHLVMHGSSSVPQDLQDIINQYGGEMPQTWGVPVEEIQRGIKHGVRKINVDTDNRMAITGAIRKLLV--EKPGEFDPRAYL
Paracoccus_denitrificans            -DILAMHVIEAIHERLS-GTHLVMHGSSSVPQYLQDLINEAGGEMPQTYGVPVEEIERGIRHGVRKVNIDTDCRMAMTGQFRKVAR--ERPEEFDPRKFL
Paracoccus_sp                       -DILAMHVIEAIHERLP-GTHLVMHGSSSVPQYLQDLINEAGGEMPQTYGVPVEEIERGIRHGVRKVNIDTDCRMAMTGHFRKVAR--EAPQEFDPRKFM
Pelagibaca_bermudensis              -DILSMETIRRINEKLP-NTHLVMHGSSSVPQALQDLINANGGHMPQTYGVPVEEIERGIRMGVRKVNIDTDCRMAMTGQYRRIAQ--EMPEEFDPRKFN
Citreicella_sp                      -DILSMETIRRINEKLP-DTHLVMHGSSSVPQELQDLINANGGHMPQTYGVPVEEIERGIRMGVRKVNIDTDCRMAMTGQYRRIAQ--EKPDEFDPRKFN
Rhodopseudomonas_palustris          -DVLAMKVVEEIHRRLP-NTHLVMHGSSSVPQDLQEAFNKYGGAMPQTFGVPVEEIVRGIKHGVRKVNIDTDCRLAMTGVFRKIAT--EHKAEFDPRKFL
```

```
Starkeya_novella                   -DILAMHVIEEIHRRLP-SVHLVMHGSSSVPQALQDLFNASGGEMPQTWGVPVEEIVRGIRHGVRKVNIDTDCRLAMTAQFRKVGQ--ANRSEFDPRKFL
Azospirillum_amazonens             -DILAMDVVEAIHERLP-NTHLVMHGSSSVPQELQDLFNAYGGRMPRTWGVPVDEIVRGIRFGVRKINIDTDCRLAMAVQFRKTAT--ENPAEFDPRKFL
Rhodobacter_sphaeroides            -EILAMSVIEAIHRKLP-DTHLVMHGSSSVPQELQDIINAFGGAMPQTFGVPVEEIVRGIKMGVRKVNIDTDCRMAMTGQFRRIAQ--QTPSEFDPRKFL
Aurantimonas_manganoxydans         -DILAMNIIEEIHAKLP-NTHLVMHGSSSVPEELQAIINQYGGRMRETYGVPVDEIVRGIKHGVRKVNIDTDCRMAMTGQFRKVAM--EKPEEFDPRKFM
Octadecabacter_arcticus            -EILAMDRLQEIHARIP-RTHLVMHGSSSVPQELQDIINDAGGQIEPTWGVPVEEIQLGIKSGVRKVNVDTDLRLALTGAIRKTFA--LNPSEFDPRKYL
Sulfitobacter_sp                   -DILAMDALKKIHGKLP-DTHLVMHGSSSVPQELQEIINRYGGEMAPTWGVPLEEIELGIKHGVRKVNIDTDLRMALTGAIRKVLA--EDKGQFDPREYL
Rhodospirillum_photometricum       -DILAMNVVKAIHARLP-GTHLVMHGSSSVPQELQDIINQYGGEMPQTFGVPVEEIVEGIKHGVRKVNIDTDCRMAITGAIRKVFA--ENKAEFDPRKYL
Pirellula_staleyi                  -SVLAMDRIEAIHKKLP-NTHLVMHGSSSVPQELQDIINKYGGKMPQTWGVPVEEIQRGIKNGVRKINVDTDCRMAITGAIRKVLT--ESPDKFDPRDYL
Blastopirellula_marina             -EVLAMDRIEAIHKKLP-NTHLVMHGSSSVPQELQDIINQYGGKMKQTFGVPVEEIQRGIKSGVRKINVDTDNRMAITGAIRKVLM--ESPSEFDPRAYM
Rhodopirellula_baltica             -EVLAMDRIEAIHAKIP-NTHLVMHGSSSVPQELQDIINQFGGEMKQTYGVPVEEIQRGIKSGVRKINVDTDCRMAITGAIRKVLT--EDKAAFDPRAYL
Koribacter_versatilis              -SVLKMQRLIEIHKRLP-NTHLVMHGSSSVPKDLQDVINQYGGQLKPTWGVPVEEIQLGIRNGVRKVNVDTDNRLAITGAIRKVFI--ETPEKFDPRDYL
Solibacter_usitatus                -ETLKMKVLIEIHKKLP-NVHLVMHGSSSVPKDLQDIINQYGGKLKPTWGVPVEEIQLGIKNGVRKINVDTDNRLAITGAIRKVFI--EKPEEFDPRSYM
Anaerolinea_thermophila            -QTLRFDVLAEIQKRLP-GFPLVMHGSSSVPQEEVERINRAGGNLKGAKGVDENQFRRAAELGVTKVNIDTDGRLVWTRVHREYFR--DHPEEIDMRPIG
Diplosphaera_colitermitum          -QSLHFDVLEKIKARLP-GFPLVMHGSSSVPQDEVKRINAAGGQIKDSAGVNVEEYLPAAKLGVTKINIDTDGRLVWTRVHREFFR--DKPAEFDFRAPG
CONSERVATION                                             * **                       *               * *                          * *

                                           410       420       430       440       450       460
                                   ....|....|....|....|....|....|....|....|....|....|....|....|....|..
Chlorobium_phaeovibrioides         GPARDALMELYKHKNIN--VLGSNGKA----------------------------------------
Pelodictyon_phaeoclathratiform     GPARDALKKLYVHKIIN--VLGSDGKA----------------------------------------
Chlorobium_chlorochromatii         GPARDSLKKIYIHKNIN--VLGSNGKA----------------------------------------
Prosthecochloris_aestuarii         GPARDALKELYKHKNIN--VLGSNDKA----------------------------------------
Chlorobaculum_parvum               GPAREALKELYKHKIIN--VLGSNGKA----------------------------------------
Victivallis_vadensis               GPARTALKELYKRKNIE--VLGSAGHAQD--------------------------------------
Brachyspira_murdochii              GPARDEMKKLYIHKINN--VLGSNGKI----------------------------------------
Brachyspira_pilosicoli             GPARDEMKKLYIHKIMN--VLGSNGKI----------------------------------------
Alistipes_indistinctus             GPARDNMKKLYEHKIVS--VLGSAGKLAEE-------------------------------------
Tannerella_sp                      GPARDNMKKLYKHKIED--VLGSAGKGGCCC------------------------------------
Tannerella_forsythia               GPARDAAKALYIHKIKN--VLGSDNKL----------------------------------------
Bacteroides_intestinalis           GPARDNMEKMYMHKIIN--VLGSDGKLAE--------------------------------------
Bacteroides_uniformis              GPARDNMEKLYKHKIIN--VLGSENKLAQLD------------------------------------
Parabacteroides_merdae             GPARDNMEKLYKHKIIN--VLGSNDKL----------------------------------------
Parabacteroides_sp                 GPARDNMEKLYKHKIIN--VLGSNDKL----------------------------------------
Capnocytophaga_sp                  GPARDNMKKLYEHKIVN--VLGSDNKL----------------------------------------
Paraprevotella_clara               GPARDNMKKLYEHKIVN--VLGSDNKL----------------------------------------
Paraprevotella_xylaniphila         GPARDNMKKLYEHKIVN--VLGSDNKL----------------------------------------
Bacteroides_salanitronis           GPARDEMEKMYKHKILN--VLGSDGKLAQ--------------------------------------
Dysgonomonas_gadei                 GPARDNMKKLYKHKIEA--VLGSAGKA----------------------------------------
Dysgonomonas_mossii                GPARDNMKKLYKHKIEA--VLGSAGKAE---------------------------------------
Paludibacter_propionicigenes       GPARDELKKLYSHKTVN--VLGSAGKA----------------------------------------
Alistipes_sp                       GPARDELKKLYMHKCES--VLGSAGKA----------------------------------------
```

```
Odoribacter_laneus                  GPARDSLKELYKHKLVN--VLGSANTVK---------------------------------------
Porphyromonas_endodontalis          GPARDSLKELYKHKICD--VLGSNDKL----------------------------------------
Candidatus_Azobacteroides           GPARELLKALYKHKVVH--VLGSAGKA----------------------------------------
Sphaerochaeta_pleomorpha            GPARAELKKMYMHKNID--VLGSAGQA----------------------------------------
Sphaerochaeta_coccoides             GPARDALKKMYMHKNEE--VLGSAGKA----------------------------------------
Spirochaeta_caldaria                GPARDELKKLYMHKNIN--VLGSAGKA----------------------------------------
Treponema_saccharophilum            GPARDELKKMYARKNIE--VLGSAGHALD--------------------------------------
Treponema_sp                        TPARDELKKMYMHKCTD--VLGSAGHALD--------------------------------------
Melioribacter_roseus                GPAREALKELYKHKIVN--VLGSNDKA----------------------------------------
Spirochaeta_thermophila             GPARAELKELYKHKIIN--VLGSEGKA----------------------------------------
Prevotella_multisaccharivorax       KVARNYMIEMYEEKIKD--VLGSENKLANCD------------------------------------
Prevotella_disiens                  GKAREYMTDLYAEKIKN--VLGSDNKLANLD------------------------------------
Fibrobacter_succinogenes            KPARENMKKMYMHKIVD--VLGSNDKL----------------------------------------
Elusimicrobium_minutum              GPARTDLIAMYKEKNQS--VLGSANRVK---------------------------------------
Hippea_maritima                     GPAREELINMYMRKNRE--VLGSANRVY---------------------------------------
Deferribacter_desulfuricans         KPAREELVKMYKHKNIN--VLGSANKA----------------------------------------
Calditerrivibrio_nitroreducens      KPAREELVKLYKHKNIN--VLGSAGKA----------------------------------------
Flexistipes_sinusarabici            KPARNALIDMYKHKNIN--VLGSAEKA----------------------------------------
Pelobacter_carbinolicus             GAGRQELIDLISHKNRN--VVGSADRLA---------------------------------------
Geobacter_lovleyi                   GEARKELIKLIKHKNET--VLGSAGKA----------------------------------------
Desulfurispirillum_indicum          SPARDLLVELIKHKNRE--VLGSAERI----------------------------------------
Trichomonas_vaginalis               GEARTKLMEMYMRKNKE--VLGCAGHAFD--------------------------------------
Entamoeba_dispar                    GPARQALKELYLHKVSDK-VLNSAGKSWY--------------------------------------
Entamoeba_histolytica               GPARQALKELYLHKVSDK-VLNSAGKSWY--------------------------------------
Leptotrichia_goodfellowii           GPAKNYMKDYYKEKIVS--VFGSEGAYKKGTPRK---------------------------------
Leptotrichia_hofstadii              GPAKDYMKEYYKDKIRN--VFGSNGAYKAGAAR----------------------------------
Streptobacillus_moniliformis        GKAKEYMKEYYKSKIID--VFGSQDAYKRGVEVK---------------------------------
Fusobacterium_mortiferum            GAAKEEMKAYYKTKIVD--VFGSEGAYKKGTK-----------------------------------
Fusobacterium_gonidiaformans        GAGQKEMKEYYKTKVQD--VFGSEGAYVKGTK-----------------------------------
Alkaliphilus_metalliredigens        GPGRNAIKKMVQHKIKN--VLKSSNSI----------------------------------------
Veillonella_sp                      GPARDAIKGMVAHKIKN--VLGSSNTL----------------------------------------
Acetonema_longum                    KPAREAIKNMVKHKINA--VLNSSGRL----------------------------------------
Thermosinus_carboxydivorans         KPAREAIKNMVKHKIRN--VLNCSGRL----------------------------------------
Pelosinus_fermentans                KPAREAIKNMVKHKIKN--VLNCSNRL----------------------------------------
Desulfosporosinus_sp                KPAREAIKNMVKHKISN--VLNCSNQI----------------------------------------
Desulfosporosinus_acidiphilus       KPAREAIKNMVKHKIKD--VLNCSNQL----------------------------------------
Acidaminococcus_sp                  GAGRQAIKDMVSHKMRD--VLNSSHRL----------------------------------------
Acidaminococcus_intestini           GAGRQAIKDMVSHKMRD--VLNSSHRL----------------------------------------
Phascolarctobacterium_succinat      KPARQAIQDMVAHKMRD--VLNSSNRL----------------------------------------
Caldicellulosiruptor_kristjans      KDGRDAIKEMVKHKLKN--VLGCAGKAPEILEEIKKNRG----------------------------
Caldicellulosiruptor_lactoacet      KDGRDAIKEMVKHKLKN--VLGCAGKAPEILEEIKKNRG----------------------------
```

```
Tepidanaerobacter_acetatoxydan   GPGRDAIKQMVIHKMRN--VLGCSGKA----------------------------------------
Thermosediminibacter_oceani      GEAREAIKKIVRHKMRN--VLGCSGKI----------------------------------------
Caloramator_australicus          GAAREAIKQVVKDKIVN--VLGCSNTI----------------------------------------
Eubacterium_saphenum             GPARSAIKQMVKHKIKD--VLKASGKR----------------------------------------
Eubacterium_infirmum             GPGRDAITKMVQHKIRN--VLNASNQR----------------------------------------
Acetobacterium_woodii            GPGRSAIKAMVSHKIKN--VLGCSNKR----------------------------------------
Flavonifractor_plautii           KPARANIKEMVKHKLIH--VLGCDGKA----------------------------------------
Oscillibacter_valericigenes      KPARQAIKDMVSHKIVD--VLGCDHKA----------------------------------------
Bryantella_formatexigens         KPARQAVKDMVAHKIQY--VLGSAGKA----------------------------------------
Stomatobaculum_longum            GDGRANVKAIVAHKITE--VLGSNNRI----------------------------------------
Selenomonas_sputigena            APGRDYIRELVEHKIKE--VLGSDGKAAEVMALLK--------------------------------
Megasphaera_elsdenii             ADGRQFVHDIVAHKIKC--VLGSEGKA----------------------------------------
Mitsuokella_multacida            ADGRTYIKELVEHKIKE--VLGSQNSAAGVMELVNANK-----------------------------
Selenomonas_infelix              TDGRSYIKELVTHKIKE--VLGSDGKAAEIQALLNK-------------------------------
Centipeda_periodontii            TDGRSYIKELVTHKIKE--VLGSDGKAPEVQALIDQSK-----------------------------
Megamonas_funiformis             ADGRTNIKAIVSHKIKE--VLGCDGKA----------------------------------------
Anaeroglobus_geminatus           KDGRQYVKDAVIHKIRN--VLGSENKA----------------------------------------
Clostridium_thermocellum         GPARNAIKELVKHKIVN--VLGCDGKA----------------------------------------
Acetivibrio_cellulolyticus       SPARAAIKDVVKHKLIN--VLGCDGKA----------------------------------------
Dictyoglomus_turgidum            GPAREEVKKLVKHKLRN--VLGSSGTV----------------------------------------
Dictyoglomus_thermophilum        GPAREEVKKLVKHKLRN--VLGSSGTI----------------------------------------
Thermotoga_lettingae             GPAREAVKKLVKHKMRN--VLGCSGQA----------------------------------------
Thermotoga_thermarum             GPAREAIKEVVKHKMRN--VLGCSGQA----------------------------------------
Caldisericum_exile               GPARDEIKNLVKHKMRD--VLGSSNTI----------------------------------------
Bilophila_sp                     KPARQAVKDMVQHKIKH--VLGCSGKA----------------------------------------
Bilophila_wadsworthia            KPARQAVKDMVQHKIKH--VLGCSGKA----------------------------------------
Lawsonia_intracellularis         KLARQAVKDMVQHKIKY--VLGCANTI----------------------------------------
Desulfohalobium_retbaense        KPARQAVQDMVQHKIET--VLGSSNTL----------------------------------------
Desulfomicrobium_baculatum       QVARTAVRDMVAHKIRN--VLGSSNKI----------------------------------------
Desulfovibrio_alaskensis         KPAREAVKNMVQHKIRN--VLGCSNKA----------------------------------------
Desulfonatronospira_thiodismut   GPAREAVQEMVQHKIKN--VLGSSGKI----------------------------------------
Desulfovibrio_piger              KPARQAVKDMVAHKIRN--VMGSSGKA----------------------------------------
Dialister_succinatiphilus        KPARNAVKEMVKHKIEA--VLGSAHTI----------------------------------------
Dialister_micraerophilus         KEGRNAVKEMVMHKIEK--VVGSSNSI----------------------------------------
Clostridium_botulinum            GEGRARIEKIVEDKIKM--F-----------------------------------------------
Peptostreptococcus_anaerobius    GAGKEAIKATVEGKIDV--VLGSKDSL----------------------------------------
Arthromitus_sp                   GAAKDGVKKVVLSKIEN--VLGCENSSSK--------------------------------------
Leptonema_illini                 GPVRDAIREIVQHKMKN--VLGSAGRADDVIGAAS--------------------------------
Anaerofustis_stercorihominis     KPARTAVKDMVAHKMQV---FGCAGKGGLK-------------------------------------
Methanocella_arvoryzae           GPAEEAMKEVAKGKMRL---FGSSGKA----------------------------------------
Methanocella_conradii            GPAEDAMKAVAKGKMQL---FGSSGRA----------------------------------------
```

```
Staphylococcus_lugdunensis      GPAREAIKETVKGKIRE---FGTSNRAK---------------------------------------
Staphylococcus_aureus           GPAREAIKETVKGKIKE---FGTSNRAK---------------------------------------
Listeria_grayi                  GPGVDAIIKTVTEKIQE---FGSNGKA----------------------------------------
Brevibacillus_brevis            GPARDAIKEVVKGKMRL---FGSSNRA----------------------------------------
Kyrpidia_tusciae                GPARQAIKETVKAKIRL---FGSNGKA----------------------------------------
Halanaerobium_praevalens        GPGREAIAAKVKEKIKI---LGSDNQAW---------------------------------------
Halanaerobium_hydrogeniformans  GPGRDAITEKVKEKIKM---LGSNDKAW---------------------------------------
Halothermothrix_orenii          GPGRDAIKAKVKEKIKM---LGSQNKA----------------------------------------
Thermoanaerobacter_wiegelii     APGEKAIEEVVKEKIKL---FGCGGRA----------------------------------------
Helicobacter_pylori             SPAQLALKNVVKERMKL---LGSANKI----------------------------------------
Helicobacter_acinonychis        APAQLALKNVVKGRMKL---LGSSNKI----------------------------------------
Thiovulum_sp                    NPAMKFVQSEIVKRMVV---LGSAGKV----------------------------------------
Fervidobacterium_nodosum        GAGKKLVKEVVMNRLEF---LGCAGKA----------------------------------------
Thermosipho_melanesiensis       KEGMEFVKEVVMERLEF---LNTAGKA----------------------------------------
Marinitoga_piezophila           KLAKEYTIKVISDRMEV---LGSAGKAELFK------------------------------------
Mesotoga_prima                  KTPKELVKNVIRERLRL---LGCSNKA----------------------------------------
Aquifex_aeolicus                KPAMEEVKNIVKKRIRL---YGSQGKAELFRV-----------------------------------
Truepera_radiovictrix           GPARDEMKEVVRKRLEL---FKSVGKA----------------------------------------
Thermus_thermophilus            GPAREAVKEVVKSRMEL---FGSVGRA----------------------------------------
Meiothermus_silvanus            GPAREELKQIVKARMEL---FGSAGKA----------------------------------------
Deinococcus_deserti             GPARDVMSKIVEHKLGV---LGSVGKA----------------------------------------
Meiothermus_ruber               GRGRDYLKQVIREKFEL---MGTVGRA----------------------------------------
Thermus_scotoductus             GKGRDYLKQVIREKFEL---MGTVGRA----------------------------------------
Giardia_lamblia                 GPGRDAITEMLIPKIKAFGSAGHAGDYKVVS---------LEEAKAWYK------------------
Bacterium_phylotype_RsD17       GPARTALTNLIITKMKDFGTAGHARDYAPKS---------LEDIKKLYLSK----------------
Eikenella_corrodens             AKTIEAMKGICLARYQAFGCEGMASKIKPIS---------LEKMATRYAKGELNQVVAK--------
Kingella_kingae                 SKTVEAMKQICIDRYLAFGCEGQADKIKPIS---------LEKMATRYAKGELNQIVK---------
Neisseria_shayeganii            AESVKAMKQICLDRYRAFGCEGQAGKIKPVS---------LEKMAERYAKGELAQIVK---------
Laribacter_hongkongensis        KASTDAMRDICIARYEAFGACGQASKIKAVS---------LDAMAASYAKGALTQIIK---------
Acinetobacter_baumannii         AKTVDSMKQICIDRYEAFGTAGNADKIRPIS---------LEKMVDRYK------------------
Acinetobacter_sp                AKTVDAMKQICIERYQAFGTAGNADKIRPIS---------LEAMVDRYK------------------
Marinomonas_mediterranea        TESIKAMKAVCIDRYEAFGCAGQASRITPID---------LEEMAVLYEQGKYSQGRKF--------
Oceanospirillum_sp              KKSMEAMTEICVNRYEAFGTAGNASKITAIS---------LENMAEKYESGELDPKIA---------
Marinobacterium_stanieri        KATMQAMSEICIARYEAFGTAGHASKIKAIS---------LENMSELYMTGELDAKVN---------
Oceanobacter_sp                 KASVAAMSDICIARYEAFGTAGNASKIKPIS---------LDDMFVRYENGELDPKIK---------
Colwellia_psychrerythraea       QVSTNAMYDICKARYEAFNTVGNASKIKPIS---------LDNMFDRYQSGELKALIK---------
Hahella_chejuensis              AVATKAMKEICVARYEAFNAAGQASKIKAMS---------LEAMFQRYARGELDQKIN---------
Alishewanella_aestuarii         QESVKAMADICVARYQAFGCAGQGSKIKPLS---------LDKMVDLYVAGKLTPNIK---------
Alishewanella_jeotgali          QESVKAMADICIARYEAFGCAGQGSKIKPLS---------LDKMVDLYVAGKLTPNIK---------
Rheinheimera_nanhaiensis        QESVKAMMEICIARYEAFNAAGHGSKIKPIS---------LDAMVTLYAAGKLTPNIK---------
Congregibacter_litoralis        KETQVAMRDICIARYEAFGTAGNASKIKVLS---------LDTMQERYDAGELVPEIR---------
```

```
Pseudoalteromonas_marina          AEATKAMTEICVARYNSFGTAGQAGKIKPIS---------LDDMHLKYLSGELAPQVK---------
Alteromonadales_bacterium         AEATKAMTEICVARYNSFGTAGQAGKIKPIS---------LDDMHLKYLSGELAPQIK---------
Idiomarina_baltica                QAATDAMQKICEDRYLAFGCEGQADKIKPIT---------LEKMFQLYEAGSLKAVIK---------
Shewanella_baltica                KASMEAMADICTVRYEAFGCAGQASKIKPLS---------LQAMYKAYQSGALDPKINM--------
Shewanella_violacea               KASMEAMADICTTRYEAFGAAGMGSKIKPKS---------LQAMYKAYQSGELDPQIK---------
Ferrimonas_balearica              QASMVAMAGICKDRYEAFGCAGMGSKIKPLS---------LQAMYKRYLSGELDPKVHI--------
Pseudomonas_mendocina             AKTIVAMRDICIARYEAFGTAGNASKIKPIS---------LEGMYQRYASGELNAKVN---------
Pseudomonas_fulva                 AKTVEAMRDVCIARYEAFGTAGNASKIKPIS---------LEGMFQRYAKGELAAKIN---------
Azotobacter_vinelandii            AKTVEAMRDICIARYEAFGTAGHASKIKPLS---------LEAMFQRYAKGELAAKVN---------
Halomonas_sp                      KETVTAMRDICIARYEAFGTAGNASRIKPIS---------LEAMYERYARGELDPKVK---------
Halomonas_boliviensis             KETVTAMRDLCIARYEAFGTAGNASKIKPIS---------LEEMFLRYERGELAPKVK---------
Chromohalobacter_salexigens       KASVAAMRDVCIARFEAFGTAGQASKIAPID---------LERMFERYASGELAPKVN---------
Marinobacter_algicola             KATMQAMTEVCVARYEAFGTAGNASKIKPVN---------LERMFERYASGELDPKVR---------
Marinobacter_aquaeolei            KATMVAMTDICVARYEAFGCAGQASKIKPLN---------LEQMFERYAAGELDPKVK---------
Thiocystis_violascens             IAATKAMKGICKARYEAFGTAGNASKITALS---------LEVMTNRYAKGELDPQVN---------
Thiocapsa_marina                  IAATKAMKGICKARYEAFGTAGNASKITPMS---------LEAMTDRYAKGELEPKVN---------
Allochromatium_vinosum            TAATKAMKDICKARYEAFGTAGNASKIKVIS---------LEGMTARYAKGELDPRVN---------
Thiorhodococcus_drewsii           TAATKAMKEICVARYEAFGTAGNASKISALS---------LETMTHRYAKGELDPKVN---------
Marichromatium_purpuratum         IAATKAMKDICRTRYEAFGTAGNASKITALS---------LERMTACYDSGELDPRIN---------
Thiomonas_intermedia              IASTKAMKEICKARYEAFGTAGNASKIKPIT---------LDAMVALYEKGALDPKIN---------
Francisella_novicida              AVAKAAMSEICAARYEAFGSAGMASKIKPIS---------LETMFQRYESGELDPIVK---------
Francisella_noatunensis           AVAKVAMSEICQARYEAFGSAGMASKIKPIS---------LETMFQRYESGELDPIVK---------
Kangiella_koreensis               KAAQDAMQAICKARYEAFGTAGNAGKIKAIN---------MEQMALDYKAGKLDPRVK---------
Coxiella_burnetii                 GEAQKAMQAICKARYEAFGTAGQAEKIKIIS---------LENMAGRYKDGELDPRIQNIQPVSSGA
Thiothrix_nivea                   KESTKGMMEICKARYEAFGSAGQASKITPVS---------LEVMFKRYASGELDPKVS---------
Thiobacillus_denitrificans        KEATKGMSDICKARYEAFGAAGQASKIKKVFN--------LEEMQKRYDKGELDPKVN---------
Thioalkalimicrobium_cyclicum      KAAEDAMMEICKARFEAFGCAGHASKIKASG---------LEVMQSRYASGSLDQKVV---------
Halothiobacillus_neapolitanus     AEATKAMKSICQARFEAFGAAGKASLIKPLN---------LDEMHKRYASGSLKQQIK---------
Nitrosococcus_watsonii            KASTAAMKDICQARFEAFGCAGHASKIKPLS---------LEAMFKRYASGELDPRVN---------
Halorhodospira_halophila          KASTEAMKEICKARYEAFGSAGMASKIKPIA---------LETMVERYESGELAPKVS---------
Alkalilimnicola_ehrlichii         KAATEAMKDICKARYEAFGSAGQASKIKPIG---------LMDMVERYASGELDPKVS---------
Rubrivivax_benzoatilyticus        KAATAAARDICRDRFEAFGCAGQASRLG----------RGRG-------------------------
Rubrivivax_gelatinosus            KAATAAARDICRDRFEAFGCAGQASRLG----------RGRG-------------------------
Hydrocarboniphaga_effusa          KAATSAARDVCRLRFEAFGSAGHASRIRPLALDTMAARYDQGDLRAAVH------------------
Dechloromonas_aromatica           KAAVAAASDLCRQRFEVFGSAGQASRIKP---------ISLEKMATTYR------------------
Oxalobacteraceae_bacterium        KDAMTAARALCAERFDAFGCSGQAARIKV---------IGMAQMVQRYPH-----------------
Leptothrix_cholodnii              AAATQAARGICEARFKAFGCEGRAAGIKP---------VSLDRMVVRYR------------------
Methylovorus_glucosetrophus       TAATQGMASICRARFEAFGSAGQASRIRP---------VACAEMARRYREGELTALVQPRQGV----
Methylobacillus_flagellatus       AAATSAMQTLCRERFEAFGSAGRATQIQP---------VPLDEMAQRYVVS----------------
Methylotenera_versatilis          SASTVAMRAICKERYEAFGSAGQAALIKP---------ISCDQMALRYKAGELDAIVKSK-------
Nitrosospira_multiformis          KDATAAARDICKARFEAFGCAGQAAKIKP---------IALDNMAGKYAKGELKAVIK---------
```

```
Gallionella_capsiferriformans      AATTVAMKAICKARYEAFGCAGQASKIKP----------ISLEAMANRYAKGELNAIIK----------
Methylomirabilis_oxyfera           TAATKAMRQICKQRYEQLGSAGNASKIRA----------ISLDDMAHRYLKGELDPRVH----------
Acidithiobacillus_caldus           QASLDAMRDICKQRYEAFGTAGHASKIKV----------IPLDTMVKRYTKGELDPRVTGV--------
Magnetospirillum_magneticum        KPSIAAMVEVCKSRYDAFGTSGQASKIKVVG----------LEDMSALYAKGALAPKVN----------
Magnetospirillum_magnetotactic     KPSIAAMVEVCKSRYDAFGTAGQASKIKVIG----------LEDMSALYAKGALAPKVN----------
Phaeospirillum_molischianum        KQTIKSMSGICKARYEAFGSAGNASKIKVIG----------LEEMTTRYASGELAPKIA----------
Aromatoleum_aromaticum             KPAREAAKLVCKARFEAFGCAGQASRIKPMP----------LEKIAARYKSGELSQVVR----------
Thauera_sp                         KPAREAAKLVCKARYEAFGCAGMASKIKPVA----------LDKIAARYKAGELSQIVR----------
Azoarcus_sp                        KPAREAAKLICKARFEAFGSAGQASKIKPVA----------LDKIAARYKAGELTQVVR----------
Dechlorosoma_suillum               KPAREAAKKICVARYQAFGCEGRAGQIKPIS----------LEKMAERYKKGELNQIVK----------
Accumulibacter_phosphatis          KPAREAAKKICRARFEAFGTAGQASKIKVIA----------LDRMAERYKSGELEQTVR----------
Herbaspirillum_sp                  KPAREAAKLVCKARFLSFGCEGQAAKIKALS----------LDKIAEKYKKGELSQIVQ----------
Herbaspirillum_seropedicae         KPAREAAKLVCKARFLSFGCEGQAGKIKPIS----------LEKIAEKYKSGELAQIVQ----------
Collimonas_fungivorans             KPAREAAKAVVKARFLAFGCEGQAAKIKPVS----------LEKIAEKYKKGELAQIVQ----------
Herminiimonas_arsenicoxydans       KPAKEAAKQVCKARFLAFGCEGQAAKIKPLP----------LEKMAEKYKSGALAQIVQ----------
Polynucleobacter_necessarius       KPAREAAKKVCIARFQAFGSAGQASKIKSIS----------LEKMAELYKSGKLTQIVK----------
Janthinobacterium_sp               KPARTAAEQIVRARMQAFGCEGQASKIKVIT----------MEKMAERYKAGELSQIVK----------
Burkholderia_cenocepacia           KPAREAAKKVCVDRYLAFGCEGQAGKIKPVS----------LDKIAEQYKSGALAQVVR----------
Oxalobacter_formigenes             KAAKEEAKKMCISRYVAFGSAGQASKIKPIP----------LEKMAEKYKKGELAQIVK----------
Ralstonia_eutropha                 KPAREAAKQVCKARYIAFGCEGQAGKIKPVS----------LTDIASQYKSGKLAQVVQ----------
Cupriavidus_taiwanensis            KPAREAAKQVCKARYLAFGCEGQASKIKPVA----------LSEIAQQYKSGKLAQVVQ----------
Cupriavidus_necator                KPAREAAKQVCKARYIAFGCEGQAGKIKPIG----------LTDIAQQYKAGKLSQVVQ----------
Acidovorax_citrulli                KPAREAARQICKQRYIEFGCEGQGAKIKGRS----------LQEMAGLYASGALAQVVN----------
Comamonas_testosteroni             KPAREAAKQVCKARYIEFGCEGQGAKIKGYS----------LEQMAKRYAAGDLAQLVK----------
Acidovorax_radicis                 KPAREAAKQVCKQRYLEFGCEGQGSKIKGLS----------LQAVAAQYAAGTLAQVVN----------
Limnobacter_sp                     KPAREAAKQICKQRYMEFGCEGQGAKIKPIP----------MSEIAQKYAKGELAQIVQ----------
Hylemonella_gracilis               KPAREAAKLVCKARYLEFGCEGQGAKVKSQG----------LAVMAQKYAKGQLAQVVH----------
Methyloversatilis_universalis      KPAREAAKAVCKQRYLEFGCEGQAPKIKAIP----------LDEMARRYAAGQLAQTVN----------
Alicycliphilus_denitrificans       KPAREAAKAICKQRYLEFGCEGQGAKIKGHT----------LEQMAQKYAAGALGQVVN----------
Delftia_sp                         KPAREAAKQICKARYIEFGCEGQGAKIKGYT----------LEQMAAKYAAGSLQQVVN----------
Delftia_acidovorans                KPAREAAKQICKARYIEFGCEGQGAKIKGYT----------LEQMAAKYAAGSLQQVVN----------
Ralstonia_pickettii                KPAREAAKQICKARYIQFGCEGQAGKIKPIS----------LSVMAQKYQSGDLAQVVQ----------
Variovorax_paradoxus               KPAREAARLICKQRYIEFGCEGQGAKIKGET----------LQVVAAKYAKGELAQEVV----------
Hydrogenophaga_sp                  KPAREAAKQICKQRYIEFGCEGQAGKIKGDT----------LQVVAGKYAKGELAQVVN----------
Ramlibacter_tataouinensis          KPAREAAKQICKQRYIEFGCEGQAGKIKGRP----------LQVVAAQYEKGELAQTVN----------
Polaromonas_naphthalenivorans      KPAREAAKAICKQRYIEFGCEGQAGTIKGQS----------LSVVAAQYAKGELAQVVQ----------
Methylibium_petroleiphilum         KPATAAAKSICKQRYVEFGCEGQAPKIKGET----------LSVVAAKYAKGELAQVVQ----------
Achromobacter_xylosoxidans         KPARAAAKAICVARYTEFGTAGNASKIKALP----------LTEIAAQYASGKLAQVVQ----------
Achromobacter_arsenitoxydans       KPARAAAKAICVARYTEFGTAGNASKIKALP----------LTDIAAQYASGKLAQVVQ----------
Bordetella_petrii                  KPARAAAKAICVQRYTQFGTAGNASKIKPLP----------LTEMAQQYAAGKLTQVVQ----------
Bordetella_pertussis               KPARAAAKAICVQRYTQFGTAGNASKIKPLP----------LADMAQQYAAGKLAQVVQ----------
```

```
Advenella_kashmirensis            KPAREAAKKICVARYQEFGTAGNASKIKPIA---------LSEMAAAYAAGKLSQQIK---------
Pusillimonas_sp                   KPAREAAKLICKQRYEQFGTAGNASKIKPIS---------LSEMAKRYAAGELAQVVQ---------
Alcaligenes_faecalis              KPAREAAKKVCVARYQQFGAAGQASKIKAIP---------LQEIARQYSTGELSQIVQ---------
Taylorella_equigenitalis          KPAREAAKQLCISRYEAFGTAGNASKIKPVE---------LSEMAQKYASGELVQKVN---------
Parasutterella_excrementihomin    KAARAASVEMCKSRYIAFGCEGQASKIKPLP---------LPEMARLYSESVLRQKVND--------
Burkholderiales_bacterium         KAARAASVEMCKSRYIAFGCEGQASKIKPLP---------LPEMARLYSEGVLRQKVND--------
Sutterella_wadsworthensis         KAARKAAENLCRERYLAFGCEGQGAKIKPIP---------LEKIAALYADGTLRQHVL---------
Xanthobacter_autotrophicus        AAAMEEAKKVCINRFEAFGAAGKADKIRPIE---------LDDMAKRYASGELAQVVH---------
Magnetococcus_marinus             RPAEDAAYAVCKARYEAFGAAGNASKIKVIT---------MEDMAKRYASGELDPKVN---------
Nostoc_punctiforme                KPSITYMQKVCAERYQQFGTAGNASKIKQIS---------LEDFAAKYAKGELNVVTKSAAKV----
Anabaena_variabilis               KPSITYMQKVCAERYVQFGTAGNASKIKQVS---------LETFAAKYAKGELNAISKSAAKV----
Microcystis_aeruginosa            KPSITYMQKVCADRYQQFGTAGNASKIKQMS---------LDDYAAKYAKGQLTQVSKKVVAV----
Lyngbya_sp                        KPSIAKMKAVCADRYQQFWAAGNASKIKVIT---------LDDFAAKYAKGELSATSKSAVAV----
Arthrospira_platensis             KPSIKYMQKVCADRYQQFGTAGNADKIKVQT---------LDEFAAKYASGALSATSKKAVAV----
Oscillatoria_sp                   KPSIKYMQKVCSDRYQSFGAAGHGTNIKQIS---------LDDFAAKYAKGELKAISKSAVTA----
Cyanothece_sp                     KPSMKYMQKVCSDRYQEFWTAGNASKIKQQG---------LDEYAAKYAKGELSAGTKKAVAV----
Synechococcus_elongatus           KPAIKYMKQVCVDRYVAFNTAGQASKIKQEG---------LDEFAAKYAKGELRAQTKVFA------
Prochlorococcus_marinus           KPARKYMKQVCLDRYQQFWCAGQASKIKQES---------TNYYSGLYAKGTLDPKAAVAV------
Paulinella_chromatophora          KPARKYMKQVCLDRYQQFWCAGNASKIHQRD---------INYYAGLYAKGELDPKTAVAA------
Synechococcus_sp                  KPSIKEMQKVCAERYIAFGCAGHGTKVPVVT---------LDEMAAKYASGALAAQVKKTVAV----
Gloeobacter_violaceus             IPSMKYMLKVCRDRYVEFGSAEQASKIKQVS---------LDEMAKRYIA---ASTMKKAVTV----
Sinorhizobium_meliloti            KPAIAHMTEVCKERFVAFRAAGQASKIRVLR---------LPEMAKRYAAA----------------
Stappia_aggregata                 KPARDAMQKLCVERLEAFNTAGKASKIKKIIT--------LADMAKRYQTGELDPKIA---------
Labrenzia_alexandrii              KPAREAMQKLCVARLEAFNTAGQASKIKKIVT--------LSDMAARYQNGSLDPKIA---------
Polymorphum_gilvum                KPARDAMTKLCTERLEAFNTAGQASKIRKVLT--------LAEMAARYKSGALDPKIA---------
Bradyrhizobium_sp                 KPAMEAMTKLCKQRLQEFNTAGQASKFKKVLT--------PAEMAKRYAKGELDPKVA---------
Agrobacterium_radiobacter         KPAMAALTKLCKERFEQFGTAGHATRIKPLP---------VSDMAKRYKAGSLDPIFS---------
Rhizobium_sp                      KPAMAALTKLCKERFEQFGTAGHATRIKPLP---------VSDMAKRYKAGSLDPIFS---------
Brucella_melitensis               KPAMAAMTKLCKERFEQFGTAGHAASIRPIP---------LSEMAKRYRDGSLDPKFS---------
Brucella_suis                     KPAMAAMTKLCKERFEQFGTAGHAASIRPIP---------LSEMAKRYRDGSLDPKFS---------
Ochrobactrum_anthropi             KPAMAAMTKLCKERFEQFGTAGHAASIRPIP---------LSEMAKRYRDGSLDPKFS---------
Sinorhizobium_fredii              KPAMTALSKLCRERFEAFGTAGRASSIHVVP---------LAEMAKRYRAGSL--------------
Mesorhizobium_amorphae            TPAMAAMRKLCKERFEQFGTAGNAQKIKPLP---------VAEMAKRYKSGSLDPKFG---------
Mesorhizobium_loti                TPAMAAMRKLCKERFEQFGTAGNAPKIKPLP---------VSEMAKRYKSGSLDPKFG---------
Methylobacterium_nodulans         KPAMEAMTKLCKQRFEEFGTAGHAGKIRPIS---------LSEMAKRYASGKLDPSFGSKQKAAAE-
Methylobacterium_radiotolerans    KPAMEAMTKLCKQRFEEFNTAGKGSKIRPIS---------VAEMAKRYASGALDPKIGPQ-------
Phenylobacterium_zucineum         KPAKEAMRKVCVQRFEEFGTAGHASKIKPLP---------MSAMAKRYASGELAPKIGLTKVAAE--
Caulobacter_sp                    KPAKEAMRKVCQARFVEFGSAGHADKVRALS---------TATMAKRYTAGELDAKFGAAKKAAAA-
Caulobacter_crescentus            KPAKEAMRKVCQARFVEFGSAGHADKIKPMS---------TATMAKRYASGELHAKFGASAAKAAAE
Paracoccus_denitrificans          IPAMKELTALCRDRFERFGTAGHASKIRVIP---------MDEMARRYASGALDPAITGAKAA----
Paracoccus_sp                     IPAMKELTALCRDRFERFGTAGHAGRIRVIP---------MDEMARRYASGALDPAITGARAA----
```

```
Pelagibaca_bermudensis           APAMEELRALCRDRFERFGTAGQASTIRPVS---------LDDMASRYASGALDPQIAAANAA----
Citreicella_sp                   APAMEELRKLCRDRFERFGTAGQASKIKPVS---------LDDMASRYASGALDPRIAAADAA----
Rhodopseudomonas_palustris       KPAMDAMRDLCRLRFEQFGTAGHASKIKVIP---------LSEMAKRYKSGALDPQIGQVARAAE--
Starkeya_novella                 KPAMDAMRDLCRERFEQFGTAGHAAQIKVLP---------LAAMAKRYASGALDPVIGGAQAAAE--
Azospirillum_amazonens           KPAMDAMKALCRDRFEQFGAAGNASRITPLP---------MAAMAKRYATGALDPHLGSTPVAAE--
Rhodobacter_sphaeroides          KPAMDAMRDLCKQRLEAFGTAGQAGKIRIIP---------MDDMAKRYASGALAPKTA---------
Aurantimonas_manganoxydans       IPAMDALKTLCLDRYERFGCAGQASKIRVLS---------LDAMAARYAGGELDPRIEAHAVAA---
Octadecabacter_arcticus          MPGREAMSKVCRERYEQFGTAGHAQKIKPVA---------LTEMAKRY-------------------
Sulfitobacter_sp                 APGVEAMSAVCFDRFERFGCAGMASKISAIP---------LSDMAKRYQSGSLRPFLGC--------
Rhodospirillum_photometricum     KPSMDAMEKVCRERYEQFGAVGMASKIKPVP---------LAEMAKRYASGSLAPKVQ---------
Pirellula_staleyi                KPARTAMKEVCKARMVQFGQAGNASKLMAKI------------KA----------------------
Blastopirellula_marina           KPARAAMKQVCVDRMTAFGQAGMAAKMRDAK------------LI----------------------
Rhodopirellula_baltica           KPARAAMKDVCVARMTAFGQAGNGAKLRATL------------GAAV--------------------
Koribacter_versatilis            KPAREAMKKVVAQRMKEFGQAGHAGDYAPIS---------LSDYASKYAANALVAAR----------
Solibacter_usitatus              KPARDAMKKVVALRMTQFGQAGHAKDYTPIP---------LDEMAKRYQP---VTA-----------
Anaerolinea_thermophila          KIFMAEYAKFIAHKNEKLGSAGRLPEVRALLGK----------------------------------
Diplosphaera_colitermitum        KVYIEEYAKFIASRNTLLGSAGQLDDLRASLKK----------------------------------
CONSERVATION
```

Aldolase Sequence Logos

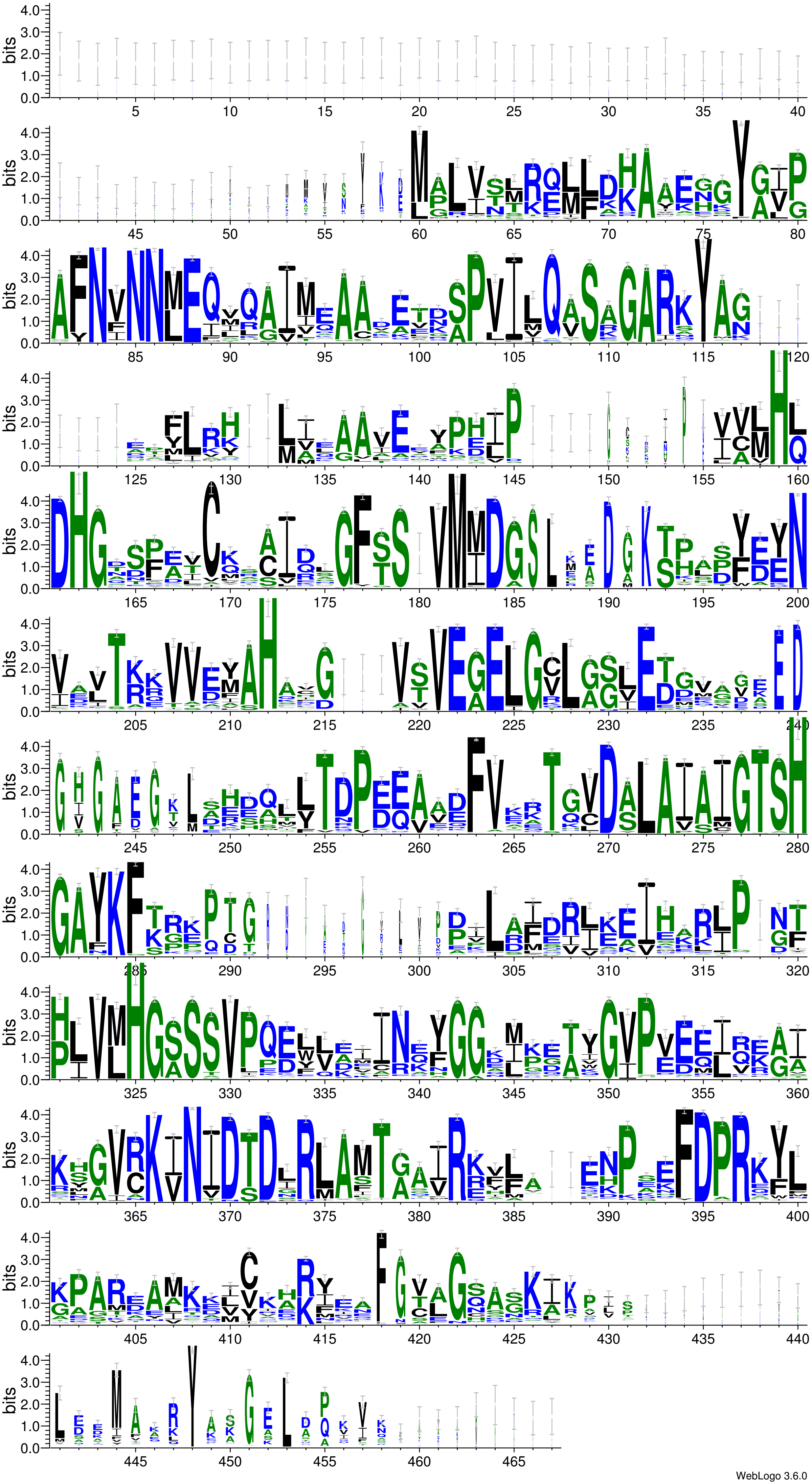

Figure S2. Full-length, unmodified image of SDS-PAGE shown in Fig. 2

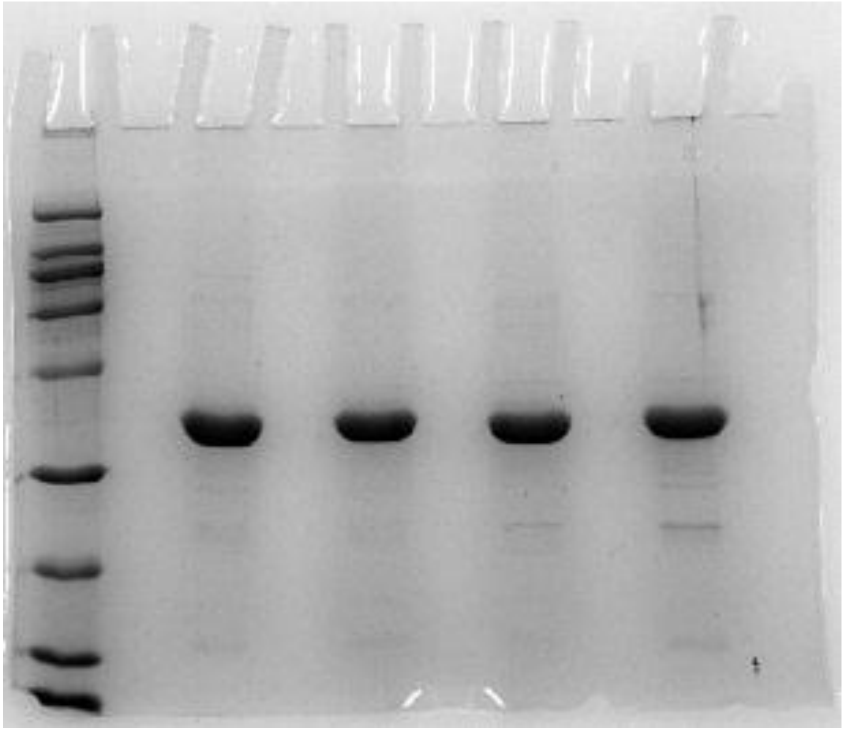

Figure S3. 2D ligand interacting plot of representative molecules docked into the proposed binding site. CAS numbers are A) 51-75-2, B) 26675-46-7, C) 1984-15-2 and D) 58-39-9.

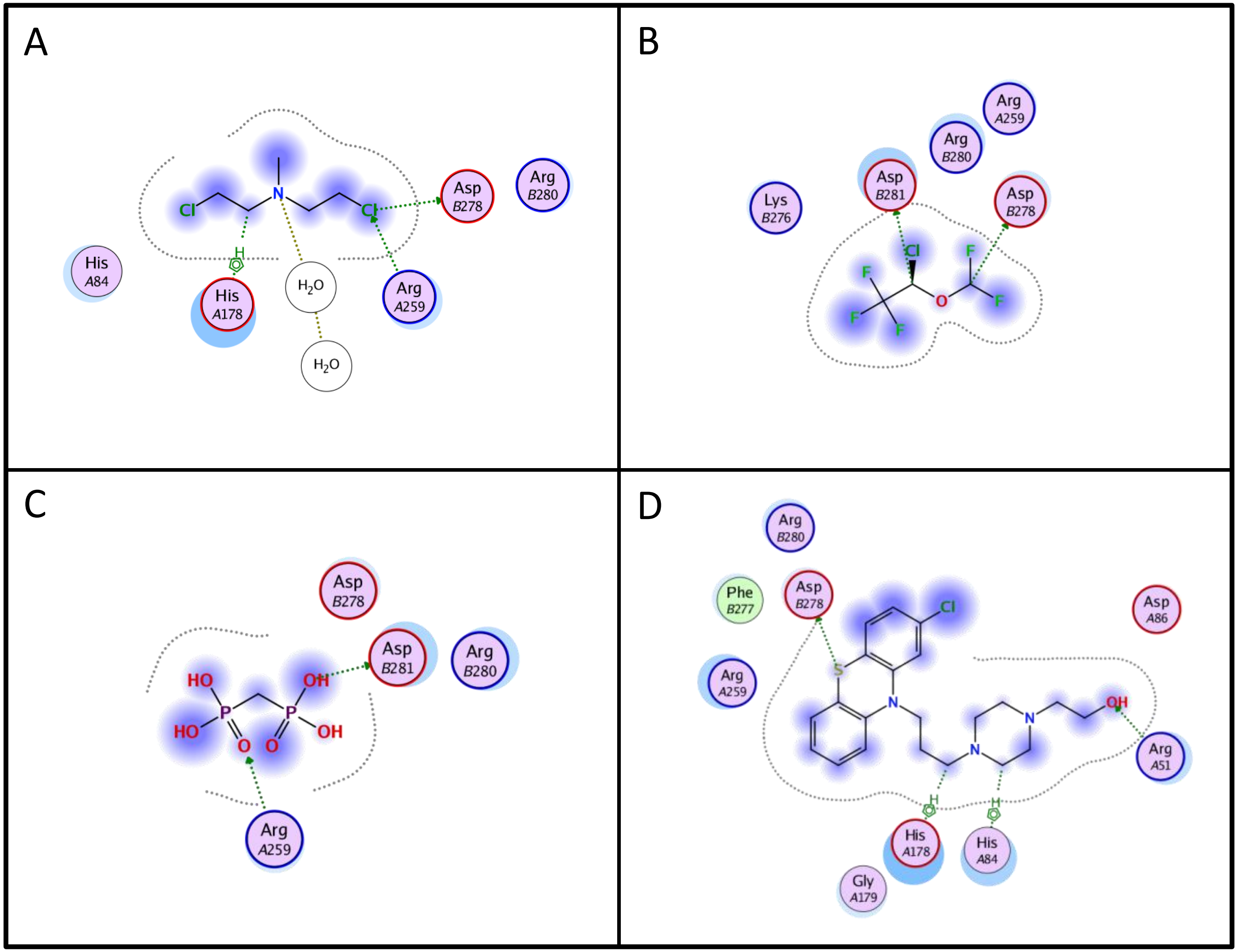

Figure S4. 3D ligand interacting plot of a representative molecule (CAS number 58-39-9) docked into the proposed binding site.

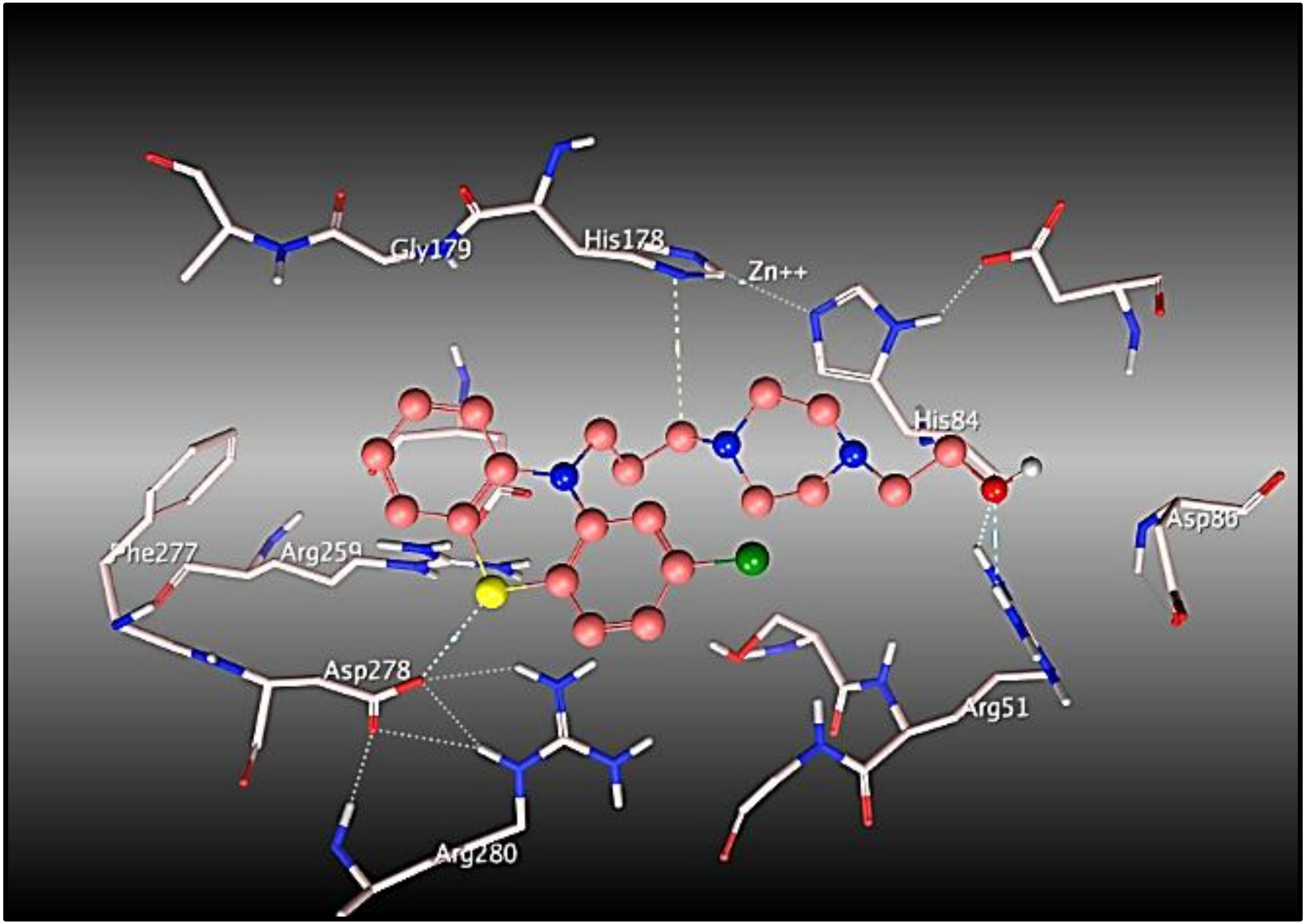

Table S1. Potential binding sites predicted by SiteFinder (MOE), ranked by the number of hydrophobic contact atoms in the receptor (column Hyd, descending order). The Size column indicates the number of contact atoms in the receptor. Propensity for Ligand Binding (PLB) score is based on the amino acid composition of the pocket, based on statistical models. The Side column indicates the number of sidechain contact atoms in the receptor. The Residues column indicates the residues that make up the calculated site in the format chain:residue-name.

Size	PLB	Hyd	Side	Residues
72	3.43	5	43	1:(ALA22 ASN24 GLN48 SER50 ASP83 HIS84 SER177 HIS178 GLY179 LYS182 HIS210 GLY211 SER212 SER213 ASN253 VAL254 ASP255 SER256 ARG259)2:(ARG280)3:(ZN326)
41	1.40	10	40	2:(ASN24 GLN48 SER50 ASP83 HIS84 HIS210 GLY211 SER212 SER213 SER214 ILE241 ILE252 ASN253 VAL254 ASP255 SER256 ASP257 PHE301)
44	1.32	15	38	1:(GLN32 GLY33 MET35 LYS36 VAL39 GLN40 LYS72 HIS73 ARG287 ASP288 THR291)2:(ASP58 TYR61)
14	0.88	10	15	2:(ILE174 GLY175 ILE193 VAL196 MET209 SER240 HIS243 ALA244 GLU247)
43	0.79	13	18	1:(LEU139 GLU143 VAL146 GLN147 ASN148 THR149 VAL150 LEU152 GLY175 THR176 SER177 HIS178 TYR181 LYS182 PHE183 LYS184 ILE189 ARG190)
19	0.70	14	20	1:(ARG267 PHE270 VAL271)2:(TYR226 GLY227 ARG267 PHE270 VAL271)
44	0.70	10	33	1:(TYR56 ASP58 TYR61)2:(GLN32 GLY33 MET35 LYS36 LYS72 HIS73 ARG287 ASP288 THR291)
29	0.46	4	15	2:(MET209 HIS210 GLY211 SER212 SER213 SER214 GLY235 VAL236 PRO237 SER240 ILE241 ILE252 ASN253)
20	0.40	5	17	2:(ALA13 ARG14 LYS15 LYS17 SER200 ASP201 ILE245 GLY246 GLU247 GLY248)
18	0.25	9	15	1:(GLU186 SER187 SER214 VAL215 PRO216 LYS217 LYS220 ALA233 VAL234 GLY235)
14	0.20	10	29	1:(ASP278 PRO279 ARG280)2:(ASN24 VAL25 ASN26 GLN30 ILE34 VAL254 ASP255 SER258 ARG259 ILE290)
20	0.18	10	15	2:(LEU152 THR153 VAL172 ALA173 ILE174 GLY175 ALA192 ARG195 VAL196 ILE199)
27	-0.18	10	14	2:(ASP218 VAL219 MET222 MET260 ALA261 GLY264 ALA265 ALA289 GLU292 MET293)
29	-0.30	17	23	2:(SER214 PRO216 ASP218 VAL219 VAL236 PRO237 ILE238 MET293 PRO296 LYS297 ALA300)
35	-0.35	5	12	2:(HIS84 VAL172 ALA173 ILE174 GLY175 MET209 HIS210 GLY211 SER212 SER240)
18	-0.49	0	17	1:(HIS84 ASP105 SER107 HIS108 GLU135 LEU139 GLY140 GLY141 HIS178)
22	-0.63	13	17	2:(LEU3 LYS63 CYS66 GLU67 LEU70 ILE80 LEU97 GLY98 PHE99)
7	-0.74	5	9	1:(PHE183 LYS184 SER185 ILE189 ARG190)
15	-0.75	7	13	1:(ARG280 ASP281 GLY284 ARG287)2:(GLY52 LYS55 TYR56)
12	-0.76	8	11	1:(ARG51 GLY52 LEU54 LYS55 MET59 MET93)
13	-0.82	6	15	2:(ARG7 HIS126 GLY166 VAL167 ASP168 ILE205 PRO206 TYR322)
10	-0.87	4	9	2:(MET1 PRO2 LEU3 VAL312 VAL313 SER314 GLU317)
15	-0.87	5	8	1:(VAL122 ALA123 HIS126 GLY166 TYR322 LYS323)
12	-0.89	5	10	1:(SER107 HIS109 PRO110 PHE111 ASN114 LEU136 GLY137 GLN151)
11	-0.96	10	16	1:(ASP188 ILE189 ARG190 LEU191 ALA192 ILE193 SER240 HIS243)
15	-0.99	6	10	1:(GLY52 LYS55 TYR56)2:(ARG280 ASP281 ARG287)
11	-1.12	3	8	2:(GLY175 LEU191 ILE193 SER240 HIS243 ALA244)
